# Monitoring Daily Sleep, Mood, and Affect Using Digital Technologies and Wearables: A Systematic Review

**DOI:** 10.3390/s24144701

**Published:** 2024-07-19

**Authors:** Robert Hickman, Teresa C. D’Oliveira, Ashleigh Davies, Sukhi Shergill

**Affiliations:** 1 Institute of Psychiatry, Psychology & Neuroscience, King’s College London, 16 De Crespigny Park, London SE5 8AF, UK; robert.1.hickman@kcl.ac.uk; 2 National Institute for Health Research, Maudsley Biomedical Research Centre, South London and Maudsley National Health Service Foundation Trust, London SE5 8AF, UK; 3 School of Psychology and Life Sciences, Canterbury Christ Church University, North Holmes Road, Canterbury CT1 1QU, UK; 4 Kent and Medway Medical School, Canterbury Christ Church University and the University of Kent, Canterbury CT2 7NZ, UK; ashleigh.davies@kmms.ac.uk

**Keywords:** sleep, circadian rhythms, affect, mood, emotions, actigraphy, prospective, systematic review

## Abstract

**Background:** Sleep and affective states are closely intertwined. Nevertheless, previous methods to evaluate sleep-affect associations have been limited by poor ecological validity, with a few studies examining temporal or dynamic interactions in naturalistic settings. **Objectives:** First, to update and integrate evidence from studies investigating the reciprocal relationship between daily sleep and affective phenomena (mood, affect, and emotions) through ambulatory and prospective monitoring. Second, to evaluate differential patterns based on age, affective disorder diagnosis (bipolar, depression, and anxiety), and shift work patterns on day-to-day sleep-emotion dyads. Third, to summarise the use of wearables, actigraphy, and digital tools in assessing longitudinal sleep-affect associations. **Method:** A comprehensive PRISMA-compliant systematic review was conducted through the EMBASE, Ovid MEDLINE(R), PsycINFO, and Scopus databases. **Results:** Of the 3024 records screened, 121 studies were included. Bidirectionality of sleep-affect associations was found (in general) across affective disorders (bipolar, depression, and anxiety), shift workers, and healthy participants representing a range of age groups. However, findings were influenced by the sleep indices and affective dimensions operationalised, sampling resolution, time of day effects, and diagnostic status. **Conclusions:** Sleep disturbances, especially poorer sleep quality and truncated sleep duration, were consistently found to influence positive and negative affective experiences. Sleep was more often a stronger predictor of subsequent daytime affect than vice versa. The strength and magnitude of sleep-affect associations were more robust for subjective (self-reported) sleep parameters compared to objective (actigraphic) sleep parameters.

## 1. Introduction

Accumulative evidence indicates a close and complex connection between sleep and affective functioning. Extant studies across naturalistic and experimental settings show compromised sleep, increases the prevalence of negative emotions, reduces positive mood states and general wellbeing, dampens emotional arousal, impairs affect regulation, and elevates overall negative affective outcomes [1,2,3,4,5,6,7,8]. Affective states (positive and negative) and affective variability also impact sleep-wake patterns and behaviour bidirectionally [1,9,10,11]. Sleep loss also perturbs underlying brain regions and connectivity (both subcortically and cortically) that subserve emotion regulation, expression, reactivity, discrimination, and affective and cognitive processing [12,13,14,15,16,17,18,19]. Sleep deprivation after only one night, for example, amplifies amygdala reactivity to negative emotional stimuli and reduces prefrontal connectivity through top-down dysregulation [20]. Sleep, especially deep or rapid eye movement (REM) periods, is linked to affective homeostasis and appears restorative in resetting emotional reactivity [2,13,21,22].

Sleep and circadian dysrhythmia are implicated in the pathophysiology and psychopathology of nearly all psychiatric disorders [23,24,25] and sleep difficulties are pervasive among the general population [26]. Sleep and rest-activity disturbances, for example, are particularly prevalent in all affective disorders, schizophrenia, and psychosis spectrum disorders [23,27,28,29]. Sleep problems are often prodromal factors for worsening mood symptoms or the precipitation of manic, depressive, or hypomanic episodes. Interventions targeting core sleep-circadian dimensions also improve symptom burden and prognosis [30,31,32]. One such intervention, Interpersonal and Social Rhythm Therapy (IPSRT), has been shown to improve psychopathology and mood symptoms in bipolar disorder through core stabilisation of daily rhythms and sleep-wake routines [33,34,35,36]. Night-shift workers are also vulnerable to circadian and sleep-wake misalignment, which confers risk for affective disorders [37], poorer mental health [38], and worsened mood or depressive symptoms. A recent meta-analysis, for example, found that night shift workers were around 40% more likely to develop depression than those working day schedules [39], while the systematic review from D’Oliveira and Anagnostopoulos [40] provides support for an association between shift work and affective disorders.

A growing body of research has explored the interrelationship of sleep and affect sampled in naturalistic settings. Recent emerging technologies and ambulatory techniques have facilitated fine-grained, longitudinal data collection, which is more ecologically valid [41]. Prospective monitoring through smartphone-based ecological momentary assessment (EMA) or experience sampling method (ESM), for example, is non-invasive, cost-effective, and enables researchers to closely predict complex sleep-affect associations in real-time and with higher levels of granularity. Given the rapid emergence of naturalistic studies, there is a need to evaluate and update evidence on the day-to-day fluctuations and temporal patterns of sleep and affect.

This systematic review aimed to address three main research questions: (1) First, to review updated evidence on the interplay of daily sleep and affective experiences (mood, affect, and emotions). The search strategy was broadened and included studies published until the end of May 2024, thus comprehensively updating and building on prior work since Konjarski et al. [42], Ong et al. [43], and ten Brink et al. [44] in a fast-moving and expanding field of sleep-affect research. (2) Second, to understand how sleep-affect associations differ and compare across age groups, affective disorder diagnoses (bipolar, depression, and anxiety), shift workers, and the consideration of situational factors (i.e., the context of daily mood assessments). This broadens the scope and inclusion criteria of prior sleep-affect reviews, which exclude bipolar-spectrum disorders and shift worker samples (e.g., due to core circadian misalignment or non-traditional sleep schedules that may impact affective experiences.; (3) Third, to comprehensively review the use of wearables, actigraphy, and digital tools to capture day-to-day fluctuations in sleep-affect patterns.

## 2. Materials and Methods

### 2.1. Search Strategy and Selection Criteria

A PRISMA-compliant systematic review [45] was conducted (Table S1) in four electronic databases until 28 May 2024: EMBASE (via Ovid), Ovid MEDLINE(R), PsycINFO (via Ovid), and Scopus (Elsevier, Amsterdam, The Netherlands). Ovid MEDLINE(R) included references from ‘Epub Ahead of Print, In-Process & Other Non-Indexed Citations and Daily’ as recommended by Bramer et al. [46]. Backward and forward citation searches were also utilised to identify relevant studies. An updated search was completed on 28 May 2024 and followed Bramer and Bain [47] and Cochrane guidelines for updating systematic searches within 12 months of publication. The systematic search strategy (see Table S2 for the complete search strings) followed PRESS recommendations [48], and syntax was adjusted for each database. The Yale MeSH Analyzer tool helped identify key search terms and phrases [49]. The full inclusion and exclusion criteria are listed in Table 1.

### 2.2. Data Extraction and Analysis

**Figure 1:**
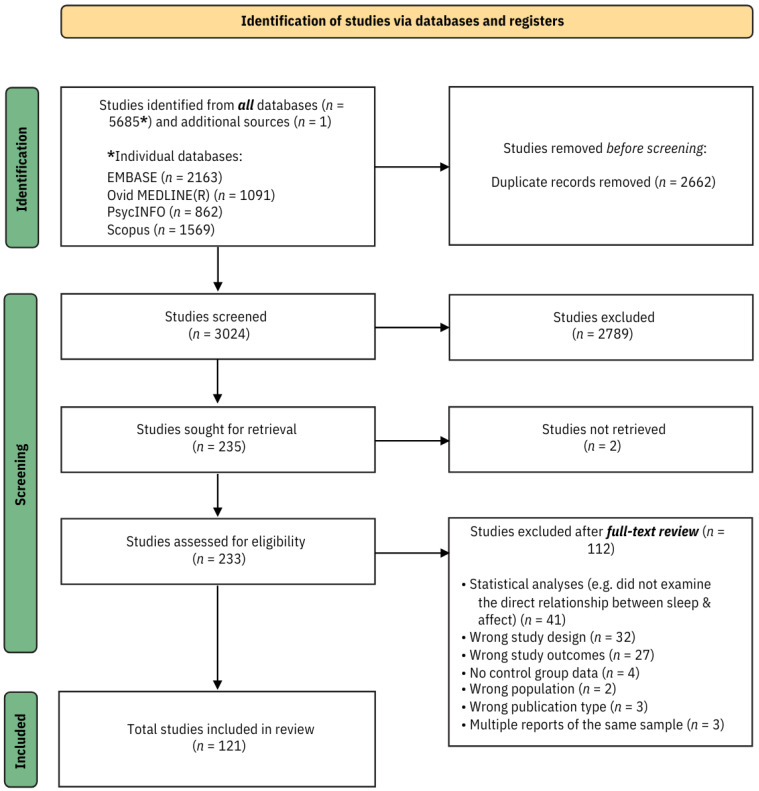
PRISMA flow diagram outlining the study selection process. Additional sources (*n* = 1) refers to one study that was identified through forward and backward citation searching.

Retrieved records were exported and de-duplicated in EndNote following Bramer and Bain [47], Bramer et al. [50] guidelines. De-duplicated records were screened on Rayyan by title and abstract, and potentially relevant studies were retrieved for full-text article screening. A total of 112 studies were excluded at full-text screening from the review (Table S3). The PICO-based (Population, Intervention, Comparison, Outcome) taxonomy of reasons was used to exclude articles from the systematic review [51]. Data extraction followed a standardised data extraction form (protocol available upon request). Categories of data and information extracted from identified records are outlined in Table S4, and the search and screening process is shown in Figure 1. Screening and data extraction were performed by one author (R.H.). Two student reviewers (blinded) also independently screened all records at the title, abstract, and full-text screening stages. Data extraction was verified by another independent (blinded) student reviewer. Discrepancies at any stage were discussed, checked, and resolved by a senior researcher (T.D.). Given the high heterogeneity across study designs and samples, this review adopted a narrative synthesis to summarise the findings.

### 2.3. Risk of Bias

Systematic risk of bias was evaluated using the National Heart, Lung and Blood Institute (NHLBI) Quality Assessment Tool for Observational Cohort and Cross-Sectional Studies, in line with prior reviews from Konjarski, Murray, Lee and Jackson [42] and ten Brink, Dietch, Tutek, Suh, Gross and Manber [44]. Two raters (R.H. and A.D.) independently reviewed study quality and risk of bias using the NHLBI tool. Discrepancies after blinded review were then discussed between the two authors (R.H. and A.D.) and resolved by consensus. In total, 63 records (52.1%) were rated as ‘Good’ (minimal or low risk of bias), and 58 records (47.9%) were assigned a ‘Fair’ grade (moderate risk of bias). Study quality ratings are outlined in Table 2. No records were excluded from the final data synthesis.

## 3. Results

### 3.1. Literature Search

A total of 5686 records were identified across all databases and sources. After de-duplication using Bramer and Bain [47], Bramer et al. [50] guidelines, 3024 records were screened based on title and abstract, of which 233 were then assessed for full-text screening. A final 121 studies met the full inclusion criteria (see PRISMA flow diagram, Figure 1).

### 3.2. Description of the Included Studies

**Figure 2:**
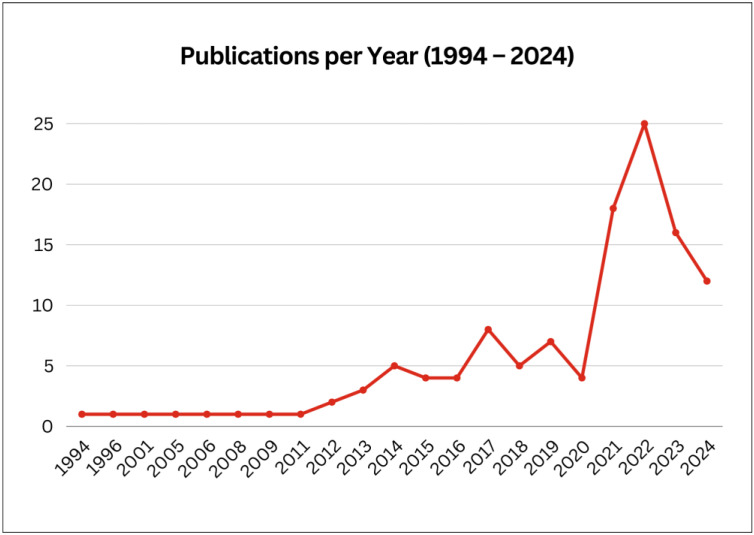
Chart of studies included in this review (*n* = 121) published per year from 1994 to 2024. Since 2021, there have been 71 studies published (up to the end of May 2024).

Studies were published between 1994 and 2024. An overview of included studies is summarised in Table 2, with sample characteristics, study quality ratings, and daily outcomes of sleep, mood, and affect reported. As evidenced in Figure 2, there has been a rapid emergence of published research (particularly from 2021 onwards) utilising naturalistic study designs to monitor day-to-day sleep, mood, or affect associations.

#### 3.2.1. Study Location

Studies were predominantly from the United States (*n* = 72; 59.5%) but represented a wide geographical area, including North and South America, Europe, the Middle East, and Asia-Pacific regions; China (*n* = 7; 5.8%), Germany (*n* = 7; 5.8%), The Netherlands (*n* = 5; 4.1%), Canada (*n* = 4; 3.3%), United Kingdom (*n* = 4; 3.3%), Israel (*n* = 3; 2.5%), Australia (*n* = 2; 1.7%), Hong Kong (*n* = 2; 1.7%), Japan (*n* = 2; 1.7%), Belgium (*n* = 1; 0.8%), Chile (*n* = 1; 0.8%), Denmark (*n* = 1; 0.8%), Finland (*n* = 1; 0.8%), Hungary (*n* = 1; 0.8%), Singapore (*n* = 1; 0.8%), Spain (*n* = 1; 0.8%), Switzerland (*n* = 1; 0.8%), and Taiwan (*n* = 1; 0.8%), and studies with two or more participating countries (*n* = 4; 3.3%).

#### 3.2.2. Participant Characteristics

Sample sizes ranged from 19 to 2804, with most studies including at least 50 participants (*n* = 109; 90.1%). Studies that involved adult populations (18 years of age or older) were most common (*n* = 103), followed by children and/or adolescent samples (<18 years of age based on WHO classifications) (*n* = 27). A small proportion of these studies had overlapping samples, which included both adult and older adult (65 years or older) populations (*n* = 15) or samples with both adults and children or adolescents (*n* = 9). Five studies explicitly involved only older adult samples [9,57,93,145,166] and eighteen studies included only children and/or adolescent samples [56,59,65,66,73,74,77,112,113,142,143,144,151,157,169,170,173,177].

Most studies involved healthy populations (*n* = 97; 80.2%) but seventeen (14%) had clinical samples with diagnosed affective or mood disorders; bipolar disorder (*n* = 7) [60,61,91,92,136,140,141], major depression, or depressive disorder (*n* = 11) [59,79,83,92,106,111,165,172,173,175,176], and anxiety disorder (*n* = 4) [59,106,165,173]. Eight studies (6.6%) investigated shift workers [54,55,88,98,107,118,150,162], mostly among healthcare staff (medical residents, *n* = 4 and nurses, *n* = 2). One study [98] included both standard work schedules and a subsample of shift workers.

#### 3.2.3. Length of Data Collection

The period of assessment varied across studies, with 1–2 weeks being the most common (66% of all records). In total, 116 studies (96%) collected data for under 2 months (ranging from 3–56 days), with the remaining studies [55,88,107,134,141] capturing long-term sleep variability and affect (ranging from 3 months to 2 years), often over multiple waves. Table S6 in the Supplementary Materials summarises overall study length and measurement frequency.

#### 3.2.4. Sleep Measures

**Figure 3:**
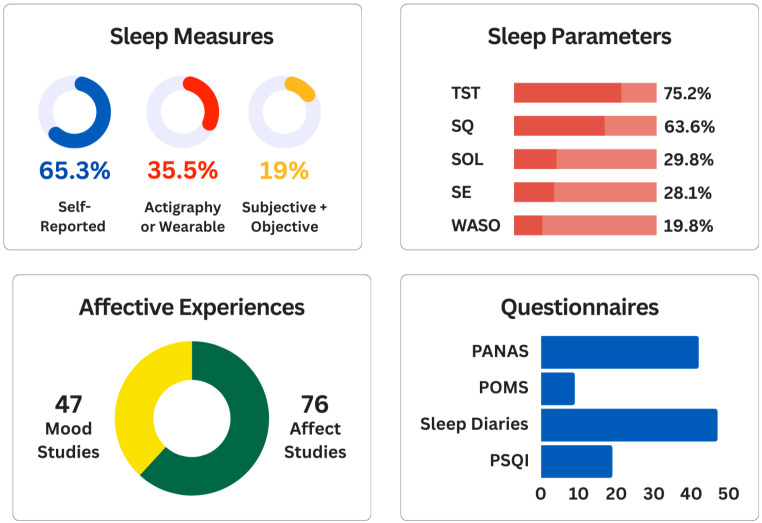
Proportion of studies with subjective or objective sleep markers and the affective domains assessed. Overview of the most frequent self-report measures and sleep variables analysed (PANAS = Positive and Negative Affect Schedule; POMS = Profile of Mood States; PSQI = Pittsburgh Sleep Quality Index; TST = sleep duration; SQ = sleep quality; SOL = sleep onset latency; SE = sleep efficiency; WASO = time awake after sleep onset).

The most assessed sleep parameters were sleep duration (TST; 75.2%), sleep quality (SQ; 63.6%), sleep onset latency (SOL; 29.8%), sleep efficiency (SE; 28.1%), and time awake after sleep onset (WASO; 19.8%). In total, 79 studies (65.3%) analysed only subjective (self-report) sleep measures (see Figure 3). Just over a third of identified studies had actigraphy or wearables (*n* = 43; 35.5%) to record sleep parameters, but only 23 of these (19%) [55,57,60,64,71,75,86,90,96,106,110,113,117,118,135,136,144,148,151,168,169,176,178] also combined self-report sleep outcomes in final analyses (i.e., reported analyses from both concurrent subjective and objective sleep data). There were seven studies [65,77,80,81,121,159,175], which collected both subjective and objective sleep data but only reported either the self-report [81] or actigraphic sleep outcomes in final analyses [65,77,80,121,159,175].

Sleep diaries or sleep logs were the most commonly used subjective sleep assessment in 47 studies (38.8%), but only 19 of these studies reported items which were from a standardised sleep diary. Modified or original sleep diary items included the Consensus Sleep Diary (CSD; *n* = 10) [9,86,110,113,120,126,129,131,148,150], the Pittsburgh Sleep Diary (PghSD; *n* = 4), [9,79,95,176], the Karolinska Sleep Diary *n* = 3) [74,78,112], and the Daily Sleep Diary (*n* = 1) [108]. Additional standardised measures included original or adapted items for daily assessment from the Pittsburgh Sleep Quality Index (PSQI; *n* = 19) [9,67,76,81,82,84,85,87,98,104,108,119,123,128,142,143,158,168,177], Insomnia Severity Index (ISI; *n* = 2) [87,132], Groningen Sleep Quality Scale (GSQS; *n* = 3) [72,115,149], Daytime Insomnia Symptom Scale (DISS; *n* = 1) [132], Daily Life Questionnaire (DLQ; *n* = 1) [71], PROMIS-SF Sleep Disturbance Scale (*n* = 1) [89], and Sleep Quality Index (*n* = 1) [161].

From the 42 studies that analysed objective sleep markers, 35 had research-grade actigraph devices, and eight [88,90,103,107,114,134,159,178] used consumer sleep trackers. Device details including model, manufacturer, wear-time, and sleep scoring algorithms, are summarised in Table 3. Research-grade devices included Actiwatch models (*n* = 22; Philips Respironics, Inc., Monroeville, PA, USA) [57,60,64,65,68,71,75,77,92,110,113,118,121,135,140,148,151,157,168,169,175,176], ActiGraph devices (*n* = 6; ActiGraph Corporation, Pensacola, FL, USA) [86,96,100,117,136,148], and Motionlogger (*n* = 5; Ambulatory Monitoring, Inc., Ardsley, NY, USA) [59,80,81,93,144]. Actigraphic and wearable data for most studies ranged from 1–2 weeks (*n* = 28) or from 3 weeks and longer (*n* = 13). Only four studies [59,65,77,168] collected actigraphic data for less than 1 week (range 3–4 days). Devices were worn on the wrist with the exception of two studies [134,159] (on the index finger) and four studies for which the wear position was not specified [64,86,103,168].

#### 3.2.5. Mood and Affect Measures

Many studies used interchangeable terms and definitions to describe affective phenomena. In this review, we broadly categorised 47 studies that monitored daily mood domains and 76 studies that reported daily affect dimensions (see Figure 3). However, there was heterogeneity across studies in defining affective phenomenon constructs. For example, da Estrela, Barker, Lantagne and Gouin [87] and Garcia, Zhang, Holt, Hardeman and Peterson [66] used the Positive and Negative Affect Schedule (PANAS) but referred to these traits as ‘mood’ outcomes. Conversely, Slavish, Sliwinski, Smyth, Almeida, Lipton, Katz and Graham-Engeland [89], Lev Ari and Shulman [63], and Chiang, Kim, Almeida, Bower, Dahl, Irwin, McCreath and Fuligni [80] used the Profile of Mood States (POMS) but described outcomes as ‘affect’ items. Therefore, for descriptive and summary purposes below, the umbrella term ‘affective experiences’ collectively refers to affect, mood, or emotion domains. Across all records, 91 studies (75.2%) assessed both positive and negative (valanced) affective states. The remaining 30 studies (24.8%) analysed positive only (*n* = 6) [64,70,84,120,123,146] or negative only (*n* = 24) [63,80,82,83,86,87,89,99,104,119,122,131,133,136,137,139,145,147,150,160,164,171,173,174] affective states. A small number of studies analysed affective arousal domains (*n* = 10) [52,74,76,77,78,82,104,112,148,168].

Standardised self-report measures were used in 68 studies (56.2%) to assess daily affective outcomes, 13 of which had adapted items from the original scales (see Table S5 for a full list of standardised affect measures). The Positive and Negative Affect Schedule (PANAS) was most widely used, with 42 studies (34.7%) incorporating either original or adapted PANAS items [9,55,57,58,59,60,65,66,67,68,70,72,75,78,79,84,85,87,90,96,98,100,101,105,108,110,113,114,119,121,127,134,139,142,146,147,148,149,160,163,167,177]. The specific PANAS subscales, the number and type of affect items (PA/NA) selected, and the rating scales varied; these included studies with items from the PANAS-C (*n* = 3) for children [59,142,177], expanded PANAS-X (*n* = 5) [67,98,113,121,148], and short-form PANAS-SF (*n* = 3) [121,127,139]. The POMS items were used in 9 studies (7.4%) [56,61,63,80,89,121,143,157,158], one of which [61] included the short-form version (POMS-SF). Twelve records (9.9%) selected items from previous ambulatory studies and/or various published scales. The remaining studies (*n* = 46; 38%) did not report the use of standardised questionnaires; daily affective experiences were assessed using author-selected items or adjectives rated on Likert scales, visual analogue scales (VAS), and sliding scales.

#### 3.2.6. Self-Report Data-Collection Format

A detailed breakdown of the number and timing of daily assessments and the self-reporting tools used across studies is outlined in Table S6. In total, 44 studies collected self-reported sleep and affect ratings concurrently. Most studies (*n* = 67) had at least one mood-affect rating that was separated in timing from subjective sleep reports, while 17 studies assessed only objective sleep data. The context of daily self-reports was rarely reported, such as location (e.g., subjective ratings, which were recorded in the home environment or workplace setting). Temporal factors (e.g., time of day) on sleep-affect assessments, however, were often reported.

Most records (*n* = 101; 83.5%) integrated at least one electronic device or digital technology for self-reported sleep and affective states. The remaining studies (*n* = 21) used paper and pencil methods [53,54,55,56,57,60,62,64,65,72,75,77,78,80,84,100,105,111,135], with three of these studies also combining online surveys [84,135], electronic tablets [145], or telephone interviews [64,145].

Digital self-report tools and sampling methods varied; 44 studies (36.4%) used online surveys, email links, webpages, and online platforms. Smartphones and mobile devices were used across 50 studies (41.3%), with 5 incorporating SMS text-based reporting and 26 using a specialised smartphone app. The ESM or EMA smartphone apps differed across studies with both Android and iOS-based platforms utilised; apps included MetricWire [90,95,113,126,148,151], movisensXS [76,149,167], the Purple Robot Android app [94], Intern+ [107], mEMA [96], MyExperience [73], RealLife Exp [118,158], Beiwe [178], Z4IP EMA [159], three customised or in-house study apps [70,170,175], and six studies, which did not report the specific smartphone app [71,74,92,128,134,141]. A small number of studies used telephone interviews and phone-call reporting (*n* = 13; 10.7%) [59,61,64,85,87,101,102,109,116,124,145,147,172] or another type of electronic device such as a small pocket computer, personal digital assistant (PDA), or tablet (*n* = 7; 5.8%) [52,79,92,121,133,143,145].

Self-reporting adherence approaches varied (see Table S6) but mostly used signal-contingent responses such as smartphone push notifications, signal alarm prompts, automated call systems, auditory buzz or ‘beepers’ (e.g., from a digital wristwatch), survey reminders, and/or prompts sent via SMS text, email, or phone call. Participant-initiated, event-based, and time-contingent responses (i.e., surveys completed at fixed times each day) were also incorporated across studies. A small number of records failed to report the signalling method or prompt design.

**Table 3:** Actigraph or wearable devices.

Author (Date)	Actigraphy Device (Manufacturer)	Concurrent Subjective Sleep Data	Device-Grade	Actigraphy Duration (Wear-Time)	Device Placement	Scoring Algorithms and Sensor Details (Provided by the Authors)
Chiang et al. (2017) [80]	Micro Motionlogger Sleep Watch (Ambulatory Monitoring, Inc., Ardsley, NY, USA)	Yes (only analysed objectively measured sleep outcomes)	Research-grade	8 days 29% (8 days), 33% (7 days), 23% (6 days), 4% (5 days), 10% (4 or fewer days)	Non-dominant wrist	Sadeh algorithm to score 60-s epochs. Action4 software (Ambulatory Monitoring, Inc., Ardsley, NY, USA) Push the event button to indicate lights off and time in or out of bed
Cousins et al. (2011) [59]	Octagonal Basic Motionlogger Wrist Actigraph (Ambulatory Monitoring, Inc., Ardsley, NY, USA)	No	Research-grade	4 days 4 nights on average during baseline and 3 nights during Week 1 (range 2–4)	Non-dominant wrist	Cole–Kripke procedure. Actigraphy data were pre-processed and scored in 60-s epochs using ActionW2.5 software Push the event button to indicate time in and out of bed
Cox et al. (2018) [86]	ActiGraph wGT3X-BT (ActiGraph Corporation, Pensacola, FL, USA)	Yes	Research-grade	7 days	Wear position not specified	Sadeh algorithm
Das-Friebel et al. (2020) [96]	ActiGraph wGT3X-BT (ActiGraph Corporation, Pensacola, FL, USA)	Yes	Research-grade	14 days	Non-dominant wrist	Sadeh algorithm to score 60-s epochs from a 3-axis MEM accelerometer. ActiLife 6 software (ActiGraph Corporation)
Difrancesco et al. (2021) [106]	GENEActiv (Activinsights Ltd., Kimbolton, UK)	Yes	Research-grade	14 days 90% with complete data over 14 days	Non-dominant wrist	Heuristic algorithm (HDCZA). Raw actigraphy data (30 Hz sample) were analysed using an open-source R package (GGIR)
Doane and Thurston (2014) [65]	Actiwatch Score (Philips Respironics, Inc., Murrysville, PA, USA)	Yes (self-reported bed-wake times are used to validate actigraphy, but only actigraphy data are reported)	Research-grade	4 days	Non-dominant wrist	Actiware-Sleep software (version 3.4) algorithm to score 60-s epochs Push the event button to indicate time in and out of bed
Fang et al. (2021) [107]	Fitbit Charge 2 (Fitbit Inc., San Francisco, CA, USA)	No	Consumer device	7 days (minimum) of raw Fitbit data for analyses. The overall study was a 1-year physician internship	Wrist-worn	R package mice and proprietary algorithms to quantify sleep from accelerometers and photoplethysmography sensors
Flueckiger et al. (2017) [81]	Motionlogger Actigraph (Ambulatory Monitoring, Inc., Ardsley, NY, USA)	Yes (only analysed self-reported sleep and affect outcomes)	Research-grade	7 days (sub-sample from study 2). 5 days (minimum) included for analyses	Non-dominant wrist	Action4 software (Ambulatory Monitoring, Inc., Ardsley, NY, USA) to score 60-s epochs
George et al. (2019) [90]	Jawbone UP3 (JB3; Jawbone, San Francisco, CA, USA)	Yes	Consumer device	10 days 6.5 days (average) and 75% completion across 10 days	Wrist-worn	A proprietary algorithm combines data from the device’s heart rate sensor and accelerometer
Gershon et al. (2012) [60]	Mini Mitter AW64 Actiwatch (Philips Respironics, Inc., Monroeville, PA, USA)	Yes	Research-grade	56 days 54 days (average)	Non-dominant wrist	Respironics Actiware Version 5.5 (Respironics, Inc.) to score 60-s epochs
Kalmbach et al. (2018) [88]	Fitbit One (2014–2015 cohort) Fitbit Charge (2015–2016 cohort) (Fitbit Inc., San Francisco, CA, USA)	No	Consumer device	2 months (before medical residency internship) and 6 months (during internship) Compliance before internship for sleep (80.2%) and physical activity (93.7%). During the internship sleep (53%), and physical activity (79.9%)	Wrist-worn	Proprietary algorithm
Li et al. (2015) [71]	Actiwatch-64 (Philips Respironics, Inc., Monroeville, PA, USA)	Yes	Research-grade	42 days 91.8% compliance (501 nights of recorded data in total)	Non-dominant wrist	Stata MP 11.2 for all statistical analyses, but scoring information is not provided Push the event button to indicate time in and out of bed
McCrae et al. (2008) [57]	Actiwatch-L (Philips Respironics, Inc., Monroeville, PA, USA)	Yes	Research-grade	14 days	Non-dominant wrist	Actiware-Sleep v. 3.3 validated algorithm set at medium sensitivity (threshold 40 activity counts) Sensor details—Actiwatch-L monitors ambient light exposure and gross motor activity. Contains an omni-directional, piezoelectric accelerometer
McCrae et al. (2016) [75]	Actiwatch-L (Philips Respironics, Inc., Monroeville, PA, USA)	Yes	Research-grade	7 days	Non-dominant wrist	Scoring information is not provided Sensor details—Actiwatch-L monitors ambient light exposure and gross motor activity. Contains an omni-directional, piezoelectric accelerometer
Merikangas et al. (2019) [92]	Respironics Monitor and Actiwatch Score (Philips Respironics, Inc., Monroeville, PA, USA)	No	Research-grade	14 days	Non-dominant wrist	Minute-by-minute activity counts. Wake and sleep periods are defined by a default threshold of <40 counts per period for more than 10 min
Messman et al. (2021) [110]	Z-machine EEG ambulatory device (General Sleep, Inc., USA) Actiwatch Spectrum (Philips Respironics, Inc., Monroeville, PA, USA)	Yes	Research-grade	7 days 524 usable days of actigraphy (94% compliance) and 476 usable days of EEG data (85% compliance)	Wrist-worn	The Z-machine algorithm scored wake periods, light sleep (stages N1 and N2), deep or slow wave sleep (stage N3), and REM sleep. A Z-machine device processes a single-channel of EEG data from 2 mastoid-placed electrodes and 1 neck-placed ground electrode Respironics Actiware version 6.0 to score actigraphy data using validated scoring hierarchy (combination of event markers, sleep diaries, light levels, and activity levels). Actiware thresholds: low threshold (activity count 10), 20 epochs of inactivity for sleep onset, and offset
Mousavi et al. (2022) [134]	Oura Ring (Ōura Health Ltd., Oulu, Finland)	No	Consumer device	3 months 43.49 (average days/person) 1623 nights of usable data	Index finger	Oura Ring algorithm from sensors (2 infrared light-emitting diode heart rate sensors, 2 negative thermal coefficient body temperature sensors, a 3-axis accelerometer, and a gyroscope)
Newman et al. (2022) [135]	Actiwatch-2 (Philips Respironics, Inc., Monroeville, PA, USA)	Yes	Research-grade	21 days (recorded as 7 consecutive days at three study time points)	Non-dominant wrist	Validated Minimitter software algorithms to estimate sleep parameters (Actiware Version)
Ong et al. (2013) [64]	Mini Mitter Actiwatch-64 (Philips Respironics, Inc., Monroeville, PA, USA)	Yes	Research-grade	7 days	Wear position not specified	Scoring information is not provided
Parsey and Schmitter-Edgecombe (2019) [93]	Mini Motionlogger (Ambulatory Monitoring, Inc., Ardsley, NY, USA)	No (only recorded self-reported sleepiness/fatigue levels)	Research-grade	7 days	Non-dominant wrist	University of California, San Diego, sleep scoring algorithm at 60-s epochs
Patapoff et al. (2022) [136]	ActiGraph Actisleep-BT (ActiGraph Corporation, Pensacola, FL, USA)	Yes	Research-grade	14 days Sample with both actigraphy and EMA data for 2–11 days. Actigraph was worn for 14-day periods in annual assessments. Some had data from multiple (2–4) years	Wrist-worn	Cole-Kripke algorithms
Ryuno et al. (2021) [100]	ActiGraph GT9X (ActiGraph Corporation, Pensacola, FL, USA)	No	Research-grade	14–56 days 29.1 (average period)	Non-dominant wrist	ActiLife software (version 6, ActiGraph Corporation)
Shen et al. (2022) [113]	Mini Mitter Actiwatches (–L, −64, −2, Spectrum, Pro, and Plus; Philips Respironics, Inc., Monroeville, PA, USA)	Yes	Research-grade	28 days 24.4 (average) with 4868 nights of usable data	Wrist-worn	Data were collected at 60-s epochs and analysed on a medium threshold in Actiware 6.0.9
Shi et al. (2021) [114]	Mi Band (model not specified) (Xiaomi Inc., Beijing, China)	No	Consumer device	7 days	Wrist-worn	Scoring information is not provided
Sun-Suslow et al. (2021) [117]	ActiGraph GT9X Link device (ActiGraph Corporation, Pensacola, FL, USA)	Yes	Research-grade	14 days	Non-dominant wrist	Scoring information is not provided
Takano et al. (2014) [68]	Actiwatch Mini Mitter (Philips Respironics, Inc., Monroeville, PA, USA)	No	Research-grade	8 days 6.3 (average nights/person)	Non-dominant wrist	A software algorithm scored in 60-s epochs Push the event button to indicate time in and out of bed
Tavernier et al. (2016) [77]	Actiwatch Score (Philips Respironics, Inc., Monroeville, PA, USA)	Yes (only analysed objectively measured sleep outcomes)	Research-grade	3 days	Non-dominant wrist	Actiware-Sleep software (version 3.4, MiniMitter/Philips Respironics)-validated algorithm used to score sleep data in 60-s epochs
Titone et al. (2022) [140]	Actiwatches (Philips Respironics, Inc., Monroeville, PA, USA)	No	Research-grade	20 days 73% compliance and 1562 observations	Non-dominant wrist	Scoring information is not provided
Tzischinsky et al. (2001) [54]	Mini-Act (Ambulatory Monitoring, Inc., Ardsley, NY, USA)	No	Research-grade	5–7 days	Non-dominant wrist	Sleep data were scored in 60-s epochs
Vigoureux and Lee (2021) [118]	Actiwatch Spectrum Plus (Philips Respironics, Inc., Monroeville, PA, USA)	Yes	Research-grade	14 days	Wrist-worn	Philips-Respironics v6.09 software validated algorithms
Wen et al. (2020) [103]	Sports bracelet (model not specified)	No	Consumer device	7 days	Wear position not specified	Scoring information is not provided
Wong et al. (2021) [121]	Actiwatch-16 (Philips Respironics, Inc., Monroeville, PA, USA)	Yes (sleep log to indicate wake-times on EMA days)	Research-grade	7 days (4 of these were EMA self-report days)	Wrist-worn	Actiware software (v5.59) automated, standard medium thresholds. Data scored in 60-s epochs
Zohar et al. (2005) [55]	Mini-Act-32 (Ambulatory Monitoring, Inc., Ardsley, NY, USA)	Yes	Research-grade	5–7 days This study surrounded nightshifts every 6 months, covering the first 2 years of medical residency	Non-dominant wrist	Actigraphic scoring analysis software program for 60-s epochs
Kouros et al. (2022) [144]	Octagonal Basic Motionlogger Wrist Actigraph (Ambulatory Monitoring, Inc., Ardsley, NY, USA)	Yes	Research-grade	7 days 5.96 nights (average); 86.5% had at least 5 nights of actigraphy; 44.7% had valid actigraphy for all 7 nights	Non-dominant wrist	Sleep data were scored in 60-s epochs using a zero-crossing mode. Raw data were analysed with ACTme software (Action W2, 2002, Ambulatory Monitoring, Inc., Ardsley, NY, USA) using Sadeh’s algorithm An event marker is pressed to indicate first attempt to fall asleep
Chachos et al. (2023) [148]	The STEPS study used Mini Mitter Actiwatches (Actiwatch Spectrum Pro, Spectrum Plus, Actiwatch-2, Actiwatch-64, and Actiwatch-L; Philips Respironics, Inc., Monroeville, PA, USA) ACES and DESTRESS studies used ActiGraph wGT3X-BT (ActiGraph Corporation, Pensacola, FL, USA)	Yes	Research-grade	7–28 days STEPS (28 days), ACES (12 days), and DESTRESS (7 days)	Wrist-worn	STEPS Mini Mitter Actiwatches: scored using combined information from the sleep diary, ambient light (when available), and activity patterns scored with 60-s epochs and a “medium” threshold for sleep-wake detection in Actiware (v. 5.5) ACES and DESTRESS ActiGraph wGT3X-BT: ActiLife software (v.6.13.3) with the Cole-Kripke algorithm
Kirshenbaum et al. (2023) [151]	Actiwatch Spectrum Plus (Philips Respironics, Inc., Monroeville, PA, USA)	Yes	Research-grade	3–14 days At least 3 days of usable actigraphy data	Wrist-worn	Event marker for sleep onset and time out of bed upon waking
Master et al. (2023) [157]	Actiwatch Spectrum (Philips Respironics, Inc., Monroeville, PA, USA)	No	Research-grade	3–7 days At least 3 valid actigraphy days. With an average of 5.4 ± 1.5 days of valid sleep actigraphy aligned with next-day mood (range 3–9 days), 69% (n = 363) had at least 5 days of valid sleep actigraphy with next-day mood data	Non-dominant wrist	Philips Actiware software (Version 6.0.4, Philips Respironics, 2017) downloaded sleep data at the 30-s epoch level
Ng et al. (2023) [159]	Oura Ring (Ōura Health Ltd., Oulu, Finland)	Yes (self-reported bed-wake times are only used to check Oura sleep periods)	Consumer device	28 days (Study 1) and 2 weeks (Study 2)	Finger (non-dominant hand)	Sleep-wake periods are estimated by Oura’s proprietary algorithm that uses body movement, heart rate variability, a circadian factor, and temperature
Poon et al. (2024) [175]	Actiwatch Spectrum Plus (Philips Respironics, Inc., Monroeville, PA, USA)	Yes (self-reported bed-wake times and event marker used to adjust actigraphy)	Research-grade	7 days	Non-dominant wrist	Actiware version 6.0.9 (Philips Respironics, Inc.). Actigraphy devices aggregated in 60-s epochs with a sample rate of 32 Hz. Event markers and self-reported bed-wake times were used to adjust the actigraphy
Wescott et al. (2024) [176]	Actiwatch Spectrum (Philips Respironics, Inc., Monroeville, PA, USA)	Yes	Research-grade	3–17 days	Non-dominant wrist	Data sampled in 30-s epochs with event markers to indicate sleep initiation
Zapalac et al. (2024) [178]	Fitbit Inspire HR (Fitbit Inc., San Francisco, CA, USA)	Yes	Consumer device	35 days Average observation days (35.20; SD = 27.01)	Wrist-worn	Proprietary Fitbit algorithms converted accelerometer and heart rate data into sleep and physical activity measures
Dong et al. (2024) [169]	Actiwatch-2 (Philips Respironics, Inc., Monroeville, PA, USA)	Yes	Research-grade	3–9 days Minimum 3 days with a sleep diary. Average wear-time 7.15 days (SD = 1.07), range (3–9)	Wrist-worn	Validated scoring algorithms with bed-wake times to set intervals
Collier Villaume et al. (2024) [168]	Mini Mitter Actiwatch-64 (Philips Respironics, Inc., Monroeville, PA, USA)	Yes	Research-grade	3 days	Not specified	Within-person (day-to-day) analyses utilised continuous measures of daily TST and TIB. Categorised ‘short’ and ‘not short’ (regular) sleepers

*Note.* Device trademark rights may have changed since the original publication. For example, Philips Respironics, Inc. (Pennsylvania, USA) acquired Mini-Mitter and Actiwatch-L. **EEG** = Electroencephalogram.

### 3.3. Affective Disorders

#### 3.3.1. Bipolar Disorder

Seven studies included samples with bipolar-spectrum disorders. Four records found significant associations between self-reported sleep disturbances and next-day mood or affect [60,61,136,141] and two for objective sleep parameters. Sleep duration was associated with better daily mood symptoms [141], poorer SQ predicted higher next-day negative mood (sadness) [136], and lower SE was linked with higher NA [60]. Sleep disturbances (WASO) were associated with next-day negative affect [60] and mood [61], while total wake-time (SOL + WASO) predicted the next morning negative mood [61]. Only one study [91] did not find a significant impact of self-reported TST on mood levels (positive or negative) the next day. Four studies captured objective sleep data (actigraphy); longer TST was associated with fewer next-day depressive symptoms [140], and longer SOL was linked to higher negative affect [60]. One study did not find an association with objective sleep markers (TST) on next-day mood symptoms (positive or negative) in bipolar or unipolar depression [92].

Across studies with bipolar disorder, five also assessed the impact of mood or affective experiences on subsequent sleep. Three found worse mood or negative affect impacted subsequent sleep disturbance (WASO, SOL, TWT) [60,61,136], SE, and sleep onset time [136]. Two studies, however, had mixed or unexpected findings, with Merikangas, Swendsen, Hickie, Cui, Shou, Merikangas, Zhang, Lamers, Crainiceanu, Volkow and Zipunnikov [92] reporting no association between mood levels (positive or negative) and TST the next day, while Li, Mukherjee, Krishnamurthy, Millett, Ryan, Zhang, Saunders and Wang [91] found elevated mood symptoms were associated with reduced TST the next night.

#### 3.3.2. Anxiety and Depressive Disorders

Only four studies included participants with diagnosed anxiety and/or comorbid depressive disorder among children or adolescents [59,173] and adult [106,165] samples. Better self-reported SQ predicted elevated positive and lower negative affect (especially among individuals diagnosed with depression or anxiety), but there was no association with TST [106]. Better SQ but not longer TST was predictive of daily affect levels (higher positive affect and lower negative affect) [165]. Self-reported TST or SQ, however, did not impact subsequent affect variability (positive or negative) [165].

There was varied evidence for actigraphic sleep indices, which depended on the affect outcome and clinical group. Cousins, Whalen, Dahl, Forbes, Olino, Ryan and Silk [59], for example, found longer sleep duration (actigraphic TST) was associated with improved next-day positive affect for adolescents diagnosed with an affective disorder (depression and anxiety) but not healthy controls, while Difrancesco, Penninx, Antypa, van Hemert, Riese and Lamers [106] did not find actigraphy-based TST predictive of subsequent same day affect. Reduced sleep latency (actigraphic-SOL) was linked to lower next-day negative affect and higher positive affect for depressed adolescents [59], while SE scores did not impact next-day affect [59].

Studies with anxiety disorder and/or depressed participants (*n* = 4) reported mixed bidirectional relationships. Daytime negative affect was related to less actigraphy-based wake time (WASO) in depressed adolescents [59], whereas lower negative affect the preceding day predicted better self-reported SQ, with effects strongest for depressed and anxious individuals [106]. Negative mood rated by children and adolescents in the evening did not predict lower nightly self-reported TST [173], but decreased TST, conversely, was linked to increased negative mood (morning irritability). Higher negative affect variability was associated with worse SQ but not TST, while higher daily levels of negative affect did not significantly predict subsequent TST or SQ [165]. Positive daytime affect also had mixed patterns; more positive affect was linked to longer time in bed that night (actigraphic TIB) and total sleep (actigraphic TST) for depressed adolescents, but conversely, less time in bed (actigraphic TIB) for adolescents with anxiety only [59]. Meanwhile, higher daily levels of positive affect (in adults with anxiety disorders and/or depression along with controls) were associated with better SQ but not TST [165]. Positive affect variability, however, was not significantly related to subsequent sleep indices (TST and SQ) [165].

Seven other studies included individuals with major depression or depressive disorders. Improved self-reported SQ predicted next-day positive affect [79], better mood [83], and lower negative affect [79]. Poorer SQ was also associated with lower morning positive affect [111,176], especially for individuals with greater depression severity [176]. Sleep duration (TST) was also non-linearly associated with daily affect (positive and negative) across depressed and non-depressed individuals [172]. Sleep the previous night (lack of hours slept and excessive sleep) was associated with lower positive and higher negative affect [172]. Disturbed sleep (delayed SOL) the previous night also dampened mood scores [83]. Daytime affect or mood levels (positive or negative) were not associated with subsequent sleep (SQ, TST) across two studies with major depression or depressive disorders [79,92]. For actigraphic sleep parameters, Poon, Cheng, Wong, Tam, Chung, Yeung and Ho [175] found no significant relationships between mood (positive or negative) and objective sleep indices (TST, SE, SOL, TIB, and WASO). Wescott, Taylor, Klevens, Franzen and Roecklein [176], meanwhile, found that longer sleep than usual (actigraphic TST) was related to better morning mood, while variability in nightly sleep duration was more impactful on mood or affect for depressed individuals compared to controls.

### 3.4. Shift Workers

Eight records investigated shift workers. Sleep loss (shorter self-reported and actigraphic TST) among medical residents impacted emotional reactions to affective work events (positive and negative) [55]. Poor sleep (shorter TST) also amplified negative emotions and dampened positive emotional responses to daytime events [55], and shorter sleep (TST) predicted a worse mood the next day, which also led to shorter sleep the following night [88]. Nurses with poorer sleep quality (SQ; within-person) had reduced daily positive affect and a higher daily negative affect [118], while in another nurse sample, worse sleep (TST via indirect effects of SE) was linked to higher depressed mood the following day [150]. Shift workers across a range of retail and service sectors (with nonstandard hours) had a more positive mood after higher amounts of self-reported TST [162]. For studies with actigraphy, TST was associated with positive affect and mood levels in healthcare workers [55,107,118] but SE [118] had no impact. Medical residents with later wake times, earlier bedtimes, and fewer shifts in total sleep (TST) also reported improved next-day mood [107]. One study with medical residents had mixed sleep-affect patterns [54]: at the start of medical residency, sleep loss during a night shift was associated with elevated negative but not positive mood the following day. However, after 6 months of medical residency, sleep disruption (lower TST) after a night shift did not impact the next-day mood (positive or negative) [54]. No studies with shift workers reported outcomes for SOL or WASO.

Only two shift work studies reported mood or affect outcomes that temporally preceded sleep. Lower mood predicted worse TST the next-day [88], and greater positive affect was linked to improved SQ [98].

### 3.5. Sleep on the Next Day Mood or Affect

**Figure 4:**
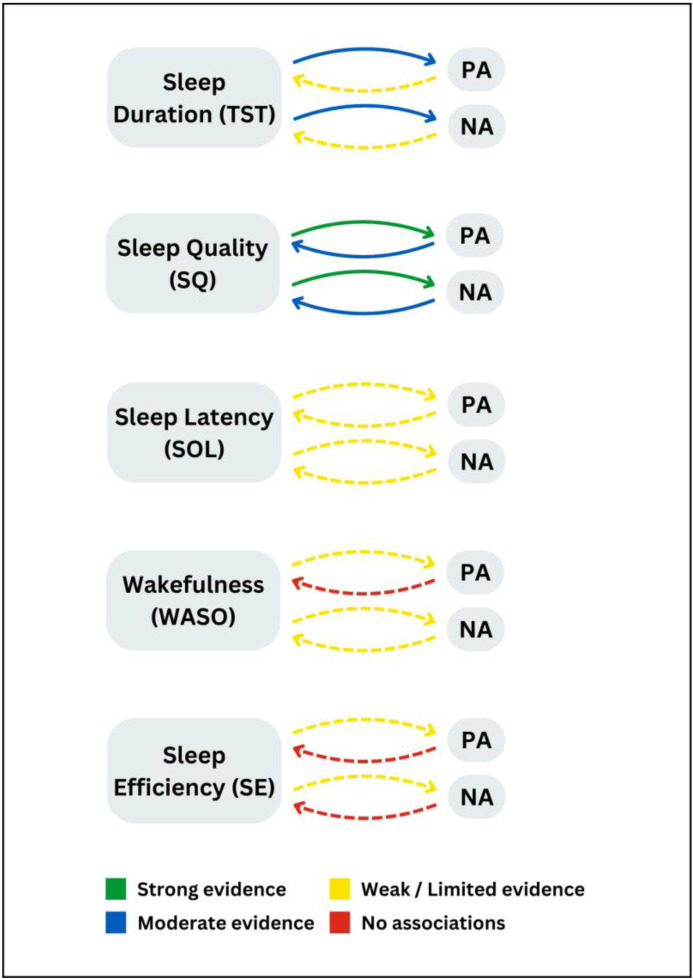
Bidirectional relationships between sleep indices (subjective and objective) and positive (PA) and negative (NA) affective experiences. Evidence from studies with healthy populations (*n* = 97) was rated based on reported findings as strong (green solid), moderate (blue solid), weak or limited (yellow dotted), or no associations (red dotted). Associations refer to (significant) expected directions.

The remaining studies had healthy populations (*n* = 97) with evidence for bidirectional relationships across five sleep indices (TST, SQ, SOL, WASO, and SE) and affective experiences (positive and negative) represented in Figure 4. Given the multitude of sleep-affect relationships, evidence for both objective and subjective sleep parameters is pooled in Figure 4.

#### 3.5.1. Sleep Duration

Self-reported TST in general was significantly related to next-day positive affect or mood in 13 of 25 studies, including children or adolescents [56,90,177] and adult samples [62,85,102,109,110,116,117,120,129,135,149,163]. Less TST and worse sleep were related to lower positive daily moods [56,116,117], while longer and more consistent patterns of self-reported TST in general were significantly related to higher next-day positive affective states [62,85,90,102,110,120,135,149,163,177].

Two records also reported curvilinear effects of overall sleep loss for both positive and negative affect the following day [101,109], and one study [158] found an inverse relationship, such that longer sleep duration was linked to a lower next-day positive mood (reduced happiness). Seven studies had null results of self-reported sleep duration and next-day positive affective experiences [66,74,96,124,126,144,148], of which four included children or adolescents [66,74,144,148]. Only 3 of 14 studies found an impact of longer objective sleep duration (actigraphic-TST) on better subsequent positive affective experiences [135,159,168]. Nine records [57,65,90,96,103,121,144,148,157] did not demonstrate improved positive affect following longer actigraphic-recorded sleep duration; one study found inconsistent associations with composite mood compared to individual mood items [71], and one study reported an inverse relationship, such that increased sleep (actigraphic-TST) was linked to lower positive affect the next day [134].

Self-reported TST in general was associated with next-day negative affect in 17 of 32 studies [52,56,58,86,99,102,105,113,116,117,122,128,135,144,147,164,177], 7 of which were with adolescents or young adult samples [56,58,99,105,113,144,177]. Shorter self-reported night-time TST was related to worse next-day negative affect, lower mood, or emotions [52,56,58,86,105,116,117,122,128,147,164,177]. Longer self-reported TST was associated with next-day lower negative mood, affect, or emotions [99,102,113,135,144].

Ten studies did not find a direct association between the amount of subjectively reported TST and next-day negative affective experiences among adults and children or adolescents [63,66,67,74,96,124,126,148,149,163]. Three studies also had varied findings: Kalmbach, Arnedt, Swanson, Rapier and Ciesla [82] found that one night of short sleep (TST) led to reduced next-day anhedonic depressive symptoms, but shorter sleep (TST) across a longer 2-week period was associated with higher anhedonia. Bean and Ciesla [104] reported increased anxious arousal symptoms following partial sleep deprivation (TST), but next-day anhedonic depressive and general distress were not impacted. Shorter sleep (TST) among adolescents [69] worsened next-day affective well-being, but among adults (over 20 years old), both shorter or longer sleep duration impacted affect the following day, thus highlighting a non-linear relationship.

There was varied evidence for actigraphic-recorded TST on negative mood. Sleep duration (actigraphic-TST) predicted next-day negative mood symptoms or affect ratings in seven studies [86,100,117,135,157,168,169], but there were no reported associations in eight other studies [57,65,96,103,114,121,144,148]. One record also had inconsistent findings; actigraphic sleep (TST) was not associated with composite mood items but with 2 of 11 individual mood items [71].

#### 3.5.2. Sleep Quality

Overall, 33 studies reported a significant impact of subjective SQ and sleep satisfaction on positive mood or affective dimensions the following day [52,53,57,58,62,64,67,70,71,72,73,74,76,78,81,84,85,90,94,95,96,101,112,117,120,125,126,130,135,138,142,146,149]. Associations were found across children and adolescents [58,73,74,90,112,142], older adults [57], and adults. Only 7 studies did not find an association between self-reported sleep quality and next-day positive affective experiences [66,75,123,124,132,144,151], including three with children or adolescents [66,144,151].

Subjective nightly SQ predicted negative affect and mood ratings the next day in 38 of 42 studies among children or adolescents [58,73,74,99,112,137,142,151,169,170], older adults [57,145], and adults [62,71,72,78,81,82,94,95,96,115,117,119,122,125,126,130,131,132,133,135,138,139,149,158,164,174]. Five out of the identified studies did not find a significant relationship between sleep quality and negative affect among adolescents or adults [66,67,101,124,177].

#### 3.5.3. Sleep Latency and Wakefulness after Sleep Onset

There was limited evidence for the impact of self-reported SOL and the frequency of nocturnal WASO on affective states. Shorter self-reported SOL was related to higher next-day positive affect and mood in 3 of 5 identified studies [52,62,110] and shorter WASO was associated with more positive next-day affect in 2 studies [52,120]. Two studies did not find a relationship between subjective sleep disturbance (SOL) and positive mood [132,177], and one study did not find significant associations between night-time WASO and positive affective experiences among adolescents [143].

Actigraphic-recorded SOL and WASO on positive affect scores also had limited evidence. Mousavi, Lai, Simon, Rivera, Yunusova, Hu, Labbaf, Jafarlou, Dutt, Jain, Rahmani and Borelli [134] found longer sleep latency (actigraphic-SOL) the previous day was linked to lower next-day positive affect. Doane and Thurston [65] with adolescents and Parsey and Schmitter-Edgecombe [93] with adults, however, did not find a relationship between objective (actigraphic) SOL indices and next-day reports of positive mood. No studies reported an impact of actigraphic WASO on next-day positive affective experiences [57,93].

Six of seven studies found that longer subjective SOL was linked to a poorer negative mood the following day [52,62,132,167,174,177] and just one identified study by Kalmbach, Pillai, Roth and Drake [67] reported no influence on negative affect ratings (as with other reported sleep indices such as TST and SQ). Greater self-reported WASO and night-time disturbance were associated with elevated negative affect and poorer mood in six studies across adolescents and adults [75,110,143,167,174,177], and one record did not find a significant relationship [67]. Actigraphic-recorded SOL [65,93] and WASO [57,93] did not impact next-day affect or mood.

#### 3.5.4. Sleep Efficiency

Greater self-reported SE was associated with higher daily positive affect [110,120] and negative affect [87,113]. One study, however, did not find significant main effects of self-reported SE on positive affect (high and low arousal) among adolescents and young adults [148]. Takano, Sakamoto and Tanno [68] demonstrated a significant association between decreased actigraphic-measured SE and reduced positive affect levels the following day, while Master, Nahmod, Mathew, Hale, Chang and Buxton [157] found individuals who slept more efficiently than their average reported a higher next-day positive mood (happiness ratings). The remaining identified studies (*n* = 7) did not find any significant relationships between objective SE and next-day positive affect [65,93,96,113,121,135,148]. Worse objectively recorded sleep efficiency (actigraphic-SE) was associated with increased next-day negative mood in just one study [117], with remaining studies reporting inconsistent [71,135] or null findings among adults, older adults, or adolescents [65,93,96,113,121,144,148,157].

### 3.6. Daytime Mood or Affect on Subsequent Sleep

#### 3.6.1. Positive Affect

More daytime positive affect or mood states predicted longer TST in 2 out of 19 identified studies with adolescents [56] and adults [67]. One study with older adults [9] also found greater variability in daily positive affect was linked to lower sleep duration and more tiredness. Evidence from the remaining studies did not find a significant relationship between positive mood or affective experiences on subsequent TST [52,58,62,65,68,69,100,102,121,135,157,163,177] and three studies reported mixed findings [108,113,178]. Zapalac, Miller, Champagne, Schnyer and Baird [178], for example, found that positive affective states in the morning (but not evening) were related to longer actigraphic sleep duration (as well as shorter SOL, better SQ, and fewer awakenings).

Daytime positive affective experiences were generally associated with self-reported SQ the following night, including for children and adolescents [58,73,74,112,144,169,170] and adults [53,64,67,94,98]. Daytime positive affect or mood states did not significantly predict subsequent subjective SQ across 5 studies [52,72,130,135,177]. Mixed results were reported by Jones, Smyth and Graham-Engeland [108] at the between- and within-person levels and Zapalac, Miller, Champagne, Schnyer and Baird [178] found discrepancies between positive affective states recorded in the morning compared to-evening and their subsequent impact on SQ. de Wild-Hartmann, Wichers, van Bemmel, Derom, Thiery, Jacobs, van Os and Simons [62] also found a negative association such that greater daytime positive affect was associated with lower SQ the next night (i.e., better mood was unexpectedly linked to worse sleep quality).

Only 3 of 9 studies found a significant impact of daily positive affect on actigraphic [121] or subjective [67,178] SOL. The remaining identified studies (*n* = 4) did not find an association with positive affect on subsequent SOL [52,62,65,68] or nocturnal wakefulness (WASO) [52,62,177]. Tavernier, Choo, Grant and Adam [77] also reported mixed findings depending on positive affect arousal; feeling calm (low-arousal PA) predicted shorter SOL, while excitedness (high-arousal PA) predicted a longer SOL that night. Generally, low-arousal affective experiences (regardless of positive or negative valence) were related to better sleep outcomes compared to worse sleep for high-arousal daytime affective feelings [77].

Evidence for positive daytime affect impacting SE was limited. Only 1 of 8 studies reported a relationship between positive affect reactivity and subsequent SE [64], while the remaining studies did not find an association (for self-reported or actigraphic SE) [65,68,121,157]. Two studies reported inverse relationships: Messman, Slavish, Dietch, Jenkins, ten Brink and Taylor [110] found lower-than-average positive morning affect was associated with higher actigraphic and self-reported sleep efficiency that night; Kouros, Keller, Martín-Piñón and El-Sheikh [144] found that higher ratings of positive mood (happiness) were associated with lower actigraphic sleep efficiency.

#### 3.6.2. Negative Affect

Elevated daytime negative affect or poorer mood among adults and adolescents impacted shorter actigraphic [77,113] and self-reported TST [67,82,122,167,177]. The remaining studies (*n* = 16), however, did not find an association with daily negative affective experiences on subsequent sleep duration (self-reported and objective TST) [52,56,58,63,65,69,74,85,86,99,102,121,128,147,157,163]. One study [144] found an inverse relationship, such that greater negative mood during the daytime was linked to higher nightly actigraphic sleep duration and efficiency.

Evidence from 16 of 27 studies reported daytime negative mood or affect impacting subsequent self-reported SQ among children or adolescents [66,73,144,177], older adults [9,166], and adults [67,82,89,94,119,125,127,135,160,171]. Eleven studies found daily negative mood or affect symptoms were unrelated to subsequent SQ indices [52,58,62,72,74,85,99,115,122,130,145] and two studies from Neubauer, Kramer, Schmidt, Könen, Dirk and Schmiedek [112], and Zapalac, Miller, Champagne, Schnyer and Baird [178] reported inconsistent effects on subsequent nightly sleep quality.

Only 3 of 9 studies [67,82,178] reported a significant impact of daytime negative affective experiences on impaired SOL, with Zapalac, Miller, Champagne, Schnyer and Baird [178] reporting associations only between SOL and morning (not evening) affect states. The remaining studies did not report any significant relationship between daytime negative mood or affect outcomes on subsequent SOL [52,62,65,121,177]. There were only three studies that reported findings of negative affect and WASO. Tavernier, Choo, Grant, and Adam [69] reported higher negative social evaluative emotions (higher anxious-nervous) among adolescents predicted longer wake bouts that night (WASO) at the within-person level, while Totterdell, Reynolds, Parkinson and Briner [52] and Xie, Zhang, Wang, Chen and Lin [177] did not find previous day mood states predictive of subsequent nocturnal wakefulness.

No studies reported a significant association (in the expected direction) between negative affect and subsequent sleep efficiency (actigraphic or self-reported SE) [65,121,157]. One study from Kouros, Keller, Martín-Piñón and El-Sheikh [144], however, reported an inverse relationship with a higher daily negative mood linked to greater sleep efficiency that night.

## 4. Discussion

Sleep and affective states are mutually connected. Emerging evidence from studies with ambulatory monitoring has shed light on the temporal relationships and dynamic patterns of sleep disturbances and affective experiences. This systematic review provides updated evidence in a rapidly emerging field and expands on previous reviews by including bipolar disorder subtypes and shift workers. As visualised in Figure 4, patterns of sleep-affect associations are summarised for healthy, non-clinical samples involving children, adolescents, adults, and older adults.

### 4.1. Key Findings

This systematic review screened 3024 records and identified 121 studies for inclusion. Only a fifth of records (*n* = 23) combined both self-report and objective (e.g., actigraphy) sleep outcomes in analyses. Most studies incorporated a standardised sleep or affective measure, with the majority utilising digital self-report tools (*n* = 101). Common sleep measures included the Consensus Sleep Diary (CSD) and the Pittsburgh Sleep Quality Index (PSQI), and for mood or affect measures, this included the Positive and Negative Affect Schedule (PANAS) and the Profile of Mood States (POMS). Sleep parameters ubiquitously assessed were sleep duration (TST; 75.2%), sleep quality (SQ; 63.6%), sleep onset latency (SOL; 29.8%), sleep efficiency (SE; 28.1%), and time awake after sleep onset (WASO; 19.8%). Despite heterogeneity in terminology, 47 records were categorised as utilising daily mood domains, and 76 studies had affect dimensions. Most studies separated the timing of sleep and mood-affect ratings, but over a third collected concurrent ratings.

Bidirectional patterns are summarised in Figure 4 for healthy populations. Most studies focused on sleep preceding subsequent affective states. Sleep duration (TST) and sleep quality (SQ) were strongly linked to daily positive and negative affective states compared to other sleep indices. Sleep latency (SOL), nocturnal wakefulness (WASO), and sleep efficiency (SE) had inconsistent evidence; while no clear patterns emerged for these domains (SOL, WASO, and SE), there was a more limited pool of studies. Daytime affect or mood-predicting subsequent sleep also had mixed evidence. Positive or negative affective experiences had limited evidence of influencing subsequent sleep duration (TST), latency (SOL), wakefulness (WASO), and sleep efficiency (SE). Only sleep quality (SQ) had moderately strong evidence for reciprocal sleep-affect associations. Sleep and affect moderators may explain these findings, such as individual coping strategies, differences in individual emotional regulation or reactivity, cognitive or self-control, and sleep hygiene, habits, or beliefs [16,44,179,180]. Additional mediating factors were also rarely controlled for, such as the influence of age, gender, chronotype propensity, physical activity, daily variables, or bedtime procrastination (e.g., electronic device usage at night) on sleep-affect outcomes [2].

Shift working samples were less common (*n* = 8), but sleep duration (TST) was shown to impact both next-day positive and negative affect. A bidirectional sleep–mood association was shown for sleep quality but was reported by only one identified shift work study, which limits generalisability [88]. Better self-reported sleep (SQ, SOL, SE, and WASO), in general, predicted mood or affect scores the following day in samples with a diagnosed affective disorder. Mixed or varied patterns were observed for daily self-reported and actigraphic-recorded sleep duration (TST). Reciprocal sleep-affect associations were also varied. In general, daytime negative mood predicted poorer subsequent sleep in clinical samples of affective disorders. Only a third of studies with an affective disorder, however, reported the impact of daytime positive mood or affect outcomes, and from these records, there was weak or no evidence for a direct association. Generalisability of findings for affective disorders is also limited by age, as only two studies [59,173] assessed non-adult populations.

### 4.2. Limitations

Methodological variance and heterogeneity in operational definitions (e.g., for affective outcomes) were limitations in the present review. Studies used interchangeable terms to describe mood, emotions, or affect. These are broad but often conflated domains that can differentially impact sleep [181]. In this review, a range of affective phenomena were considered to evaluate a wider pool of available evidence and to consolidate the significant heterogeneity across ambulatory sleep-affect studies. Future research, however, should conceptually clarify and disentangle these complex interactions between sleep and multifaceted affect states. Ambulatory assessment is also an umbrella term [182,183,184] used to capture daily sampling but includes a range of methods. Variability in sleep or actigraphic parameters analysed, single or multi-item measures, sampling resolution, scheduling and prompting, sleep monitoring periods, and response compliance may also have impacted findings. Records that fail to incorporate a standardised affective measure may limit the replicability of findings, given that item composition and operationalisation (e.g., wording) differ markedly across studies [41]. Contextual parameters and situational drivers were largely overlooked across studies, which have been shown to impact mood symptoms that fluctuate over time [185].

Future ambulatory studies would benefit from adopting additional, objective physiological markers to assess sleep-affect relationships [186]. Just over a third of studies identified in this review utilised objective wearables (Figure 3 and Table 3), but very few captured additional multidimensional sleep-circadian biomarkers (e.g., temperature, cardio-respiratory function, general psychomotor levels, or light data) [187]. Wearable device advancements have enabled researchers to more accurately monitor real-time sleep and diurnal activity outside of laboratory settings [188,189]. Multiple sensors integrated within new-generation wearables, such as electrodermal activity (EDA), can be used to estimate sleep stages, detect sleep disorders, or indicate sleep quality and emotion classification [187,190,191,192].

### 4.3. Research Agenda

This systematic review highlighted current gaps in the literature and future study recommendations:

1. Standardised affective measures should be utilised in future studies to afford consistency in reporting, robust quantification, and specificity of findings. Future research should consider both positive and negative valence, affective arousal domains, and specific emotions, which may be differentially impacted by sleep.
2. Multi-modal sleep assessments should encompass both subjective (standardised self-report measures) and objective (e.g., actigraphic) sleep parameters across a range of sleep domains: these include sleep duration (TST), sleep quality (SQ), sleep latency (SOL), sleep efficiency (SE), wakefulness (WASO), time in bed (TIB), and sleep timing variability. The inclusion of multiple sleep features enables unique and granular predictions of affective function interrelationships.
3. The optimal study length should be at least 7 to 14 days to capture sleep-affect interplay. This allows for daily function variability, captures working and non-working days (in line with ICSD-3 recommendations), and identifies acute, cumulative, or cascading effects.
4. There is a paucity of studies to date utilising ambulatory tools to assess the mutual interplay of daily sleep and mood among affective disorders and shift workers; two groups vulnerable to disruptions in circadian and sleep pattern rhythmicity.
5. To avoid contextual bias, future studies should consider the timing and sampling resolution of daily assessments and situational drivers. Time of day effects (e.g., mood-congruent biases due to proximity to sleep-wake intervals) and frequency of affect assessment (e.g., multiple or single ratings) should be considered. Multiple daily measures, in particular, are needed to capture both transient mood changes and affect states.

## 5. Conclusions

Reciprocal sleep-affect associations were complex and evidenced across affective disorders (bipolar, depression, and anxiety), shift workers, and non-clinical populations. Overall, the pattern of findings indicates sleep disturbances, particularly poorer sleep quality and shortened sleep duration, were related to decreased daytime positive and increased negative affective experiences. Sleep was a stronger predictor of subsequent mood and affect, rather than vice versa. The strength and magnitude of sleep-affect connections were more robust for self-reported (subjective) sleep markers compared to actigraphic (objective) sleep markers. Future research is needed to further elucidate the impact of daytime affect (especially positive moods) on subsequent sleep.

## Figures and Tables

**Table 1:** Inclusion and exclusion criteria.

Category	Inclusion Criteria	Exclusion Criteria
Study design	Naturalistic study design, which must include an ambulatory technique or prospective daily measure of sleep (nightly) and affective experiences (mood, affect, or emotions)	No inclusion of daily outcome variables: sleep or affective experiences (mood, affect, or emotions)
Outcomes/study design	Daily assessment of sleep parameters (objective or subjective) and affective experiences (positive or negative) over multiple days (≥2 days). Outcomes must be reported by individuals directly (within-subject) and not secondary (e.g., parental reported outcomes were not included)	The following study designs and types were not eligible: Cross-sectional/single-point studies Animal Single-case/single-group studies Laboratory-based/experimental paradigms Forced desynchrony protocols Intervention studies (e.g., behavioural/pharmacological treatment of sleep or affect) or individuals receiving sleep-inducing medications (e.g., hypnotics, sedatives, and melatonin) Studies investigating the following areas were not eligible: Jet lag Sleep deprivation or restriction Napping Nightmares Substance dependence/illicit substance or alcohol use
Analysis reported	Direct analysis of the relationship (unidirectional or bidirectional) between daily sleep and daily affective experiences (mood, affect, or emotions)	Analysis focused solely on covariates or mediating factors was excluded
Publication type/date	Selected articles were empirical studies published in peer-reviewed journals in English. All publications until 28 May 2024 were included	Non-empirical or secondary research (e.g., meta-analyses, review papers, conference proceedings, case reports, or case series) or qualitative studies
Participants	Studies with clinical or healthy samples were considered across all age groups. ‘Clinical’ populations were limited to affective disorder diagnoses including bipolar subtypes (BD I, BD II, or BD-NOS), depressive disorders, or anxiety disorder. The diagnosis (DSM/ICD criteria) of affective disorder must be confirmed through validated instruments or a clinical interview. The illness phase (acute or nonacute) was recorded (e.g., illness episode or populations experiencing remission or interepisode status)	Studies in the following populations were not eligible: Diagnosed sleep disorder (e.g., insomnia, obstructive sleep apnoea), excluding individuals with a comorbid affective disorder (e.g., insomnia and bipolar) Shift work disorders Medical or psychiatric condition other than an affective disorder (bipolar, depression, or anxiety)—apart from studies with an adequate control group or comparator healthy sample with no psychiatric or sleep disorder

**BD I and BD II** = Bipolar Disorders Type I and Type II; **BD-NOS** = Bipolar Disorder-Not Otherwise Specified; **DSM** = Diagnostic Statistical Manual (any version); **ICD** = International Classification of Diseases (any version).

**Table 2:** Characteristics of the included studies.

Author (Date)	Country	Sample Characteristics		Sample Size (N)	Mean Age (± SD) or Range	Sleep Outcome	Affect/Mood Outcome	Quality
Totterdell et al. (1994) [52]	UK	Healthy	Adults	30	31.60 (range 20–59)	Sleep diary (sleep onset and offset, TST, SOL, number of awakenings), and SQ (rated on VAS)	Mood (positive and negative) rated on a VAS across 4 dimensions: energetic arousal (alert and tired), hedonic tone (cheerful and depressed), tense arousal (calm and tense), and engagement (involved and disinterested)	Good
Jones and Fletcher (1996) [53]	UK	Healthy	Adults	42	Unspecified	SQ (4 items) rated on 7-point scale	Mood (positive and negative) rated on a 7-point semantic differential scale (9 adjectives described polarised mood states, e.g., elated-depressed and dejected-cheerful). High scores indicated a negative mood	Fair
Tzischinsky et al. (2001) [54]	Israel	Shift workers (medical residents)	Adults	78	(range 26–41)	Actigraphy (TST, SE, sleep onset, and offset)	Mood (positive and negative) rated on a 4-point scale from daily questionnaire	Fair
Zohar et al. (2005) [55]	Israel	Shift workers (medical residents)	Adults	78	(range 26–39)	Actigraphy (TST) and sleep logs (number of wake-ups during the night shift). Sleep onset and offset were recorded, and SE was calculated. Fatigue (4 items) rated on the POMS subscale	Affect (positive and negative) from PANAS (4 PA items; 4 NA items)	Good
Fuligni and Hardway (2006) [56]	USA	Healthy	Adolescents	761	(range 14–15)	TST and calculated daily sleep time variability	Mood (positive and negative) from POMS selected items (anxious, depressive, happiness feelings, and fatigue)	Fair
McCrae et al. (2008) [57]	USA	Healthy	Older adults	103	72.81 (7.12)	Actigraphy (TWT) and sleep diary (SQ rated on a 5-point scale and TWT)	Affect (positive and negative) from PANAS (10 PA items; 10 NA items)	Good
Galambos et al. (2009) [58]	Canada	Healthy	Adolescents/young adults	191	18.35 (0.47) (range 17–19)	SQ (7-point scale) and TST (sleep onset and offset)	Affect (positive and negative) from PANAS (10 PA items; 10 NA items)	Fair
Cousins et al. (2011) [59]	USA	Affective disorder and HC (AD only and primary MDD with and without secondary comorbid ADs)	Children and adolescents	94 (AD only *n* = 23) (primary MDD with and without secondary comorbid ADs, *n* = 42) and (HC *n* = 29)	11.73 (range 8–16)	Actigraphy (SE, SOL, TIB, TST, and WASO)	Affect (positive and negative) from PANAS-C (4 PA items; 4 NA items)	Good
Gershon et al. (2012) [60]	USA	Affective disorder and HC (interepisode BD I)	Adults	68 (BD I *n* = 32) and (HC *n* = 36)	BD I: 34.7 (10.5) HC: 33.3 (12.6) (range 18–64)	Actigraphy and sleep diary collected the same sleep indices (SE, SOL, TIB, TST, TWT, WASO, number of awakenings, and terminal wakefulness)	Affect (positive and negative) from PANAS (10 PA items; 10 NA items)	Good
Talbot et al. (2012) [61]	USA	Affective disorder and HC (interepisode BD 1 and BD II)	Adults	101 (BD I *n* = 49) and (HC *n* = 52)	34.06 (3.45) (range 18–65)	Sleep diary (SOL, WASO, TIB, TST, sleep onset and offset, terminal wakefulness, number of awakenings), TWT, and SE (calculated)	Mood (positive and negative) adapted items from POMS-SF	Fair
de Wild-Hartmann et al. (2013) [62]	Belgium	Healthy	Adults	553 (subset from EFPTS Twin Survey)	27.77 (7.86) (range 18–61)	Sleep diary (SQ rated on a 7-point scale, SOL, number of awakenings, sleep onset and offset), TIB, and TST calculated	Affect (positive and negative) rated from 10 adjectives on a 7-point scale	Good
Lev Ari and Shulman (2013) [63]	Israel	Healthy	Adults/young adults	286	23.21 (1.75) (range 19–29)	Sleep log (TST and sleep variability)	Mood (negative only) from POMS (negative mood subscale only). Note: authors use POMS items to assess ‘daily negative affect’	Fair
Ong et al. (2013) [64]	USA	Healthy	Adults and older adults	100 (subset from MIDUS II, NSDE II, and Biomarker studies)	55.71 (12.37) (range 43–68)	Actigraphy (SE and TST) and sleep diary (SQ and morning rest level rated on 5-point scale) (MIDUS II, NSDE II, and Biomarker measures)	Affect (positive only reported in detail) adjectives (13 PA items) rated on a 5-point frequency scale (MIDUS II, NSDE II, and Biomarker measures) The study focused on PA and sleep but also recorded daily NA, trait PA (6-items from validated scales), trait NA (6-items), positive events (5 questions), and negative events (DISE measure)	Good
Doane and Thurston (2014) [65]	USA	Healthy	Adolescents	78	18.05 (0.41) (range 17–18)	Actigraphy (TST, SE, SOL, and TIB) (self-reported bed-wake times to validate actigraphy, but only actigraphy data reported)	Affect (positive and negative) from PANAS (10 PA items; 10 NA items). Stress levels and stressful events were also reported	Good
Garcia et al. (2014) [66]	USA	Healthy	Adolescents	19	15	TST (open-ended question) and SQ (yes/no response)	Affect (positive and negative) from PANAS (3 PA items; 3 NA items). Note: authors refer to PANAS ‘affect’ terms but describe these traits as ‘mood’	Fair
Kalmbach et al. (2014) [67]	USA	Healthy	Adults	171	20.07 (3.32)	PSQI (3-items related to SQ, TST, and SOL)	Affect (positive and negative) from 6 PANAS-X subscales (3 PA subscales: joviality, self-assurance, serenity) (3 NA subscales: fear, sadness, and hostility)	Good
Takano et al. (2014) [68]	Japan	Healthy	Adults/young adults	43	19.40 (1.30)	Actigraphy (SOL, SE, and TST)	Affect (positive and negative) from PANAS (3 PA items; 3 NA items)	Good
Wrzus et al. (2014) [69]	Germany	Healthy	Children and adolescents through to older adulthood	397	39.86 (20.48) (range 12–88)	TST	Affect (positive and negative) from 12 adjectives (6 PA adjectives, e.g., happy) and (6 NA adjectives, e.g., nervous) rated on a 6-point scale	Fair
Fortier et al. (2015) [70]	Canada	Healthy	Adults	63	42.60 (5.61)	Sleep satisfaction is rated from (1) very dissatisfied to (7) very satisfied	Affect (positive only) from PANAS (10 PA items)	Fair
Li et al. (2015) [71]	Canada	Healthy	Adults	19 (actigraphy *n* = 13)	34.0 (5.70) (range 18–43)	Actigraphy (SOL, TST, SE, and WASO) and self-reported DLQ items (SQ and daytime sleepiness)	Mood (positive and negative) from DLQ items and other mood questionnaires	Good
Simor et al. (2015) [72]	Hungary	Healthy	Adults/young adults	75	22.15 (2.98)	GSQS (Hungarian version; 14 items) for overall SQ	Affect (positive and negative) from PANAS (10 PA items; 10 NA items)	Good
van Zundert et al. (2015) [73]	The Netherlands	Healthy	Adolescents	286	14.19 (0.54) (range 13–16)	Sleep log (SQ rated on a 7-point scale) and number of night-time awakenings (sleep disturbance). Sleep items used in previous ESM studies, e.g., de Wild-Hartmann et al. (2013)	Affect (positive and negative) from 10 adjectives (5 PA adjectives, e.g., happy; 5 NA adjectives, e.g., anxious) on a 7-point scale. Affect items used in previous ambulatory studies	Fair
Konen et al. (2016) [74]	Germany	Healthy	Children and adolescents	110	9.88 (0.61) (range 8–11)	Sleep diary (Karolinska Sleep Diary with SQ and bed-wake times; TST calculated)	Affect (positive and negative) from 6 adjectives (3 PA items; 3 NA items) and affect arousal (6 items in total for activation/deactivation). All 12 affect state items rated on a 5-point scale	Good
McCrae et al. (2016) [75]	USA	Healthy	Adults and older adults	55	62.80 (12.21) (range 38–86)	Actigraphy (TWT) and sleep diary (TWT, SQ rated on a 5-point scale, bedtime, and time out of bed)	Affect (positive and negative) from PANAS (5 PA items; 5 NA items)	Good
Reis et al. (2016) [76]	Germany	Healthy	Adults	52	36.61 (9.98)	SQ (1-item from PSQI rated on 5-point scale)	Affect (positive and negative) dimensions are rated on 4-point intensity scales from the adapted MDBF-short. Energetic arousal (2 items, e.g., sleepy-rested), tense arousal (2 items, e.g., relaxed-nervous), and pleasant affect (4 items, e.g., unhappy-happy)	Good
Tavernier et al. (2016) [77]	USA	Healthy	Adolescents	77	14.37 (1.95) (range 11–18)	Actigraphy (TST, SOL, SE, and WASO). Study only analysed actigraphic sleep outcomes but also collected a sleep diary.	Affect (positive and negative) rated from 17 adjectives on a 5-point scale. Five dimensions from PCA analysis: negative social evaluative emotions (e.g., embarrassed), dysphoria (e.g., sad), anxious-nervous, angry-irritable, and high arousal positive affect (e.g., happy)	Fair
Blaxton et al. (2017) [78]	USA	Healthy	Adults and older adults	552	62.18 (10.42) (combined cohort range 62–91)	Sleep diary (Karolinska Sleep Diary; 5 items and sleep sufficiency item summed to assess SQ)	Affect (positive and negative) is from PANAS (10 PA items; 10 NA items) and low emotional arousal (7 items, e.g., sad)	Good
Bouwmans et al. (2017) [79]	The Netherlands	Affective disorder (MDD) and HC	Adults	54 (MDD *n* = 27) and (HC *n* = 27)	34.35 (0.35)	SQ (1-item from PghSD rated on 7-point scale). Fatigue (1-item). TST item not analysed	Affect (positive and negative) is rated from 14 adjectives (combined from PANAS and additional items; 7 PA and 7 NA)	Good
Chiang et al. (2017) [80]	USA	Healthy	Adolescents/young adults	289	16.40 (0.74) (range 15–20)	Actigraphy (sleep onset and offset, TST, SE, and TIB from the actigraph event-marker). Study only analysed actigraphic sleep outcomes but also collected a sleep diary (e.g., sleep-wake times, lights off, and time out of bed)	Mood (negative only) selected items from POMS (anxiety/depression subscales) rated on a 5-point scale. Note: authors use POMS items to assess ‘daily negative affect’	Good
Flueckiger et al. (2017) [81]	Switzerland	Healthy	Adults/young adults	596 (study 1 *n* = 292) and (study 2 *n* = 304; actigraphy *n* = 42)	Study 1: 20.70 (2.50) Study 2: 21.40 (5.10)	Study 1—SQ (1-item from PSQI rated on 5-point scale) Study 2—Actigraphy (TST), sleep log (TST, bedtimes), and SQ (1-item from PSQI, as in study 1). Study 2 only analysed self-reported sleep data (SQ) with affect outcomes	Studies 1 and 2. Affect (positive and negative) rated on a 7-point Pleasantness Scale (3 PA items; 3 NA items)	Fair
Kalmbach et al. (2017) [82]	USA	Healthy	Adults/young adults	171	20.07 (3.32)	PSQI (3-items; TST, SOL, and SQ). SQ is rated on a 4-point scale	Mood (negative only) from MASQ-SF (29 items) to assess depression/anxiety symptoms (3 subscales: general distress, anxious arousal, anhedonia)	Good
Lauritsen et al. (2017) [83]	Denmark	Affective disorder (MDD)	Adults	34	35.9 (10.8)	SQ (VAS rated from 0–10) and sleep indices (sleep onset and offset, number of awakenings, and naps)	Mood (negative/low only) rated on a VAS from 0 (worst) to 10 (best) depressive symptom severity. Mood scores < 4 (equivalent to severe depression)	Fair
McGrath et al. (2017) [84]	USA and New Zealand	Healthy	Adults and older adults	69	38 (10.91) (range 22–73)	SQ (1-item rated on a 6-point scale; based on an adapted PSQI item) and TST	Affect (positive only) from PANAS (5 PA items)	Fair
Sin et al. (2017) [85]	USA	Healthy	Adults	312 (sample 1 *n* = 131) and (sample 2 *n* = 181)	41.34 (3.21) (range 21–63)	PSQI (adapted from 2 items; SQ is rated on a 4-point scale and TST)	Affect (positive and negative) from PANAS (10 PA items; 10 NA items)	Good
Cox et al. (2018) [86]	USA	Healthy [33% met MINI criteria for a major form of psychopathology: mood (12%) and anxiety (24%) disorder]	Adults	138 (actigraphy *n* = 98)	22.48 (9.24) (range 18–64)	Actigraphy (TST) and CSD (9-items, e.g., TST, sleep onset and offset, SOL, and WASO)	Mood (low; anxiety only). Anxiety symptom ratings on a single-item scale from 0 (not anxious at all) to 100 (most anxious you could ever imagine feeling)	Good
da Estrela et al. (2018) [87]	Canada	Healthy	Adults	66	42.7 (6.89)	PSQI and ISI items (SE and 1-item SQ/sleep satisfaction rated on a 5-point scale). SE score calculated from sleep indices (e.g., TST, TIB, SOL, and WASO)	Affect (negative only) from PANAS (5 NA items). Note: authors use PANAS to assess daily negative mood	Fair
Kalmbach et al. (2018) [88]	USA	Shift workers (medical residents)	Adults	33	27.3 (2.6)	Actigraphy (TST). Also assessed were sleep timing changes across the internship (from actigraphic bed-wake times and midsleep)	Mood (high/low) ratings on a single-item scale from 1 (low) to 10 (high)	Good
Slavish et al. (2018) [89]	USA	Healthy	Adults	242	46.8 (10.9)	PROMIS sleep disturbances scale (short form adapted for daily use) for SQ and difficulty falling asleep, each rated from 0 (very poor) to 100 (very good)	Mood (negative only) from POMS (5 negative items). Note: the authors use POMS items to assess ‘the daily negative affect’	Good
George et al. (2019) [90]	USA	Healthy	Adolescents/young adults	515 (study 1 *n* = 120) and (study 2 *n* = 395; actigraphy *n* = 104)	Study 1 (range 18–19) Study 2 (range 10–16)	Study 1—Actigraphy (TST), self-reported TST (1-item) and SQ (1-item; rated 0–9) Study 2—Actigraphy (TST), self-reported TST (2-items), and SQ (1-item; rated 0–2)	Study 1—Affect (positive and negative) adjectives from adapted PANAS items and other daily measures (3 PA items; 4 NA items). Daily emotion dysregulation (adapted from ARC) rated on 4 yes/no questions. Study 2—Affect (positive and negative) from adapted PANAS items (3 PA items; 4 NA items) on a 100-point scale. Daily emotion dysregulation (adapted from ARC) is rated on a 5-point scale	Good
Leger et al. (2019) [9]	USA	Healthy	Older adults	288	73.94	TST (1-item adapted from PSQI), morning tiredness (1-item adapted from PghSD), and SQ (1-item adapted from CSD rated from 1–5)	Affect (positive and negative) adapted items (4 PA; 5 NA) from PANAS and other measures	Good
Li et al. (2019) [91]	USA	Affective disorder and HC (BD I and BD II in any mood state)	Adults/young adults	20 (BD *n* = 10) and (HC *n* = 10)	Unspecified (minimum age 18)	TST and sleep indices (e.g., sleep onset and offset, time in-out of bed, and number of awakenings)	Mood (high/low) single item rated on the VAS (converted to a 0–100 scale for analysis) for positive and negative mood valance	Fair
Merikangas et al. (2019) [92]	USA	Affective disorder and HC (BD I and BD II, MDD)	Adults	242 (BD *n* = 54) (MDD *n* = 91) and (HC *n* = 97)	48 (16.9)	Actigraphy (TST, sleep onset and offset)	Mood (positive and negative) is rated on a 7-point scale from very happy (1) to very sad (7)	Good
Parsey and Schmitter-Edgecombe (2019) [93]	USA	Healthy	Older adults	73	67.64 (9.59)	Actigraphy (SOL, SE, and WASO; as an index of SQ) and self-reported sleepiness/fatigue	Mood (positive and negative) is rated on a 5-point scale from very good (1) to very poor (5)	Fair
Triantafillou et al. (2019) [94]	USA	Healthy (depressive and anxiety scores met cut-off values for PHQ-9 and GAD-7 but was not clinically diagnosed)	Adults	206	39.3 (10.3) (range 18–66)	SQ (1-item) rated on a 9-point scale	Mood (positive and negative) is rated on a 9-point scale from 0 (extremely poor) to 8 (extremely good)	Fair
Williamson et al. (2018) [95]	Chile	Healthy	Adults	121	29.59 (6.40)	SQ (1-item from PghSD) rated on 5-point scale	Mood (positive and negative) is rated from Multi-Affect Indicator items (4 positive; 4 negative) on a 5-point scale	Fair
Das-Friebel et al. (2020) [96]	UK	Healthy	Adults/young adults	101 * * see also Lenneis et al. (2024) [97], which utilises the same dataset	19.7 (1.09) (range 18–22)	Actigraphy (TST and SE) and self-reported TST (calculated from lights off and bed-wake times) and sleep satisfaction (rated on 4-point scale)	Affect (positive and negative) adjectives from PANAS and the Circumplex Model of Affect (5 PA items; 5 NA items)	Good
O’Neill et al. (2020) [98]	USA	Shift workers (18% of sample) and healthy	Adults	324 (162 couples)	38.2 (9.10) for veterans and 36.4 (9.10) for spouses	SQ (1-item adapted from PSQI) rated on a 4-point scale	Affect (positive and negative) adjectives from various scales, e.g., PANAS-X (3 PA items; 5 NA items) rated on 5-point scale	Fair
Peltz et al. (2019) [99]	USA	Healthy	Adolescents and adults	193	Adolescents: 15.7 (0.94) (range 14–17) Parents: 47.6 (5.4)	Sleep diary for TST (calculated from sleep onset and offset) and SQ (4-item measure for overall SQ rated on a 5-point scale, SOL, restedness, and WASO)	Mood (negative only) rated from the adapted PHQ-4 for anxiety (2 items) and depressive (2 items) symptoms	Fair
Ryuno et al. (2021) [100]	Japan	Healthy (dyads of primary family caregivers and elderly care receivers living together under home care conditions)	Adults and older adults	25	66.3 (10.8) for family caregivers	Actigraphy (TST, TIB, SE, and WASO)	Affect (positive and negative) from PANAS (10 PA items; 10 NA items)	Good
Sayre et al. (2021) [101]	USA	Healthy	Adults	96	38 (9.58)	TST (1-item) (controlled for SQ in analyses; 1-item SQ rated on a 5-point scale)	Affect (positive and negative) from PANAS (3 PA items; 3 NA items)	Fair
Sin et al. (2020) [102]	USA	Healthy	Adults and older adults	1982 (subset from MIDUS II and NSDE II—data from individuals with at least 2 consecutive telephone interview days)	56.31 (12.19) (range 33–84)	TST (1-item; MIDUS II and NSDE II measures)	Affect (positive and negative) adjectives (13 PA; 14 NA) were rated on a 5-point frequency scale (MIDUS II and NSDE II measures)	Fair
Wen et al. (2020) [103]	China	Healthy	Adults/young adults	95	21.40 (2.92)	Actigraphy (TST)	Affect (positive and negative) adjectives rated from 0 (not at all) to 7 (very much) (4 PA items; 5 NA items)	Fair
Bean and Ciesla (2021) [104]	USA	Healthy	Adults/young adults	94	19.6 (1.99) (range 18–34)	TST (1-item adapted from PSQI; ‘sleep deprivation’ defined as ≤4 h of sleep) and naps	Mood (negative only) from the Mini-MASQ (26 items) to assess depressive/anxiety symptoms (3 subscales; general distress, anxious arousal, and anhedonia)	Good
Díaz-Morales and Parra-Robledo (2021) [105]	Spain	Healthy	Adolescents/young adults	111	16.1 (1.17) (range 14–20)	Sleep diary (TIB and TST calculated from bed-wake times)	Affect (positive and negative) rated from PANAS (10 PA items; 10 NA items)	Fair
Difrancesco et al. (2021) [106]	The Netherlands	Affective disorder and HC (current and remitted depressive or anxiety disorder)	Adults	359 (current affective disorder *n* = 93) (remitted affective disorder *n* = 176) and (HCs *n* = 90)	49.5 (12.6)	Actigraphy (SE and TST) and self-reported TST and SQ (rated on 7-point scale)	Affect (positive and negative) adjectives (6 PA items; 7 NA items) rated on a 7-point scale	Good
Fang et al. (2021) [107]	USA	Shift workers (medical residents)	Adults	2115	27.5 (2.4)	Actigraphy (24-h TST, SOL, WASO, and bed-wake time)	Mood (high/low) is rated on a single-item scale from 1 (lowest) to 10 (highest) mood	Fair
Jones et al. (2022) [108]	USA	Healthy	Adults	121	42.1 (12.01) (range 25–65)	Sleep indices (TST, SOL, and SQ averaged from items) from the adapted Daily Sleep Diary and PSQI items	Affect (positive and negative) adjectives from PANAS and the Circumplex Model of Affect (8 PA items; 8 NA items) rated on a 0–100 scale	Good
Lee (2022) [109]	USA	Healthy	Adults and older adults	1958 (subset from MIDUS II and NSDE II—data from individuals with at least 4 telephone interview days)	56.40 (12.15) (range 33–84)	TST and sleep loss variable calculated for TST < 6 h/night (MIDUS II and NSDE II measures)	Affect (positive and negative) adjectives rated on 4-point scale (13 PA items; 14 NA items) (MIDUS II and NSDE II measures)	Fair
Messman et al. (2021) [110]	USA	Healthy (depressive scores met cut-off values for QIDS but not clinically diagnosed)	Adults	80	32.65 (10.07)	Actigraphy (TST, SE, SOL, WASO, light levels, and event markers for bed-rise times), Z-machine (ambulatory EEG; N1-N3 sleep stages, REM sleep, TST, SOL, SE, and WASO), and CSD (SOL, WASO, terminal wakefulness, TIB, and calculated TST, TWT, and SE)	Affect (positive and negative) from PANAS (10 PA items; 10 NA items)	Good
Minaeva et al. (2021) [111]	The Netherlands	Affective disorder and HCs (MDD: currently depressed, past depressed and never depressed. Sample included 12-month follow-up)	Adults	579 (baseline) and 417 (follow-up) Subset from the EFPTS Twin Survey; at baseline, individuals with past (*n* = 82), current (*n* = 26), and without (lifetime) depression (*n* = 471). At 12-month follow-up, individuals who did (*n* = 58) and did not (*n* = 319) develop a depressive episode.	Past depression 26.93 (7.30) Present depression 28.23 (8.69) Never depressed 32.11 (9.41) Follow-up developed depression 29.41 (7.34) Follow-up non-depressed 26.89 (7.25)	Sleep diary (SQ rated on a 7-point scale, SOL, number of awakenings, bed-wake times, and calculated TIB and TST)	Affect (positive and negative) adjectives rated (4 PA items; 6 NA items) on a 7-point scale	Fair
Neubauer et al. (2021) [112]	Germany	Healthy	Children and young adolescents	192 (study 1, *n* = 108) and (study 2, *n* = 84)	Study 1: 10.11 (0.44) (range 9–11) Study 2: 8.98 (0.78) (range 8–10)	Study 1 and Study 2 had the same sleep measures. Sleep diary (items based on Karolinska Sleep Diary; 3 SQ items rated on a 5-point scale; TST operationalised as TIB). Same SQ items as Konen, Dirk, Leonhardt and Schmiedek [74]	Study 1: Affect (positive and negative) adjectives (3 PA; 3 NA) and 1-item on tense arousal affect (‘uneasy’) rated on a 5-point scale. Affect items (study 1) same as Konen, Dirk, Leonhardt and Schmiedek [74] Study 2: Affect (positive and negative) adjectives (6 PA; 6 NA) with high and low arousal	Good
Shen et al. (2022) [113]	Australia	Healthy	Adolescents	205	16.92 (0.87)	Actigraphy (TST and SE) and self-reported items (TST and SE) from the adapted CSD. SQ was operationalised as SE	Affect (positive and negative) from PANAS-X (6 PA items; 6 NA items)	Good
Shi et al. (2021) [114]	China	Healthy	Adults/young adults	64	20.92 (1.75) (range 19–26)	Actigraphy (TST)	Affect (positive and negative) from PANAS (5 PA items; 5 NA items)	Good
Simor et al. (2021) [115]	Belgium Hungary and Spain	Healthy (included clinically relevant moderate depressive symptoms)	Adults/young adults	166	28.5 (10.09) (range 18–69)	GSQS (14 items; English version translated to Spanish) for SQ and sleep disruption. Sleep schedules (bed-wake-time, SOL) are used to estimate TST	Mood (high/low) ratings on a single-item scale from 0 (extremely negative: sad, negative, distressed) to 7 (extremely positive: happy, joyful, and relaxed) State rumination (ruminative, perseverative, and intrusive thoughts) was also reported	Fair
Sin et al. (2021) [116]	USA	Healthy	Adults and older adults	1426 (subset from MIDUS II and III and NSDE II—data from individuals who reported chronic conditions at both waves and had complete data on covariates)	56.40 (12.15) (range 33–84)	TST (MIDUS II and III and NSDE II measures) Short sleep coded in 3 ways (person-centred continuous variable, ≤6 h and <7 h) and long sleep coded (≥9 h)	Affect (positive and negative). Frequency of affect emotion items rated on a 5-point scale (13 positive; 14 negative) from measures developed for MIDUS II and III and NSDE II	Fair
Sun-Suslow et al. (2021) [117]	USA	Healthy (HCs) and people with HIV (PWH)	Adults and older adults	94	(range 50–74)	Actigraphy (TST and SE) with sleep log (bed-wake time to estimate TIB) and self-reported TST and SQ (rated on 1–10 VAS)	Mood (positive and negative). Levels of happiness, depression, anxiety, and worry were reported. Happiness is rated on a 5-point scale at each EMA survey.	Fair
Vigoureux and Lee (2021) [118]	USA	Shift workers (nurses)	Adults	60	35.35 (11.83)	Actigraphy (SE, TIB, TST, and WASO) and self-reported sleep (SQ and sleep sufficiency rated on a 4-point scale, number of awakenings, SOL, bed-wake times, and daytime sleepiness)	Affect (positive and negative) rated adjectives (4 PA; 5 NA) on a 0–100 scale from Diener and Emmons (1984)	Good
Wang et al. (2021) [119]	USA	Healthy	Adults/young adults	135	19.90 (2.87)	SQ (1 item from PSQI) rated on a 4-point scale	Affect (negative only) from PANAS reported in the morning (9 NA items) and evening (10 NA items)	Fair
Wieman et al. (2022) [120]	USA	Healthy	Adults/young adults	260	18.86 (0.98)	CSD (6 items; SOL, TST, WASO, TWT, SE, and SQ rated on a 5-point scale)	Affect (positive only) ratings of state positive affect on 5-point scale (4 PA emotion items; these provided the global index of hedonia)	Fair
Wong et al. (2021) [121]	USA	Healthy	Adults	462	42.7 (7.3)	Actigraphy (TST, SE, and ‘sleep timing’ defined as midpoint between sleep onset and offset) with sleep log for wake times	Affect (positive and negative). Affect (13 items in total, with 5 PA and 7 NA listed in Table 1 of the study article) was rated on a 6-point scale. Items were from adapted PANAS-SF, POMS (2 items added), and PANAS-X (3 items added)	Good
Chan et al. (2022) [122]	Hong Kong	Healthy	Adults/young adults	59	21.7 (4.18) (range 18–35)	Sleep diary (TST, bed-wake times, and SQ rated from 1–100)	Mood (low; daily anxiety only). Daily anxiety levels rated on a single item (1–100 scale) in the morning and evening	Fair
Dickens et al. (2021) [123]	USA	Healthy	Adults/young adults	52	30 (10) (range 19–65)	SQ (adapted PSQI item) rated on a sliding scale (0–100)	Affect (only positive affect analysed) from SPANE (6 PA items) rated on a 0–100 scale	Good
Liu et al. (2022) [124]	USA	Healthy (family caregivers provided primary care to persons living with dementia ‘PLWD’ who lived in the same household)	Adults	173	61.97 (10.66) for family caregivers	Sleep diary (TIB, bed-wake times, and SQ rated on 5-point scale). SQ item from McCrae et al. (2016)	Affect (positive and negative) items adapted from the Non-Specific Psychological Distress Scale (after factor analysis, 9 PA items; 11 NA items) rated on a 5-point scale	Fair
Narmandakh et al. (2021) [125]	The Netherlands	Healthy (depressive and anxiety scores with cut-off values for DASS but not clinically diagnosed)	Adults	1165	38.6 (13.2)	SQ (2 items) rated on VAS scales (0–100)	Affect (positive and negative) adjectives (6 PA; 6 NA) and single item of worry each rated on VAS scale (0–100). Authors refer to positive/pleasant affect (PA) and negative/unpleasant affect (UA)	Fair
Parsons et al. (2022) [126]	UK	Healthy	Adults/young adults	101	21.69 (1.91) (range 18–24)	SQ (1-item from CSD rated on a 5-point scale) and TST (calculated from sleep-wake times)	Mood (positive and negative). Emotion intensity (positive/negative) rated on a 0–100 scale (“how positive/negative have you been feeling?”) and emotion duration/use of regulation strategies reported. Emotion regulation (positive; 5 items and negative; 6 items) also assessed	Fair
Song et al. (2021) [127]	China	Healthy	Adults	213	31.78 (8.51)	SQ (4-item adapted scale)	Affect (positive and negative) from PANAS-SF (6 PA; 6 NA). PA items used as a control variable	Fair
Sperry and Kwapil (2022) [128]	USA	Healthy (including heightened risk for BSDs)	Adults/young adults	233	18.81 (1.04)	Sleep diary (3 items adapted from PSQI; bed-wake times and SQ is rated on a 4-point scale) and TST calculated	Affect (positive and negative) adjectives rated on a 7-point scale (4 PA; 7 NA)	Fair
Ying et al. (2021) [129]	Canada and USA	Healthy	Adults/young adults	1233 (community sample *n* = 911, student sample *n* = 322)	Community sample: 46.52 (16.02) Student sample: 21.20 (3.89)	CSD (bed-rise times, WASO, SOL, TIB, SQ rated on 0–100 VAS, and TST calculated)	Affect (positive and negative) adjectives (9 PA; 7 NA) rated on a VAS (0–100) with items adapted from the NSDE (MIDUS study; see Sin et al. 2020)	Good
Harris et al. (2022) [130]	USA	Healthy	Adults/young adults	109	20.67 (0.49) (range 20–22)	Sleep diary (5 standardised items; TST, SOL, WASO, number of awakenings; and SQ is rated on a 4-point scale). Items are averaged to create an overall daily SQ score	Affect (positive and negative) adjectives (5 PA; 7 NA) rated on a 5-point scale with items adapted from mDES	Good
Hruska et al. (2022) [131]	USA	Healthy	Adults	79	30.72 (9.38)	SQ (1 item from CSD rated on a 5-point scale)	Mood (negative only). Anger (5 items, e.g., “you felt angry/grouchy today”) adapted from the PROMIS subscale rated on a 5-point scale	Fair
Lee et al. (2023) [132]	USA	Healthy	Adults	20	37.68 (4.55)	Sleep disturbance (7 items from ISI, e.g., SOL, sleep satisfaction, sleep maintenance). Sleepiness and fatigue (DISS items)	Mood (positive and negative) rated on a 5-point scale from DISS items (“How stressed/happy do you feel right now?”)	Good
Marcusson-Clavertz et al. (2022) [133]	USA	Healthy (the SHADE study included asthma and rheumatoid arthritis)	Adults	523 ESCAPE (*n* = 234) SHADE (*n* = 117) SAWM (*n* = 172)	ESCAPE 46.9 (10.9) SHADE 44.5 (13.8) SAWM 49.5 (16.9)	ESCAPE (TST, difficulty falling asleep, and SQ rated on VAS 0–100) SHADE (TST and SQ rated on a 7-point scale) SAWM (TST, SOL, and SQ is rated on a 4-point scale)	Affect (negative only) adjectives were used across all 3 studies and stressful event occurrences reported. ESCAPE (5 NA items rated 0–100 on VAS) SHADE (5 NA items rated on a 7-point scale) SAWM (4 NA items rated from 0–7)	Fair
Mousavi et al. (2022) [134]	USA	Healthy	Adults/young adults	20	19.80 (1.0)	Sleep tracker device (Oura ring; TST, WASO, SE, and SOL)	Affect (positive and negative) items from PANAS (10 PA; 10 NA) rated on a 0–100 scale	Good
Newman et al. (2022) [135]	USA	Healthy (depressive scores met cut-off values for QIDS but were not clinically diagnosed)	Adults	181	41.91 (5.06)	Actigraphy (TST, SE) and sleep diary (TST, SE, TIB, and SQ is rated on a 4-point scale)	Affect (positive and negative) adjectives rated in morning and evening (overall 12 PA; 12 NA each day) rated on a 5-point scale. Evening affect items from mDES	Good
Patapoff et al. (2022) [136]	USA	Affective disorder (BD; not in acute manic or depressive episodes)	Adults	56	47.3 (9.2)	Actigraphy (TST, SE, sleep onset time, and WASO) with sleep diary (bed-rise times and TST), and SQ (rated on a 4-point scale)	Mood (negative only). Intensity of moods (4 items, e.g., sadness, anxiety) rated on a 7-point scale	Good
Peltz and Rogge (2022) [137]	USA	Healthy	Adolescents and adults	386 (adolescents and parent dyads *n* = 193)	Adolescents 15.7 (0.94) Parents 47.6 (5.4)	Sleep diary (4 item measure for SQ; SOL, WASO, restedness and overall SQ rated on a 5-point scale), TIB, and TST calculated	Mood (negative only). Depressive/anxiety symptoms from adapted PHQ-4 items (rated on a 4-point scale)	Fair
Roberts et al. (2022) [138]	USA	Healthy	Adults	225	(range 23–67)	Sleep diary (bed-wake times, SQ rated 0–10)	Mood (positive and negative). Morning and evening mood states (calm, happy, relaxed, and angry/irritable) are rated on a 5-point scale. Positive and negative events (10 items) were also reported	Fair
Shi and Wang (2022) [139]	China	Healthy	Adults	98	Unspecified (majority 25–34 years)	SQ (3 items rated on a 5-point scale)	Affect (negative only) from PANAS-SF (6 NA items)	Fair
Titone et al. (2022) [140]	USA	Affective disorder (recent-onset BSD and high-risk for BSD)	Adults/young adults	107	21.82	Actigraphy (SOL and TST)	Mood (positive and negative). Depressive (4 items, e.g., sad, hopeless) and hypomania (4 items, e.g., happy, self-confident) symptoms were reported on a 5-point scale. Items from ASRM and BDI-II measures	Good
Tseng et al. (2022) [141]	Taiwan	Affective disorder (BD)	Adults	159	34.5 (11.34)	TST and bed-wake times	Mood (high/low). Mood scores are rated daily from 0 (low) to 6 (high/better mood). At weekly intervals, depressive, anxiety, and manic symptoms were reported from ASRM and DASS-21	Fair
Wang et al. (2023) [142]	USA	Healthy	Adolescents	546	15	SQ (1 item from PSQI) rated on a 5-point scale	Mood and Affect (positive and negative). Affect items from PANAS-C rated (4 PA; 4 NA). Mood during the past 24 h rated on a 5-point scale	Fair
Yip et al. (2022) [143]	USA	Healthy	Adolescents	350	14.27 (0.61) (range 13–17)	Sleep-wake problems self-reported; adapted PSQI items measured night-time disturbance (8 items) and daytime dysfunction (2 items). Day-time sleepiness (1-item from SSS)	Mood (positive and negative) from adapted POMS. Daily negative mood (4 items), anxious mood (4 items), and positive mood (4 items) are rated on a 5-point scale. Mood states and symptoms over the past 2 weeks were reported at the end of the study	Fair
Kouros et al. (2022) [144]	USA	Healthy	Adolescents	311 (4th wave of the Auburn University Sleep Study)	17.37 (0.87)	Actigraphy (TST, SE and event-marker to indicate first attempt to fall asleep) with sleep diaries (TST, bed-rise times, sleep-wake problem composite score, and SQ rated on a 5-point scale)	Mood (positive and negative). Negative mood (6 items, e.g., sad and angry. Composite summed score) and positive mood (1 item; happiness), both rated on a 5-point Likert scale	Good
Lucke et al. (2022) [145]	Germany	Healthy	Older adults	325 (pooled data from two studies)	Sample 1 EMIL Study included 120 young–old adults: 67.2 (0.9), range (66–69), and 45 old–old adults: 86.7 (1.5), range (84–90) Sample 2 Socioeconomic Panel included 160 adults: 71.8 (5.8), range (61–88)	SQ (slider from 0 = extremely bad to 100 = extremely good)	Affect (negative only) (6 adjectives, e.g., angry, sad) is rated on a slider scale (0–100). Mean overall affect score for baseline negative affect (situations without a previous stressor) and affective reactivity (increase in negative affect in situations with vs. without previous stressors)	Fair
Sheppard et al. (2022) [146]	USA	Healthy	Adults	135	39.6 (6.50)	SQ (rated 1–5) and TST	Affect (positive only) from PANAS (10 PA items) rated on 5-point scale	Good
Barber et al. (2023) [147]	USA	Healthy	Adults	2804 (subset from two MIDUS samples). Sample 1 (*n* = 2022) and Sample 2 (*n* = 782)	Sample 1 MIDUS I and II NSDE at time of data collection: 56.2 (12.2), range (33–84) Sample 2 MIDUS Refresher Study and NSDE: 47.9 (12.7), range (25–74)	TST (MIDUS and NSDE measure)	Affect (negative only) items adapted from PANAS and the Non-Specific Psychological Distress Scale for the MIDUS study (14 negatively valenced emotions, e.g., nervous, rated on their frequency on a 5-point scale. Scores averaged across items)	Fair
Chachos et al. (2023) [148]	Australia	Healthy	Adolescents/young adults	468 (205 adolescents; 263 emerging adults) from three projects (STEPS, ACES, DESTRESS)	Adolescents: 16.92 (0.87) Young adults: 21.29 (1.73)	Actigraphy (TST and SE) and CSD (TST and SE)	Affect (positive and negative) from PANAS-X (12 items) rated on 5-point Likert scale. Four separate affect domains derived (low-high PA/NA arousal)	Good
Hachenberger et al. (2023) [149]	Germany	Healthy	Adults/young adults	147	22.6 (1.9), range (18–25)	SOL and TST. SQ (rated on VAS 0–100; 1 item adapted from GSQS)	Affect (positive and negative) from PANAS and Russell’s Circumplex Model of Affect items (5 PA; 5 NA rated on VAS 0–100). Scores summed for PA and NA. Affect measures follow Das-Friebel et al.’s (2020) study	Good
Jordan et al. (2023) [150]	USA	Shift workers (nurses)	Adults	401	39.47	CSD (TST, SE, SOL, WASO, terminal wakefulness, and midpoint of sleep)	Mood (negative only) from 1-item on depressed mood, “I felt down, depressed, or hopeless” rated on a 5-point Likert scale	Good
Kirshenbaum et al. (2023) [151]	USA	Healthy	Adolescents	89 (main analyses *n* = 87)	15.51 (1.37), range (13–20). Main analyses with 13–18 years old	Actigraphy (TST and SE with event markers) and sleep diary (TST coded, SQ rated from 1–100)	Affect (positive and negative) items adapted from prior studies* (6 NA; 4 PA). Ratings averaged for aggregate scores of negatively and positively valenced items * [152,153,154,155,156]	Good
Master et al. (2023) [157]	USA	Healthy	Adolescents	525 (subsample from the Fragile Families and Child Wellbeing Study)	15.4 (0.5)	Actigraphy (TST awakenings, sleep-wake times, and sleep maintenance efficiency)	Mood (positive and negative) is rated on a 5-point Likert scale across 3 items. Mood items similar to POMS subscales and prior study items, e.g., Fuligni and Hardway (2006)	Good
McGowan et al. (2023) [158]	USA	Healthy	Adults/young adults	226 (data from the Social Health Impact of Network Effects study)	20.2 (1.7), range (18–42)	TST and SQ rated from 1–100 (adapted PSQI items for daily assessment)	Mood (positive and negative) items from POMS (1 PA; 3 NA) rated on a sliding scale 1–100	Good
Ng et al. (2023) [159]	Singapore	Healthy	Adults/young adults	119	22.54 (1.74), range (21–30)	Actigraphy (TIB, TST, SOL, WASO, SE, mid-sleep times, and bed-wake times) and self-report bed-wake times (only to check sleep periods from Oura Ring) and reported naps	Mood (positive and negative) is rated on a sliding scale (0–100) “How are you feeling right now?”	Good
Ohana and Fortin (2023) [160]	USA	Healthy	Adults	251	45.6 (11.3), range (23–70)	SQ (3 items from the Sleep Quality Index) [161]	Affect (negative only) from PANAS items	Fair
Punna et al. (2023) [162]	Finland	Shift workers (nonstandard hours in retail/services sectors, e.g., hotels, stores and service stations)	Adults	29	35 (11)	TST (1 item)	Mood (positive and negative) items rated on a Likert scale (3 NA; 2 PA)	Fair
Rea et al. (2023) [163]	USA	Healthy	Adults	244	18.9	TST and calculated TST variability	Affect (positive and negative) from PANAS (10 PA; 10 NA) is rated on a 5-point scale. Affect variability is also calculated	Fair
Sell et al. (2023) [164]	Canada and the UK	Healthy	Adults	Study 1 (*n* = 111 couples) Study 2 (*n* = 100 couples)	Study 1: 26.76 (7.17), range (18–57) Study 2: 24.15 (6.61), range (18–64)	Study 1 and 2—SQ (4-point scale) and TST	Study 1—Affect (negative only) 3 emotions rated on a 7-point scale. Averaged into a negative affect composite Study 2—Affect (negative only) 3 emotions rated on a 5-point scale. Averaged into a negative affect composite	Good
Song et al. (2023) [165]	USA	Affective disorder and HCs (GAD, MDD, GAD, MDD, and HCs)	Adults	80 * * GAD (*n* = 23) MDD (*n* = 11) GAD and MDD (*n* = 11) HCs (*n* = 35)	GAD: 33.61 (12.13) MDD: 42.80 (15.80) GAD and MDD: 30.55 (13.16) HCs: 28.94 (12.54)	TST and SQ (rated on VAS 0–100)	Mood (items covering DSM-5 symptoms for MDD and GAD) is rated on the VAS (0–100). Affect (positive and negative) items (4 PA; 7 NA). Daily affect averaged over PA and NA items and affect variability calculated	Good
Xie et al. (2023) [166]	USA	Healthy	Older adults	238 couples (daily diaries for a total sample of 476 individual participants)	70 (6.7)	SQ rated on the slider (0–100)	Affect (positive and negative) rated on slider 0–100 (3 NA; 3 PA). Averaged scores for composite NA and PA	Fair
Baglioni et al. (2024) [167]	Germany	Healthy (sample had controls with no sleep or health issues)	Adults	Controls * (*n* = 86) Insomnia (*n* = 97) * included good sleepers under self-imposed sleep restrictions (*n* = 41) and good sleepers with the usual amount of sleep (*n* = 45)	Good sleeper with sleep restriction: 35.89 (13.77) Good sleeper with usual amount of sleep: 37.39 (15.62) Insomnia: 38.32 (17.10)	Sleep diaries (SOL, WASO, SE index, TST, and TIB). Also collected heart rate from ECG sensors (EcgMove 3, Movisens).	Affect (positive and negative) from PANAS (18 items), and positive and negative feelings rated 1–5	Good
Collier Villaume et al. (2024) [168]	USA	Healthy (including individuals at risk for major depressive episodes; MDE)	Adolescents/young adults	139 * * draws on multiple waves of data from the Youth Emotion Project	18.97 (0.42)	Actigraphy (TST and TIB) and self-report PSQI (TIB and TST)	Affect (positive and negative). High arousal NA (e.g., worried), low arousal NA (e.g., sad). High arousal PA (e.g., alert) and low arousal PA (e.g., happy)	Good
Dong et al. (2024) [169]	USA	Healthy	Children and young adolescents	142	14.03 (1.37), range (12–16)	Actigraphy (TST and SE) and sleep diary (SQ rated 0–100, bed-wake times)	Mood (positive to negative) rated from 0 (sad) to 100 (happy)	Good
Evans et al. (2024) [170]	USA	Healthy (included youth at high or low familial risk for psychopathology)	Children and young adolescents	82 youths * * at high (*n* = 41) or low (*n* = 41) familial risk for psychopathology	11.12 (1.46), range (9–13)	Sleep diary (SQ rated on a sliding scale of 0–100) (TST and bed-wake times are not analysed)	Affect (positive and negative) items (8 NA; 8 PA) are rated on a sliding scale (0–100). Affect averaged for NA/PA items—means, peaks, and variability calculated	Fair
Kwan et al. (2024) [171]	China	Healthy	Adults	160	85% of sample ≤ 35	SQ (3 items, including awakenings)	Affect (negative only) 5 items rated on a 5-point Likert scale	Fair
Lee et al. (2024) [172]	USA	Affective disorder and HCs (MDD)	Adults	2012 (subset from MIDUS and NSDE II]	56.51 (12.15), range (33–84)	TST	Affect (positive and negative) items (13 PA; 14 NA) rated on a 5-point scale. Average scores across PA/NA items calculated	Fair
Meigs et al. (2024) [173]	USA	Affective disorder and HCs (primary diagnosis of disruptive mood dysregulation disorder (DMDD), ADHD, anxiety disorder, or HCs)	Children and young adolescents	125 * * DMDD (*n* = 37) ADHD (*n* = 33) Anxiety disorder (*n* = 28) HCs (typically developing youth) (*n* = 27)	12.58 (2.56), range (8–18)	TST and bed-wake times	Mood (negative only) irritability symptoms via 4 items (anger, frustration, grouchiness, and mood change) rated on 5-point Likert scales	Fair
Peng et al. (2024) [174]	China	Healthy (met cut-off values for GAD-7 but was not clinically diagnosed)	Adults	115	19.60 (1.02)	Sleep disturbance (1-item based on GAD criteria: difficulty falling or staying asleep or restless and unsatisfying sleep)	Mood (negative only) based on DSM-5 GAD criteria (e.g., excessive worry, uncontrollable worry, restlessness, fatigue, irritability) rated on a Likert scale from 1–7	Fair
Poon et al. (2024) [175]	Hong Kong	Affective disorder and HCs (MDD: currently depressed)	Adults	150 * *MDD (*n* = 75) HCs (*n* = 75)	All participants: 30.3, range (8–64) MDD: 30.4 (11.2) HCs: 30.2 (11.1)	Actigraphy (TST, SE, SOL, TIB, WASO). Self-reported bed-wake times and event marker were used to adjust the actigraphy. Study only analysed actigraphy data	Mood (positive and negative) items from DISS rated on VAS (positive mood—relaxed, energetic, calm, happy, and efficient; negative mood—anxious, stressed, tense, sad, and irritable)	Good
Wescott et al. (2024) [176]	USA	Affective disorder and HCs (varying levels of depression: seasonal depression, subsyndromal seasonal depression, non-seasonal depression, and controls)	Adults	91 * * includes seasonal depression (*n* = 26), subsyndromal seasonal depression (*n* = 25), non-seasonal depression (*n* = 22), and HCs (*n* = 18) * 73 had actigraphy, and ≥3 days of morning affect data	All participants: 42.1 (13.3) Seasonal depression: 42.7 (13.2) Subsyndromal seasonal depression: 38.8 (12.8) Non-seasonal depression: 40.8 (14.5) HCs: 48.2 (11.5)	Actigraphy (TST and sleep midpoints, event markers for sleep initiation) and PghSD (sleep-wake times to cross-reference actigraphy and SQ rated on VAS 0–100)	Affect (positive and negative scale) from PghSD rated on VAS 0–100 (“Very tense” to “Very calm”)	Good
Xie et al. (2024) [177]	China	Healthy	Children and young adolescents	273	11.57 (1.31)	TST, SQ (rated on a 4-point scale), SOL, and sleep disturbance (adapted from PSQI)	Affect (positive and negative) from PANAS-C items (5 PA; 5 NA) rated on a 5-point scale. Average affect scored	Good
Zapalac et al. (2024) [178]	USA	Healthy	Adults	82	21.40 (3.21), range (18–35 years)	Actigraphy (TST, WASO, SE, sleep stages, e.g., minutes of light-deep sleep, REM, REM onset latency; NREM sleep variable summed) and self-reported sleep (TST, SOL, awakenings, and SQ rated 0–3)	Mood (positive and negative) rated on a 5 dimensions through Likert scales	Good

**AD** = Anxiety Disorder; **ARC** = Affect Regulation Checklist; **ASRM** = Altman Self-Rating Mania Scale; **BD I and BD II** = Bipolar Disorders Type I and Type II; **BDI-II** = Beck Depression Inventory-II; **BSDs** = Bipolar Spectrum Disorders; **CSD** = Consensus Sleep Diary; **DASS-21** = Depression, Anxiety, and Stress Scale; **DISE** = Daily Inventory of Stressful Events; **DISS** = Daytime Insomnia Symptom Scale; **DLQ** = Daily Life Questionnaire; **EEG** = Electroencephalogram; **GAD-7** (Generalised Anxiety Disorder Scale-7); **GSQS** = Groningen Sleep Quality Scale; **HC** = Healthy Controls; **ISI** = Insomnia Severity Index; **MASQ** = Mood and Anxiety Symptom Questionnaire (Mini-MASQ; MASQ-SF); **MDBF** = Multidimensional Mood State Questionnaire-Short; **MDD** = Major Depressive Disorder; **mDES** = Modified Differential Emotions Scale; **MINI** = Mini-International Neuropsychiatric Interview; **PANAS** = Positive and Negative Affect Schedule (PANAS-C; PANAS-X; PANAS-SF); **PghSD** = Pittsburgh Sleep Diary; **PHQ** = Patient Health Questionnaire; **POMS** = Profile of Mood States (POMS-SF); **PROMIS** = Patient-Reported Outcomes Measurement Information System; **PSQI** = Pittsburgh Sleep Quality Index; **QIDS** = Quick Inventory of Depressive Symptomatology; **SE** = Sleep Efficiency; **SOL** = Sleep Onset Latency; **SPANE** = Scale of Positive and Negative Experience; **SQ** = Sleep Quality; **SSS** = Stanford Sleepiness Scale; **TIB** = Time In Bed; **TST** = Total Sleep Time/Sleep Duration; **TWT** = Total Wake Time; **VAS** = Visual Analogue Scale; **WASO** = Time Awake After Sleep Onset.

## Data Availability

The data supporting the conclusions of this article will be made available by the authors on request.

## Supplementary Materials

The following supporting information can be downloaded at: https://www.mdpi.com/article/10.3390/s24144701/s1, Table S1: PRISMA 2020 Statement and Checklist; Table S2: Systematic search strategy; Table S3: Records excluded at full-text screening; Table S4: Data extraction categories; Table S5: Standardised affective state measures; Table S6: Number and timing of self-report assessments.
