# Supplementary material for: Monitoring Daily Sleep, Mood, and Affect Using Digital Technologies and Wearables: A Systematic Review
Source: Sensors (Basel). 2024 Jul 19;24(14):4701. doi: 10.3390/s24144701 (PMC11280943; doi:10.3390/s24144701)
Supplement: Supplementary file 1 [file sensors-24-04701-s001.zip › sensors-3011073-supplementary.pdf]

sensors

MDPI

*Systematic Review*

# Monitoring daily sleep, mood and affect using digital technologies and wearables: A systematic review

## Supplementary Materials

**Table S1.** PRISMA 2020 Statement and Checklist

**Table S2.** Systematic search strategy

**Table S3.** Records excluded at full-text screening

**Table S4.** Data extraction categories

**Table S5.** Standardised affective state measures

**Table S6.** Number and timing of self-report assessments

sensors

MDPI

**Table S1.** PRISMA 2020 Statement and Checklist.

## PRISMA 2020 Checklist

| Section and Topic | Item # | Checklist item | Location where item is reported |
|---|---|---|---|
| **TITLE** | | | |
| Title | 1 | Identify the report as a systematic review. | Page 1 |
| **ABSTRACT** | | | |
| Abstract | 2 | See the PRISMA 2020 for Abstracts checklist. | Page 1 |
| **INTRODUCTION** | | | |
| Rationale | 3 | Describe the rationale for the review in the context of existing knowledge. | Page 2 |
| Objectives | 4 | Provide an explicit statement of the objective(s) or question(s) the review addresses. | Pages 1 & 2 |
| **METHODS** | | | |
| Eligibility criteria | 5 | Specify the inclusion and exclusion criteria for the review and how studies were grouped for the syntheses. | Pages 3-5 & Table 1 |
| Information sources | 6 | Specify all databases, registers, websites, organisations, reference lists and other sources searched or consulted to identify studies. Specify the date when each source was last searched or consulted. | Pages 2-3 |
| Search strategy | 7 | Present the full search strategies for all databases, registers and websites, including any filters and limits used. | Supplementary Table S2 |
| Selection process | 8 | Specify the methods used to decide whether a study met the inclusion criteria of the review, including how many reviewers screened each record and each report retrieved, whether they worked independently, and if applicable, details of automation tools used in the process. | Pages 4-5 |
| Data collection process | 9 | Specify the methods used to collect data from reports, including how many reviewers collected data from each report, whether they worked independently, any processes for obtaining or confirming data from study investigators, and if applicable, details of automation tools used in the process. | Pages 3-5 and Supplementary Table S4 |
| Data items | 10a | List and define all outcomes for which data were sought. Specify whether all results that were compatible with each outcome domain in each study were sought (e.g. for all measures, time points, analyses), and if not, the methods used to decide which results to collect. | Supplementary Table S4 |
| | 10b | List and define all other variables for which data were sought (e.g. participant and intervention characteristics, funding sources). Describe any assumptions made about any missing or unclear information. | Table 2 & Supplementary Table S4 |

| Section and Topic | Item # | Checklist item | Location where item is reported |
|---|---|---|---|
| Study risk of bias assessment | 11 | Specify the methods used to assess risk of bias in the included studies, including details of the tool(s) used, how many reviewers assessed each study and whether they worked independently, and if applicable, details of automation tools used in the process. | Page 5 |
| Effect measures | 12 | Specify for each outcome the effect measure(s) (e.g. risk ratio, mean difference) used in the synthesis or presentation of results. | N/A |
| Synthesis methods | 13a | Describe the processes used to decide which studies were eligible for each synthesis (e.g. tabulating the study intervention characteristics and comparing against the planned groups for each synthesis (item #5)). | Pages 4-5 & Table 2 |
| | 13b | Describe any methods required to prepare the data for presentation or synthesis, such as handling of missing summary statistics, or data conversions. | Pages 2-6 |
| | 13c | Describe any methods used to tabulate or visually display results of individual studies and syntheses. | Pages 6-31 & Figures 2 - 4 |
| | 13d | Describe any methods used to synthesize results and provide a rationale for the choice(s). If meta-analysis was performed, describe the model(s), method(s) to identify the presence and extent of statistical heterogeneity, and software package(s) used. | N/A |
| | 13e | Describe any methods used to explore possible causes of heterogeneity among study results (e.g. subgroup analysis, meta-regression). | N/A |
| | 13f | Describe any sensitivity analyses conducted to assess robustness of the synthesized results. | N/A |
| Reporting bias assessment | 14 | Describe any methods used to assess risk of bias due to missing results in a synthesis (arising from reporting biases). | N/A |
| Certainty assessment | 15 | Describe any methods used to assess certainty (or confidence) in the body of evidence for an outcome. | Page 5 |
| **RESULTS** | | | |
| Study selection | 16a | Describe the results of the search and selection process, from the number of records identified in the search to the number of studies included in the review, ideally using a flow diagram. | Figure 1 & Page 5 |
| | 16b | Cite studies that might appear to meet the inclusion criteria, but which were excluded, and explain why they were excluded. | Supplementary Table S3 |
| Study characteristics | 17 | Cite each included study and present its characteristics. | Table 2 |
| Risk of bias in studies | 18 | Present assessments of risk of bias for each included study. | Table 2 |
| Results of individual studies | 19 | For all outcomes, present, for each study: (a) summary statistics for each group (where appropriate) and (b) an effect estimate and its precision (e.g. confidence/credible interval), ideally using structured tables or plots. | N/A |
| Results of syntheses | 20a | For each synthesis, briefly summarise the characteristics and risk of bias among contributing studies. | Page 5 & Table 2 |
| | 20b | Present results of all statistical syntheses conducted. If meta-analysis was done, present for each the summary estimate and its precision (e.g. confidence/credible interval) and measures of statistical heterogeneity. If comparing groups, describe the direction of the effect. | Pages 30-47 |
| | 20c | Present results of all investigations of possible causes of heterogeneity among study results. | N/A |
| | 20d | Present results of all sensitivity analyses conducted to assess the robustness of the synthesized results. | N/A |

| Section and Topic | Item # | Checklist item | Location where item is reported |
|---|---|---|---|
| Reporting biases | 21 | Present assessments of risk of bias due to missing results (arising from reporting biases) for each synthesis assessed. | N/A |
| Certainty of evidence | 22 | Present assessments of certainty (or confidence) in the body of evidence for each outcome assessed. | Page 5 & Table 2 |
| **DISCUSSION** | | | |
| Discussion | 23a | Provide a general interpretation of the results in the context of other evidence. | Pages 42 - 50 |
| | 23b | Discuss any limitations of the evidence included in the review. | Page 49 |
| | 23c | Discuss any limitations of the review processes used. | Page 49 |
| | 23d | Discuss implications of the results for practice, policy, and future research. | Page 48 - 50 |
| **OTHER INFORMATION** | | | |
| Registration and protocol | 24a | Provide registration information for the review, including register name and registration number, or state that the review was not registered. | Review was not registered |
| | 24b | Indicate where the review protocol can be accessed, or state that a protocol was not prepared. | Available on request |
| | 24c | Describe and explain any amendments to information provided at registration or in the protocol. | Review was not registered |
| Support | 25 | Describe sources of financial or non-financial support for the review, and the role of the funders or sponsors in the review. | Page 50 |
| Competing interests | 26 | Declare any competing interests of review authors. | Page 50 |
| Availability of data, code and other materials | 27 | Report which of the following are publicly available and where they can be found: template data collection forms; data extracted from included studies; data used for all analyses; analytic code; any other materials used in the review. | N/A |

**Table S2.** Systematic search strategy.

| **Database** | Full search strategies across each database. Search date to **28th May 2024** |
|---|---|
| **APA PsycINFO** | **Database: APA PsycInfo <1806 to May Week 4 2024>**<br>**Search Strategy:**<br>**1** sleep*.id. or (sleep* or asleep or slept).ab,ti. or exp Sleep/ (104386)<br>**2** (mood*1 or emotion* or affect*).id. or (mood* or emotion* or affect or affective or affectivity or well-being or wellbeing).ab,ti. or exp Emotions/ (1076927)<br>**3** (ecological momentary or experience sampling or diary or diaries).id. or (ecological momentary or experience sampling or (ambulatory adj (assessment or study)) or intensive longitudinal or event sampling or ((diary or diaries) adj2 (study or structured or ambulatory or daily or electronic or online)) or real-time or momentary or (daily adj (experience*1 or event*1 or variability)) or day-to-day).ab,ti. or exp Ecological Momentary Assessment/ or *journal writing/ or *diary measure/ (47821)<br>**4** 1 and 2 and 3 (862) |
| **Embase** | **Database: Embase <1974 to 2024 May 24>**<br>**Search Strategy:**<br>**1** sleep*.kw. or (sleep* or asleep or slept).ab,ti. or exp sleep/ (450615)<br>**2** (mood*1 or emotion* or affect*).kw. or (mood* or emotion* or affect or affective or affectivity or well-being or wellbeing).ab,ti. or exp emotion/ (2251816)<br>**3** (ecological momentary or experience sampling or diary or diaries).kw. or (ecological momentary or experience sampling or (ambulatory adj (assessment or study)) or intensive longitudinal or event sampling or ((diary or diaries) adj2 (study or structured or ambulatory or daily or electronic or online)) or real-time or momentary or (daily adj (experience*1 or event*1 or variability)) or day-to-day).ab,ti. or exp ecological momentary assessment/ (536354)<br>**4** 1 and 2 and 3 (2163) |
| **Ovid MEDLINE(R)** | **Database: Ovid MEDLINE(R) ALL <1946 to May 24, 2024>**<br>**Search Strategy:**<br>**1** sleep*.kw. or (sleep* or asleep or slept).tw. or exp Sleep/ (268307)<br>**2** (mood*1 or emotion* or affect*).kw. or (mood* or emotion* or affect or affective or affectivity or well-being or wellbeing).tw. or exp Emotions/ (1633627)<br>**3** (ecological momentary or experience sampling or diary or diaries).kw. or (ecological momentary or experience sampling or (ambulatory adj (assessment or study)) or intensive longitudinal or event sampling or ((diary or diaries) adj2 (study or structured or ambulatory or daily or electronic or online)) or real-time or momentary or (daily adj (experience*1 or event*1 or variability)) or day-to-day).tw. or exp Ecological Momentary Assessment/ (396867)<br>**4** 1 and 2 and 3 (1091) |
| **Scopus** | **1** ( AUTHKEY ( sleep* ) OR TITLE-ABS ( sleep* OR asleep OR slept ) OR INDEXTERMS ( sleep* ) ) AND ( AUTHKEY ( mood OR emotion* OR affect* ) OR TITLE-ABS ( mood* OR emotion* OR well-being OR wellbeing ) OR TITLE-ABS ( {affect} OR {affective} OR {affectivity} ) OR INDEXTERMS ( mood OR emotion* OR affect* ) ) AND ( AUTHKEY ( "ecological momentary" OR "experience sampling" OR diary ) OR TITLE-ABS ( "ecological momentary" OR "experience sampling" OR {ambulatory assessment} OR {ambulatory study} OR "intensive longitudinal" OR "event sampling" OR real-time OR momentary OR day-to-day ) OR TITLE-ABS ( daily PRE/0 ( experience OR event OR variability ) ) OR TITLE-ABS ( diary W/1 ( study OR structured OR ambulatory OR daily OR electronic OR online ) ) OR INDEXTERMS ( "ecological momentary" OR "experience sampling" OR diary ) )<br>**2** (1,569 document results) |

**Table S3.** Records excluded at full-text screening.

| | Records excluded at full-text screening, Author (Date) | Reference | Exclusion Reason |
|---|---|---|---|
| 1. | Berry and Webb (1985) | [1] | Wrong study design |
| 2. | Bower et al. (2010) | [2] | Wrong study outcomes |
| 3. | Buysse et al. (2007) | [3] | No control group data |
| 4. | Casement et al. (2021) | [4] | No control group data |
| 5. | Elovainio et al. (2020) | [5] | Wrong study outcomes |
| 6. | Kammerer et al. (2021) | [6] | Reported statistical analyses |
| 7. | Kaufmann et al. (2016) | [7] | Wrong study design |
| 8. | Kouros and El-Sheikh (2015) | [8] | Wrong study design |
| 9. | Lemola et al. (2013) | [9] | Wrong study design |
| 10. | O'Leary et al. (2016) | [10] | Multiple reports of the same sample |
| 11. | Peltz (2022) | [11] | Wrong study outcomes |
| 12. | Pressman et al. (2017) | [12] | Wrong study design |
| 13. | Strahler et al. (2020) | [13] | Wrong study outcomes |
| 14. | Thota (2020) | [14] | Wrong study outcomes |
| 15. | Totterdell et al. (1995) | [15] | Reported statistical analyses |
| 16. | Coifman et al. (2021) | [16] | Wrong study design |
| 17. | Dong and Zhang (2021) | [17] | Reported statistical analyses |
| 18. | Gumport et al. (2021) | [18] | Reported statistical analyses |
| 19. | Hussain et al. (2021) | [19] | Reported statistical analyses |
| 20. | Hyde et al. (2021) | [20] | Reported statistical analyses |
| 21. | Kaurin et al. (2022) | [21] | No control group data |
| 22. | Lee et al. (2020) | [22] | Wrong study design |
| 23. | Moshe et al. (2021) | [23] | Reported statistical analyses |
| 24. | Muzni et al. (2020) | [24] | Wrong study design |
| 25. | Philbrook and Macdonald-Gagnon (2021) | [25] | Wrong study outcomes |
| 26. | Mousa et al. (2021) | [26] | No control group data |
| 27. | Saksvik-Lehouillier et al. (2021) | [27] | Reported statistical analyses |
| 28. | Sano et al. (2017) | [28] | Wrong publication type |
| 29. | Seidl et al. (2021) | [29] | Reported statistical analyses |
| 30. | Shen et al. (2021) | [30] | Wrong study outcomes |
| 31. | Nater-Mewes et al. (2021) | [31] | Wrong population |
| 32. | Wu et al. (2021) | [32] | Reported statistical analyses |
| 33. | Zamora et al. (2022) | [33] | Wrong study outcomes |
| 34. | Zawadzki et al. (2022) | [34] | Reported statistical analyses |

| | | | |
|---|---|---|---|
| 35. | Almeida et al. (2022) | [35] | Wrong study design |
| 36. | Alzueta et al. (2022) | [36] | Reported statistical analyses |
| 37. | Andel et al. (2022) | [37] | Wrong study design |
| 38. | S. Bai et al. (2022) | [38] | Reported statistical analyses |
| 39. | Y. Bai et al. (2022) | [39] | Reported statistical analyses |
| 40. | Balouch et al. (2022) | [40] | Wrong study outcomes |
| 41. | Blok et al. (2022) | [41] | Wrong study design |
| 42. | Bonnar et al. (2022) | [42] | Wrong study outcomes |
| 43. | Burke et al. (2022) | [43] | Wrong population |
| 44. | Dominiak et al. (2022) | [44] | Reported statistical analyses |
| 45. | Goyer et al. (2022) | [45] | Wrong study design |
| 46. | Hamilton et al. (2021) | [46] | Wrong study design |
| 47. | Horwitz et al. (2022) | [47] | Reported statistical analyses |
| 48. | Kabir et al. (2022) | [48] | Wrong publication type |
| 49. | Kivela et al. (2022) | [49] | Wrong study outcomes |
| 50. | Krohn et al. (2022) | [50] | Reported statistical analyses |
| 51. | Le et al. (2022) | [51] | Wrong study design |
| 52. | Lewis et al. (2023) | [52] | Wrong study design |
| 53. | Manasse et al. (2022) | [53] | Reported statistical analyses |
| 54. | Massar et al. (2022) | [54] | Reported statistical analyses |
| 55. | Moilanen et al. (2020) | [55] | Wrong study outcomes |
| 56. | Newman et al. (2021) | [56] | Wrong study outcomes |
| 57. | Niemeijer et al. (2022) | [57] | Wrong study design |
| 58. | O’Rourke and Sixsmith (2021) | [58] | Wrong study design |
| 59. | Park et al. (2022) | [59] | Wrong study outcomes |
| 60. | Parsons and Young (2022) | [60] | Wrong study design |
| 61. | Riedy et al. (2022) | [61] | Reported statistical analyses |
| 62. | Şansal (2021) | [62] | Wrong study outcomes |
| 63. | Sletten et al. (2022) | [63] | Wrong study design |
| 64. | Wieman et al. (2022) | [64] | Wrong study design |
| 65. | Willroth et al. (2022) | [65] | Reported statistical analyses |
| 66. | Xia et al. (2022) | [66] | Wrong study outcomes |
| 67. | Yap et al. (2021) | [67] | Wrong study outcomes |
| 68. | Zhang et al. (2022) | [68] | Reported statistical analyses |
| 69. | Zubek et al. (2022) | [69] | Wrong study outcomes |
| 70. | Zuidersma et al. (2022) | [70] | Wrong study design |

| | | | |
|---|---|---|---|
| 71. | Amon et al. (2022) | [71] | Reported statistical analyses |
| 72. | Barbeau et al. (2022) | [72] | Wrong study design |
| 73. | Lim et al. (2022) | [73] | Wrong study outcomes |
| 74. | Walsh et al. (2022) | [74] | Wrong study outcomes |
| 75. | Asiamah-Asare et al. (2023) | [75] | Reported statistical analyses |
| 76. | Ballesio et al. (2023) | [76] | Reported statistical analyses |
| 77. | Bundo et al. (2023) | [77] | Reported statistical analyses |
| 78. | Frerart et al. (2023) | [78] | Reported statistical analyses |
| 79. | Junca-Silva and Mendes (2023) | [79] | Wrong study outcomes |
| 80. | Lee et al. (2023) | [80] | Reported statistical analyses |
| 81. | Massar et al. (2023) | [81] | Wrong study design |
| 82. | Mathew et al. (2023) | [82] | Multiple reports of the same sample |
| 83. | Menghini et al. (2023) | [83] | Reported statistical analyses |
| 84. | Nakagawa et al. (2023) | [84] | Reported statistical analyses |
| 85. | Newman et al. (2023) | [85] | Wrong study outcomes |
| 86. | Smith et al. (2023) | [86] | Reported statistical analyses |
| 87. | Speyer et al. (2023) | [87] | Wrong study design |
| 88. | Stapp et al. (2023) | [88] | Wrong study outcomes |
| 89. | Tang et al. (2023) | [89] | Reported statistical analyses |
| 90. | Urwin et al. (2023) | [90] | Reported statistical analyses |
| 91. | Walsh et al. (2023) | [91] | Reported statistical analyses |
| 92. | Whiston, Igou, Fortune, Analog Devices, et al. (2023) | [92] | Wrong study outcomes |
| 93. | Whiston, Igou, Fortune and Semkovska (2023) | [93] | Reported statistical analyses |
| 94. | Xu et al. (2023) | [94] | Reported statistical analyses |
| 95. | Zhang et al. (2023) | [95] | Reported statistical analyses |
| 96. | Zhu, Martin, et al. (2023) | [96] | Reported statistical analyses |
| 97. | Zhu, Zhao, et al. (2023) | [97] | Reported statistical analyses |
| 98. | Chen et al. (2024) | [98] | Reported statistical analyses |
| 99. | Dickman et al. (2024) | [99] | Wrong study outcomes |
| 100. | Hoyniak et al. (2024) | [100] | Wrong study design |
| 101. | Kinoshita et al. (2024) | [101] | Wrong study design |
| 102. | Labbaf et al. (2024) | [102] | Wrong publication type |
| 103. | Lenneis et al. (2024) | [103] | Multiple reports of the same sample |
| 104. | Li et al. (2024) | [104] | Reported statistical analyses |
| 105. | Palatine et al. (2024) | [105] | Wrong study design |
| 106. | Siepe et al. (2024) | [106] | Wrong study design |

| | | | |
|---|---|---|---|
| 107. | Song et al. (2024) | [107] | Wrong study design |
| 108. | Toenders et al. (2024) | [108] | Wrong study design |
| 109. | Wen et al. (2024) | [109] | Wrong study outcomes |
| 110. | Wijnhoven et al. (2024) | [110] | Wrong study outcomes |
| 111. | Xu et al. (2024) | [111] | Wrong study design |
| 112. | Yang et al. (2024) | [112] | Wrong study design |

**Table S4.** Data extraction categories.

| **Information extracted from full text records:** |
|---|
| • Author(s)<br>• Publication year<br>• Study location (geographical region)<br>• Study aims<br><br>• Assessment tools<br>• Sleep / affect parameters<br>• Actigraphic methods<br>• Number & timing of daily assessments<br>• Length of data collection<br><br>• Sample size & participant characteristics<br>• Inclusion / exclusion criteria<br>• Recruitment methods<br>• Main study conclusions<br>• Funding sources<br>• Potential conflicts of interest |

**Table S5.** Standardised affective state measures.

| **Daily mood or affect measure** | **Study incorporating measure, Author (Date)** |
|---|---|
| Affect Regulation Checklist (ARC) [113] | [114]<br><br>Adapted ARC items for daily emotion dysregulation |
| Altman Self-Rating Mania Scale (ASRM) [115] | [116,117]<br><br>For Tseng et al. (2022) mood scores were rated daily (0-6) and ASRM reported at weekly intervals |
| Beck Depression Inventory-II (BDI-II) [118] | [117] |
| Daily Life Questionnaire (DLQ). See [119] for measure details | [120] |
| Daytime Insomnia Symptom Scale (DISS) [3] | [121,122] |
| Depression, Anxiety, and Stress Scale (DASS-21) [123] | [116]<br><br>For Tseng et al. (2022) mood scores were rated daily (0-6) and DASS-21 reported at weekly intervals |
| Daily Inventory of Stressful Events (DISE) [124] | [125] |
| Modified Differential Emotions Scale (mDES) [126] | [127,128] |
| Mood and Anxiety Symptom Questionnaire (MASQ)<br><br>• Mini-MASQ [129]<br>• MASQ-SF [130] | [131,132] |
| Multidimensional Mood State Questionnaire (short version) (MDBF) [133] | [134] |
| Multi-Affect Indicator [135] | [136] |
| Non-Specific Psychological Distress Scale [137,138] | [139,140] |
| Positive and Negative Affect Schedule (PANAS) [141]<br><br>• PANAS-C (Positive and Negative Affect Schedule for Children) [142]<br>• PANAS-X (Positive and Negative Affect Schedule-Expanded Form) [143]<br>• PANAS-SF (Positive and Negative Affect Schedule-Short Form) [144] | 42 studies used the PANAS (includes studies with adapted or author selected PANAS items) [114,140,145-184]<br><br>• PANAS-C [147,173,184]<br>• PANAS-X [157,166,175,177,179]<br>• PANAS-SF [167,171,175] |
| Patient Health Questionnaire-4 (PHQ-4) [185] | [186,187] |
| Patient-Reported Outcomes Measurement Information System (PROMIS) [188] | [189] |
| Pleasantness Scale [190] | [191] |
| Profile of Mood States (POMS) [192]<br><br>• POMS-SF (Profile of Mood States-Short Form) [193] | The Profile of Mood States (POMS) items were used in 9 studies [175,194-201] (includes four studies with adapted or author selected POMS items)<br><br>One study [198] included the short form version (POMS-SF) |
| Russell's Circumplex Model of Affect [202] | [149,156,180] |
| Scale of Positive and Negative Experience (SPANE) [203] | [204] |

**Table S6.** Number and timing of self-report assessments.

| Author (Date) | Study length | Assessment tool | Timing of daily assessments | Self-reported ratings of sleep and affect |
|---|---|---|---|---|
| Totterdell et al. (1994) [205] | 14 days | Electronic pocket computer (Psion plc., London, UK) | Sleep diary (morning) & auditory reminder every 2 hours during waking (mood) | Separated ratings |
| Jones and Fletcher (1996) [206] | 21 days | Paper & pencil questionnaires | Completed daily measures each evening. Participants also contacted by telephone during the study | Same time ratings |
| Tzischinsky et al. (2001) [207] | 3 days (actigraphy for 5-7 days) | Paper & pencil questionnaires (with beeper) | Participants contacted 3 times via beepers at different times during the day | Separated ratings (Actigraphy only) |
| Zohar et al. (2005) [176] | 3 days (actigraphy for 5-7 days surrounding nightshifts, every 6 months. This covered the first 2 years of medical residency) | Paper & pencil questionnaires | Participants received 3 phone-call reminders at random times during their workday to complete study measures (asked to complete measures within 15 mins) | Separated ratings |
| Fuligni and Hardway (2006) [195] | 14 days | Paper & pencil diaries (with electronic time & date stamper) | Daily diaries completed at home every night before going to bed. Participants contacted by telephone once per week as a reminder to complete diaries | Same time ratings |
| McCrae et al. (2008) [160] | 14 days | Paper & pencil diaries | Completed daily measures every morning | Same time ratings |
| Galambos et al. (2009) [153] | 14 days | Online (web-based checklists) | Web-based checklists completed daily before going to sleep each night. The checklist was available from 6 p.m. until noon the next day | Same time ratings |
| Cousins et al. (2011) [147] | 8 days | Telephone interview (phone-call) | Participants received phone-call 12 times between 4 p.m. Friday and 10 p.m. Monday (24 calls in total over 2 weeks). EMA and sleep assessments collected during periods when participants were not in school | Separated ratings (Actigraphy only) |
| Gershon et al. (2012) [155] | 56 days | Paper & pencil diaries (time-stamped) | Sleep diary (morning) and affect diary (evening before bedtime). Participants called a voice mailbox after completion of each diary (morning and evening) to obtain a time-stamped record of completion | Separated ratings |
| Talbot et al. (2012) [198] | 7 days | Telephone (voicemail to record answer) | Sleep and mood measures completed via telephone at home by calling a voicemail box to record answers. Sleep diary and mood completed in morning (on waking) and a second mood response in evening | Same time ratings |

| | | | | |
|---|---|---|---|---|
| de Wild-Hartmann et al. (2013) [208] | 5 days | Paper & pencil questionnaires (digital wristwatch for ESM 'beep') | Sleep questionnaire completed on awakening (morning part) and prior to sleep (evening part). Positive and negative affect (ESM) rated 10 times each day via paper and pencil booklet. Digital wristwatch programmed to emit a signal ('beep') at an unpredictable moment in each of ten 90-mins time blocks between 07:30 and 22:30 every day | Separated ratings |
| Lev Ari and Shulman (2013) [196] | 7 days | Online questionnaires (website daily log) | Participants completed daily diary questionnaires every day through a secure online website (7 a.m. – Midnight) | Same time ratings |
| Ong et al. (2013) [125] | 7 days | Paper & pencil diaries and telephone interviews | Paper and pencil diaries completed daily. Daily positive affect reported through telephone interview each evening (10-15 mins) | Separated ratings |
| Doane and Thurston (2014) [151] | 4 days | Paper & pencil diaries | Paper and pencil diaries completed 5 times a day (15 diary entries in total). Completed on waking (morning), 30 mins after waking, 2 randomly selected times during the day and one entry before bed | Separated ratings |
| Garcia et al. (2014) [154] | 7 days | Mobile phone (SMS text message through 'YEMAS' Youth Ecological Momentary Assessment System) | SMS text of study questions 4 times each day (morning, after school, late afternoon, and evening; 28-44 questions per day). Each text was an individual question e.g. sleep quality, positive / negative mood | Separated ratings |
| Kalmbach et al. (2014) [157] | 14 days | Online questionnaires (web-delivered electronic questionnaires on home computer) | Electronic questionnaires completed daily on home computer each morning (upon waking) | Same time ratings |
| Takano et al. (2014) [172] | 7 days | Online questionnaire (via email link on mobile phone) | Participants received 8 daily emails on mobile phone between 8 a.m. and 12 a.m. Each email contained a link for the online questionnaire. Study days were divided into 8 intervals of 120 mins; e-mails were sent once for each interval with semi-random timing | Separated ratings (Actigraphy only) |
| Wrzus et al. (2014) [209] | 9 days | Online questionnaire (on Nokia E50 mobile phone) | Mobile phones (Nokia E50) were used to prompt participants 6 times a day to complete questionnaires; the 6 assessments occurred in 2-hour intervals with the exact time randomised. Participants chose between 6 a.m. and noon, when the first assessment (sleep duration) should occur | Separated ratings |
| Fortier et al. (2015) [152] | 14 days | Electronic questionnaire & 'Apple-application' developed for study (completed on personal electronic device or device supplied by researchers) | Questionnaires completed at the end of the day (interval-contingent) and after physical activity (event-contingent) on electronic device | Same time ratings |
| Li et al. (2015) [120] | 42 days | Smartphone app with EMA prompts | Smartphone app prompted questionnaire response at participants' preferred time each day (1 hour window allocated and 2-3 mins to complete questionnaire) | Same time ratings |
| Simor et al. (2015) [169] | 7 days | Paper & pencil questionnaires | Sleep quality reported in morning (upon waking) and 8 hours after awakening participants rated current affect | Separated ratings |
| van Zundert et al. (2015) [210] | 6 days | Smartphone app ('MyExperience') with signal prompt & reminders | Smartphones sent 'buzzing' signals 9 times a day at random time points during 90-mins intervals to complete questionnaires. Standard morning (sleep items) and evening questionnaires were also completed daily. Smartphones sent reminder signals every 2 mins until questionnaire was completed (maximum 3 reminders). After the third reminder (8 mins after first signal) the questionnaire was no longer available | Separated ratings |

| | | | | |
|---|---|---|---|---|
| Konen et al. (2016) [211] | 31 days | Smartphone app (with phone call prompt) | Affect was measured 4 times a day and sleep reported in the morning via smartphone app. Phones rang 4 times a day in school and at home to complete measures: 8:50 a.m. (Occasion 1), 11:25 a.m. (Occasion 2), 3 p.m. (Occasion 3), and 7 p.m. (Occasion 4) | Separated ratings |
| McCrae et al. (2016) [159] | 7 days | Paper & pencil diaries | Sleep diaries and affect completed once each morning | Same time ratings |
| Reis et al. (2016) [134] | 10 days (2 work weeks) | Smartphone app (movisensXS, Version 0.4.2453, movisens GmbH, Karlsruhe, Germany) with signal alarm prompts | Smartphone app prompted 3 'alarm' signals per day: in the morning before work (sleep quality), at noon (affect), and after work (affect) | Separated ratings |
| Tavernier et al. (2016) [212] | 3 days | Paper & pencil diaries | Morning diary (sleep) completed within 30 mins of waking and evening diary (daily experiences) before bedtime | Separated ratings (only reported actigraphic sleep outcomes in final analyses) |
| Blaxton et al. (2017) [145] | 56 days | Paper & pencil diaries | Daily sleep diary (upon waking) and affect measures (in evening) | Separated ratings |
| Bouwmans et al. (2017) [146] | 30 days | Electronic device & electronic diaries (PsyMate device; PsyMate BV, Maastricht, The Netherlands) | Sleep (morning) and affect 3 times a day using electronic diaries and PsyMate alarm (30 mins prior). Assessment times were fixed (adjusted to participant chronotype) with 6 hour intervals in between; on average timings were late morning (10 a.m.) afternoon (4 p.m.) and evening (10 p.m.) | Separated ratings |
| Chiang et al. (2017) [194] | 15 days (first 8 days with actigraphy) | Paper & pencil diaries (with security coded, pre-programmed time stampers & booklets) | Sleep diary (morning) and emotional experiences (evening prior to bed) with time-stamp recordings. Sleep self-reported for 8 days and affect across 15 days | Separated ratings (only reported actigraphic sleep outcomes in final analyses) |
| Flueckiger et al. (2017) [191] | Study 1 (33 days) & Study 2 (30 days; actigraphy subsample for 7 days) | Online surveys (via email link) | Online surveys sent at 5 p.m. via email (survey links accessible for the next 24 hours) | Same time ratings |
| Kalmbach et al. (2017) [131] | 14 days | Online questionnaire (web-delivered on home computer) | Daily online questionnaires (web-delivered) completed in morning (upon waking) | Same time ratings |
| Lauritsen et al. (2017) [213] | 28 days | Online surveys ('Daybuilder' webpage on personal computer or Mac) & SMS texts | Sleep recorded once and mood twice (morning and evening) each day. SMS texts sent as reminder to complete surveys on 'Daybuilder online webpage'. Mood values could also be entered through SMS | Separated ratings |
| McGrath et al. (2017) [161] | 5 days | Online surveys or paper & pencil | Daily surveys completed online or via paper and pencil. Positive affect & sleep quality assessed in morning | Same time ratings |
| Sin et al. (2017) [170] | 8 days | Telephone interview (phone call) | Telephone interviews (evening) for emotions that day and sleep on the prior night | Same time ratings |
| Cox et al. (2018) [214] | 7 days | Online surveys ('REDCap' Research Electronic Data Capture web-based platform via email links) | Sleep (morning) and anxiety 3 times each day in morning (8 a.m.) afternoon (2 p.m.) and evening (8 p.m.) via email link. Surveys completed within 2 hours of receipt on secure web-based platform (REDCap) | Separated ratings |
| da Estrela et al. (2018) [148] | 6 days | Electronic daily diaries (one telephone interview on the first day) | Daily electronic diaries completed at same time each evening. On the first day, the daily diary was a semi structured interview over the phone | Same time ratings |

| | | | | |
|---|---|---|---|---|
| Kalmbach et al. (2018) [215] | 2 months (pre-internship) & 6 months (during internship year) | Mobile phone SMS text & web-based mood-monitoring platform (Mood 247.com; Remedy Health Media LLC, New York, NY) | Daily automated SMS text (evening) at 8 p.m. to record daily mood ratings | Separated ratings (Actigraphy only) |
| Slavish et al. (2018) [197] | 14 days | Smartphone surveys (customised interface) | Daily sleep survey (morning) upon waking and emotions experienced that day (evening) within 1 hour before bedtime | Separated ratings |
| George et al. (2019) [114] | Study 1 (10 days) & Study 2 (14 days) | Smartphone app ('MetricWire') with EMA notification prompts | Affect reported in each survey signal across both studies. In study 1, participants completed 5 surveys every 4 hours; one in the morning (7 a.m. sleep & affect items), three during the daytime (affect items at 11 a.m., 3 p.m., & 7 p.m.), and one in the evening (affect at 11 p.m.). In study 2, participants completed 3 daily surveys: morning (sleep & affect), afternoon (affect), and evening (affect) | Separated ratings |
| Leger et al. (2019) [158] | 6 days | Mobile phone device to complete EMA surveys (Android device provided by the DEWS study 'Daily Experiences and Well-being Study') | Sleep (morning) reported upon waking and mood every 3 hours during waking hours (roughly the same time each day; an average of 20 within-day surveys) | Separated ratings |
| Li et al. (2019) [216] | 14 days | Smartphone (Motorola Droid RazrM) with EMA prompts | Once-daily sleep (morning) and twice-daily mood (morning & evening) surveys. Mood survey auto-generated by smartphone at random times (4-hour time windows) twice per day. Participants could choose schedule for the 4-hour window based on their sleep habits | Separated ratings |
| Merikangas et al. (2019) [217] | 14 days | Electronic personal digital device (Tungsten E2 PDA; Palm) & mobile app (Android platform) | Participants completed EMAs 4 times per day (fixed), with an approximately 4-hour delay between assessments. Initial study completed on personal digital assistant but since 2013 EMA was delivered using an Android mobile app | Separated ratings (Actigraphy only) |
| Parsey and Schmitter-Edgecombe (2019) [218] | 7 days | Mobile phone (phone-based self-report questions & automated call system) | Phone-based EMA completed 4 times each day using numeric phone keypad. Participants received an automated phone call to complete surveys at a random time within 4 time blocks: morning (9:30-11:30 a.m.), midday (12:30-2:30 p.m.), afternoon (3:30-5:30 p.m.), and evening (6:30-8:30 p.m.). System re-dialled 10 mins later if no response (2 redials within each block) | Separated ratings (Actigraphy only) |
| Triantafillou et al. (2019) [219] | 42 days | Smartphone app (Android app, Purple Robot, CBITs TECH Website, 2015) | Participants responded to sleep and affect surveys in morning (9 a.m.). Mood recorded 2 more times at 3 p.m. & 9 p.m. Responses could be delayed until later and survey times adjusted within a 2-hour window | Separated ratings |
| Williamson et al. (2018) [136] | 10 days (2 work weeks) | Smartphone app ('MetricWire') with EMA notification prompts | Signal-contingent smartphone ESM surveys completed mid-morning (sleep & mood) during workday | Same time ratings |
| Das-Friebel et al. (2020) [149] | 14 days | Smartphone app ('mEMA' Mobile Ecological Momentary Sampling) with EMA prompt notifications | Smartphone prompts 6 times each day to record sleep (every morning at 8 a.m.) and affect (remaining 5 notifications). Affect items randomly sent between 8 a.m. and 10 p.m. (on weekdays) or 10 a.m. and 10 p.m. (on weekends) with at least 1 hour between prompts. Each survey had a 20 mins time window | Separated ratings |
| O'Neill et al. (2020) [177] | 32 days | Online surveys (via email link) | Survey links emailed to participants once daily and were required to be completed between 5 p.m. and 11 p.m. Shift workers or participants with irregular hours completed surveys during a 5 a.m. to 11 a.m. time window | Same time ratings |

| | | | | |
|---|---|---|---|---|
| Peltz et al. (2019) [187] | 7 days | Online survey platform (time-stamp diary entries) | Morning diary (sleep duration & quality) completed within 1 hour of waking and evening diary (mood items; depressive & anxiety symptoms) completed within 1 hour of going to sleep | Separated ratings |
| Ryuno et al. (2021) [164] | 56 days | Paper & pencil questionnaires | Affect surveys completed each evening (before sleep) | Separated ratings (Actigraphy only) |
| Sayre et al. (2021) [165] | 8 days | Telephone interview (phone call) | Phone-call interview (evening) at 7 p.m. (only workdays were analysed for the study) | Same time ratings |
| Sin et al. (2020) [220] | 8 days | Telephone interview (daily phone call with computer-assisted telephone interview programming) | Daily interviews by telephone (evening) | Same time ratings |
| Wen et al. (2020) [221] | 7 days | Mobile phone (WeChat platform) & online survey link | Affect items recorded 5 times each day with surveys sent via WeChat platform. Participants had 1 hour to complete each online survey link | Separated ratings (Actigraphy only) |
| Bean and Ciesla (2021) [132] | 14 days | Online questionnaires (completed on smartphone) | Online questionnaires completed on smartphone twice per day. Surveys sent in morning (6 a.m.) completed upon waking and in the evening (8 p.m.) completed 2 hours before bedtime | Separated ratings |
| Díaz-Morales and Parra-Robledo (2021) [150] | 7 days | Paper & pencil diaries (with email reminders) | Sleep and mood diaries completed in evening before sleep with email reminders | Same time ratings |
| Difrancesco et al. (2021) [222] | 14 days | Smartphone electronic diary | Smartphone EMA items sent 5 times each day (every 3 hours). Sleep reported in first assessment of the day | Separated ratings |
| Fang et al. (2021) [223] | 2 months (prior to medical internship) & 1 year (during internship). Actigraphy (minimum of 7 days) | Smartphone app ('Intern+' developed by Remedy Health Media LLC, New York) with push notifications | Daily push notifications from smartphone app to assess mood at user-specified time (between 5 - 10 p.m.) | Separated ratings (Actigraphy only) |
| Jones et al. (2022) [156] | 7 days | Online surveys ('Survey Signal' platform sent Qualtrics link via SMS / text messages to phone) | Survey links (via SMS text) sent 8 times each day: survey upon waking (sleep & affect items sent at 6 a.m. and available until 11 a.m.), evening survey prior to bed (health behaviour items sent at 8:30 p.m. and available until 2 a.m.) and 6 ambulatory surveys (affect items) sent at semi-random 2 hour intervals. No reminder messages for morning & evening surveys but the 6 ambulatory surveys had reminder texts after 25 mins with links expiring after 30 mins | Separated ratings |
| Lee (2022) [224] | 8 days | Telephone interview (phone call) | Diary phone interviews at end of day (sleep & well-being measures) | Same time ratings |
| Messman et al. (2021) [162] | 7 days | Online surveys ('REDCap' Research Electronic Data Capture web-based platform via email links) | Survey link for sleep & affect diaries (via email in morning) with 2 reminders sent at 3 hour intervals if incomplete. Participants messaged by researchers again if diaries not completed by noon | Same time ratings |
| Minaeva et al. (2021) [225] | 5 days | Paper & pencil questionnaires (digital wristwatch for ESM 'beep') | Digital wristwatch signal ('beep') to record affect at random times within ten 90-mins time blocks (between 7:30 a.m. and 10:30 p.m.). Sleep diary (morning) | Separated ratings |

| | | | | |
|---|---|---|---|---|
| Neubauer et al. (2021) [226] | Study 1 (28 days) & Study 2 (21 days) | Smartphone surveys with prompts | Study 1: smartphone survey prompts 4 times each day (fixed times). Morning time window (6–7:50 a.m.), late mornings (9:50 a.m.), afternoons (3 p.m. – 5:15 p.m. or 4 p.m. –5:45 p.m. on longer school days), and evenings (6:30–8:30 p.m.). Sleep items completed in morning surveys and affect items in all 4 survey prompts.<br><br>Study 2: smartphone survey prompts 3 times each day outside of school context. Morning surveys scheduled during week (at 6:45 a.m. to 7:50 a.m.) and weekends (at 6:45 a.m. to 9:15 a.m.), afternoon and evening survey timings could be chosen by parent with an earlier option (3 p.m. to 5:15 p.m. and 6 p.m. to 8:30 p.m.) or later option (4 p.m. to 6:15 p.m. and 7 p.m. to 9:30 p.m.). Sleep items completed in morning surveys and affect items in all 3 survey prompts | Separated ratings |
| Shen et al. (2022) [166] | 28 days | Smartphone app ('MetricWire') with EMA notification prompts | Sleep reported each morning (7 a.m. to 2:30 p.m.) and affect each afternoon (3:30 p.m. to 7 p.m.) via smartphone app | Separated ratings |
| Shi et al. (2021) [168] | 7 days | Mobile phone (WeChat platform) & online survey link | Affect recorded 5 times each day (9 a.m. to 8 p.m.) at random intervals of 120–240 mins via online survey link / WeChat (1 hour to complete each survey) | Separated ratings (Actigraphy only) |
| Simor et al. (2021) [227] | 14 days | Online questionnaires & email reminders | Sleep items upon waking (questionnaire available from 5 a.m. to 12:30 p.m.) and mood measures before bedtime (available from 6 p.m. to 3 a.m.). Reminder emails also sent | Separated ratings |
| Sin et al. (2021) [228] | 8 days | Telephone interview (phone call) | Diary phone interviews at end of day (daily sleep & well-being measures) | Same time ratings |
| Sun-Suslow et al. (2021) [229] | 14 days | Smartphone EMA prompts (Samsung Galaxy with 4G Android Operating System) | Smartphone EMA surveys (4 each day) randomised at 3 hour time intervals and adjusted to participant sleep-wake schedules. Sleep items in first EMA survey (morning) and mood reported at each prompt | Separated ratings |
| Vigoureux and Lee (2021) [230] | 14 days | Smartphone app (RealLife Exp, LifeData Corporation) with notifications | Smartphone surveys 4 times per day (upon waking, before lunch, afternoon & before bedtime). Morning sleep survey was event-based (user initiated waking survey) and remaining 3 surveys were notification-initiated (time-based) to report momentary sleepiness & affect<br><br>For day shift nurses, notifications were scheduled for 11 a.m., 3 p.m., and 9 p.m. For night shift nurses, notifications were scheduled for 11 p.m., 3 a.m., and 9 a.m. Surveys expired after 1 hour | Separated ratings |
| Wang et al. (2021) [174] | 7 days | Online survey (via email & optional SMS text reminder) | Online survey sent in morning (8 a.m. for sleep & affect items) and in evening (8 p.m. for affect) | Separated ratings |
| Wieman et al. (2022) [231] | 7 days | Online surveys ('Qualtrics' sent via SMS text) | Survey links SMS texted 3 times each day (at 8 a.m., 2 p.m. and 8.pm). Sleep items completed in morning survey and affect at each EMA time point | Separated ratings |
| Wong et al. (2021) [175] | 4 days (actigraphy for 7 days) | Electronic personal digital device (PDA; Palm Z22, software: Satellite Forms) | Personal digital device (PDA) prompted participants to complete affect items every 1 hour throughout the day. PDA also used to indicate when participant awoke. During EMA study participants telephoned 4 times for technical support | Separated ratings |

| | | | | |
|---|---|---|---|---|
| Chan et al. (2022) [232] | 30 days | Online diary | Daily diaries sent to participants at 9 a.m. (sleep & anxiety items) and 11 p.m. each day (anxiety items) | Separated ratings |
| Dickens et al. (2021) [204] | 28 days | Online surveys ('Survey Signal' platform sent Qualtrics link via SMS / text messages to phone) | Survey links sent in morning for sleep (upon waking) and end of the day for affect items (evening) | Separated ratings |
| Liu et al. (2022) [139] | 8 days | Telephone interview (phone call) & sleep diary | Daily evening phone calls to report on affect and completed daily sleep diary | Separated ratings |
| Narmandakh et al. (2021) [233] | 30 days | Online questionnaire (SMS text link to online platform used in Dutch study, "How Nuts Are The Dutch") | SMS text sent 3 times each day (with 6 hour intervals) for online questionnaires. Text time points were adjusted for each participant sleep-wake schedule and questionnaires available for 1 hour. Affect was measured 3 times at each EMA prompt and sleep reported in morning | Separated ratings |
| Parsons et al. (2022) [234] | 7 days | Smartphone app ('MetricWire') with EMA notification prompts | Smartphone notifications sent 4 times each day. First survey sent at random timepoint between 10 a.m. and 12 p.m. (included sleep and emotion regulation questions). Remaining 3 surveys sent at pseudo-random times (at least 2 hours apart) between 1 p.m. and 10 p.m. (included emotion measures). Links to surveys expired after 20 mins | Separated ratings |
| Song et al. (2021) [171] | 10 days (2 work weeks) | Mobile phone (WeChat platform) & online survey link | Online daily survey link sent in morning (9 a.m. for sleep items) and evening (8:30 p.m. for affect) on WeChat. 1 hour window to complete each survey with reminders sent after 30 mins if incomplete | Separated ratings |
| Sperry & Kwapil (2022) [235] | 14 days | Smartphone app with ESM notifications | ESM affect survey sent 8 times each day at random times between 10 a.m. to 10 p.m. (within 90 mins stratified intervals). Survey notifications completed within 10 mins. Sleep diaries sent at 5 a.m. and available for completion until the end of that day | Separated ratings |
| Ying et al. (2021) [236] | 7 days | Online surveys ('Qualtrics' link via email) | Morning survey link (sleep items) emailed at 7 a.m. and evening surveys (current-day affect) emailed at 7 p.m. | Separated ratings |
| Harris et al. (2022) [127] | 7 days | Online diaries (via email link) | Online sleep diary emailed in morning (4 a.m.) and completed upon waking with reminder (1 p.m.). Affect survey in evening (6 p.m.) and completed before bed with reminder email (1 a.m.) | Separated ratings |
| Hruska et al. (2022) [189] | 8 days | Online surveys ('REDCap' Research Electronic Data Capture web-based platform via email links) | Daily surveys (sleep and anger emotions) emailed at 6 a.m. each day. Participants (emergency medical workers) completed surveys upon receipt if they had already worked or if they were not working that day. If working later in the day participants completed surveys after shift | Same time ratings |
| Lee et al. (2023) [237] | 10 days | Online surveys (via SMS text completed on smartphone) | SMS text messages sent with an online survey link. Sleep disturbance reported in morning (9 a.m.) and mood / sleepiness 4 times each day (9 a.m., 1 p.m., 5 p.m., 9 p.m.) | Separated ratings |
| Marcusson-Clavertz et al. (2022) [238] | • ESCAPE (14 days)<br>• SHADE (7 days)<br>• SAWM (7 days) | • ESCAPE study - smartphone with ESM 'beep'<br>• SHADE study - palmtop computer<br>• SAWM Study - palmtop computer | • ESCAPE – morning sleep survey (self-initiated upon waking) and 5 random ESM surveys (affect items) each day. Smartphone 'beep' at semi-random times (interval-based) adjusted to participant's typical waking time<br>• SHADE – morning sleep survey (upon waking) and 5 random surveys (affect items) each day at roughly 3-hour intervals<br>• SAWM – morning sleep survey (upon waking) and 5 random surveys (affect items) each day based on participants' self-reported wake time | Separated ratings |

| | | | | |
|---|---|---|---|---|
| Mousavi et al. (2022) [163] | 3 months | Smartphone app (Android EMA app) with reminders sent via SMS text, email, or phone call | Daily affect (evening) via EMA smartphone app | Separated ratings (Actigraphy only) |
| Newman et al. (2022) [128] | 21 days total (1 week of assessments at 3 study time points) | Paper & pencil and email diaries | Morning questionnaires completed on paper (sleep diary & affect) and evening diary (affect) sent by email at participants' preferred time (around 8 p.m.) | Separated ratings |
| Patapoff et al. (2022) [239] | 11 days (actigraphy for 14 days)<br><br>[sample included individuals with both actigraphy and EMA data for 2–11 days. Actigraphy device worn for 14 day period in annual assessments; some had data from multiple (2–4) years] | Smartphone EMA and sleep diary | EMA mood surveys sent 3 times each day (morning, afternoon, evening – surveys sent sporadically in intervals specified by participant). Sleep quality measured daily as part of the EMA survey and participants also completed a sleep diary | Separated ratings |
| Peltz and Rogge (2022) [186] | 7 days | Online survey (via email) | Sleep quality (morning diaries) and mood (evening diaries) sent via email each day | Separated ratings |
| Roberts et al. (2022) [240] | 14 days | Online diary (via email links on Google forms) | Morning diary upon waking (completed within 1 hour or so) and evening diary (within 1 hour or so before bed) assessed sleep and mood. Email reminders if missing diary entries | Separated ratings |
| Shi and Wang (2022) [167] | 10 days | Online survey (WenJuanXing platform) | Daily diaries twice each day on workdays with reminders sent by researchers. Morning diary upon waking (sleep & affect prior to work) and diary at end of workday (affect) | Separated ratings |
| Titone et al. (2022) [117] | 20 days | Mobile phone SMS text | Mood measured 3 times daily (morning, afternoon, evening) via text message | Separated ratings (Actigraphy only) |
| Tseng et al. (2022) [116] | 2 – 398 days (average of 3 months) | Smartphone app (also collected active & passive data e.g. GPS data as indicator of movement activity) | Daily sleep and mood recorded in smartphone app (no information on number and timing) | Separated ratings |
| Wang et al. (2023) [173] | 29 days | Daily diary on 'internet-capable devices' (reminders via email or SMS) | Daily diary (sleep & affect) completed between 5 p.m. and midnight with 2-4 reminders each day via email or SMS | Same time ratings |
| Yip et al. (2022) [199] | 14 days | Electronic tablet for daily diary | Daily diary (sleep & mood) completed in evening before bed | Same time ratings |
| Kouros et al. (2022) [241] | 7 days | Paper & pencil questionnaires | Daily diary (sleep & mood) completed in evening | Same time ratings |
| Lucke et al. (2022) [242] | 7 days | Electronic tablet (Apple iPad) and paper & pencil questionnaire versions (for a small number of participants). Phone call to clarify any questions on second day | Morning survey upon waking (self-initiated event-based; for affect & sleep quality). Momentary affect every 2-3 hours (at 10 a.m.,1 p.m., 4 p.m., 7 p.m., & 9 p.m.) prompted by iPad (leeway of 30 min before and 2 hr after prompted times) | Separated ratings & same time*<br><br>*morning survey had affect & sleep quality* |
| Sheppard et al. (2022) [183] | 10 days*<br><br>*workdays over 2 consecutive work weeks* | ESM tool (digital but not specified) | Daily surveys at noon (sleep & affect) sent at 11 a.m. (available until 2 p.m.) | Same time ratings |
| Barber et al. (2023) [140] | 8 days | Telephone interview (phone call) | Telephone interview in evening (affect & sleep) | Same time ratings |

| | | | | |
|---|---|---|---|---|
| Chachos et al. (2023) [179] | 7-28 days*<br><br>**STEPS (28 days), ACES (12 days), DESTRESS (7 days)* | Smartphone app (‘MetricWire’) with EMA notification prompts | STEPS: sleep survey available (7 a.m. to 2:30 p.m.) & affect (3:30 p.m. to 7 p.m.)<br><br>ACES / DESTRESS: sleep survey available (11 a.m. to 3 p.m.) & affect three times daily from 11 a.m. to 3 p.m. (morning), 3.30 p.m. to 7:30 p.m. (afternoon) & 8 p.m. to 02:00 a.m. (evening)<br><br>**only afternoon survey analysed* | Separated ratings |
| Hachenberger et al. (2023) [180] | 14 days | Smartphone app (‘movisensXS’, Version 1.5.13) | Short surveys (2–3 mins) sent 7 times per day. First survey prompt (affect & sleep) sent (8-9 a.m. on weekdays or 9-10 a.m. on weekends). Next 5 survey prompts (affect over past 1.5 hrs) sent (10:30 a.m. to 7:30 p.m. on weekdays & 11:30 a.m. to 7:30 p.m. on weekends). Last survey prompt at end of day (affect) sent (9 -10 p.m.). Prompts sent at random times within intervals. Gap of at least 90 mins between two adjacent prompts. Surveys available for 30 mins | Separated ratings & same time*<br><br>**morning survey had affect & sleep* |
| Jordan et al. (2023) [243] | 14 days | Electronic diaries (REDCap) | Sleep diary & mood (morning) upon waking | Same time ratings |
| Kirshenbaum et al. (2023) [244] | 3-14 days*<br><br>**at least 3 days of usable actigraphy data* | Smartphone app (‘MetricWire’) with EMA notification prompts | Morning (sleep & affect) prompt, afternoon (affect) & night (affect) | Separated ratings & same time*<br><br>**morning survey had affect & sleep* |
| Master et al. (2023) [200] | 7 days*<br><br>**at least 3 valid actigraphy days* | Electronic diaries (via authenticated web-link) | Electronic diary (evening) after 7 p.m. (before going to sleep) | Separated ratings (Actigraphy only) |
| McGowan et al. (2023) [201] | 28 days | Smartphone app (‘LifeData, RealLife Exp’) | Morning sleep survey (8 a.m.) & evening survey (6 p.m.) with prompt reminders. Mood / emotions twice a day | Separated ratings |
| Ng et al. (2023) [245] | 28 days (Study 1) & 14 days (Study 2) | Smartphone app (‘Z4IP EMA’) | EMA twice daily (mood & sleepiness) – during post-wake window (8 a.m. to 1:59 p.m.) & pre-bedtime window (8 p.m. to 23:59 p.m.) | Separated ratings (Actigraphy only) |
| Ohana and Fortin (2023) [181] | 8-10 days | Online (web-based Qualtrics survey) | Daily diary questionnaire (end of each working day) | Same time ratings |
| Punna et al. (2023) [246] | 7 days | Mobile phone (with web-based application adapted for mobile device) | Timing varied (between 1 p.m. and 5 p.m.) with 4 hours to answer & reminder sent | Separated ratings |
| Rea et al. (2023) [182] | 7 days | Online (emailed via SurveyMonkey) | Survey links emailed at 8 p.m. in evening and available until 12 p.m. next day. Reminder emails sent at 11 p.m. | Same time ratings |
| Sell et al. (2023) [247] | 14 days | Online (emailed surveys) | Study 1 - Emailed questionnaires at 6 p.m. (available until midnight to complete) for affect that day & sleep<br><br>Study 2 - Emailed questionnaires at 4 p.m. (available until midnight to complete) for affect & sleep | Same time ratings |
| Song et al. (2023) [248] | 28-55 days*<br><br>**average days 33.69 (4.17)* | Mobile phone (SMS text message) | SMS text to complete survey questions 4 times per day (randomised schedule every 4 hours). First survey for sleep | Separated ratings |
| Xie et al. (2023) [249] | 7 days | Daily diary (unclear if electronic) | Affect & sleep (once a day) | Not specified |

| | | | | |
|---|---|---|---|---|
| Baglioni et al. (2024) [178] | 5 days | Smartphone app ('movisensXS') | Sleep diaries (morning within 30 mins of waking & evening before bed). Affect in morning, evening and 4 random times during day | Separated ratings & same time*<br><br>*morning survey had affect & sleep* |
| Collier Villaume et al. (2024) [250] | 3 days | Diaries (not specified if digital) | EMA (affect) recorded 6 times daily | Separated ratings (Actigraphy) |
| Dong et al. (2024) [251] | 7 days | Electronic diaries (with prompt) | Participants prompted to fill out the daily diary / surveys within 24 hours. Mood upon awakening | Separated ratings (Actigraphy) |
| Evans et al. (2024) [252] | 9 days | Smartphone app (study smartphone with custom EMA app) | Weekend surveys (8 PA/NA prompts at quasi-random intervals between 10 a.m. to 10 p.m.). Weekdays (4 PA/NA prompts, including one in morning & 3 at quasi-random intervals between 4p.m. to 9:30 p.m.). Sleep diary was user-initiated | Separated ratings |
| Kwan et al. (2024) [253] | 14 days*<br><br>**10-11 weekdays* | Online (web-based daily survey) | Morning survey (9 a.m.) each weekday (sleep & affect). Afternoon survey (4 p.m.) each weekday (affect). Reminder after 30 mins | Separated ratings & same time*<br><br>**morning survey had affect & sleep* |
| Lee et al. (2024) [254] | 8 days | Telephone interview (phone-call) | Phone-call in evening for daily experiences | Same time ratings |
| Meigs et al. (2024) [255] | 7 days | Smartphone (via 'ReTAINE' platform) | Surveys sent 3 times a day: morning (sleep & mood) afternoon (mood), evening (mood) | Separated ratings & same time*<br><br>**morning survey had mood & sleep* |
| Peng et al. (2024) [256] | 27-50 days*<br><br>**average 47.66 days (SD = 4.28)* | Online surveys (via Wenjuanxing platform) | Surveys sent at 8 p.m. (deadline to respond by 3 a.m. next day) | Same time ratings |
| Poon et al. (2024) [122] | 7 days | Smartphone app (in-house app, 'Longitudinax' version 1.4.2) | Push notifications (mood) from app at 4 time-points (9 a.m., 1 p.m., 5 p.m., & 9 p.m.). Reminder prompt 5 mins later (up to two reminders within 15 mins each time) | Separated ratings (Actigraphy only) |
| Wescott et al. (2024) [257] | 3–17 days | Electronic diaries | Morning diary (sleep & affect) | Same time ratings |
| Xie et al. (2024) [184] | 7 days | Diaries (not specified if digital) | Affect in morning (6:30 a.m.) & evening (8:30 p.m.). Sleep in morning upon waking | Separated ratings & same time*<br><br>**morning survey had affect & sleep* |
| Zapalac et al. (2024) [258] | 35 days*<br><br>**average 35.20 (SD = 27.01) days of observation per participant* | Smartphone app ('Beiwe™ smartphone app' & research platform) | EMAs twice per day – morning (sleep & mood) at 9.a.m. & evening (mood) at 7 p.m. EMAs distributed on Monday, Wednesday, Friday, and Sunday | Separated ratings & same time*<br><br>**morning survey had mood & sleep* |

## References


1. Berry, D.T.; Webb, W.B. Mood and sleep in aging women. *Journal of Personality and Social Psychology* **1985**, *49*, 1724-1727, doi:10.1037//0022-3514.49.6.1724.
2. Bower, B.; Bylsma, L.M.; Morris, B.H.; Rottenberg, J. Poor reported sleep quality predicts low positive affect in daily life among healthy and mood-disordered persons. *Journal of Sleep Research* **2010**, *19*, 323-332, doi:10.1111/j.1365-2869.2009.00816.x.
3. Buysse, D.J.; Thompson, W.; Scott, J.; Franzen, P.L.; Germain, A.; Hall, M.; Moul, D.E.; Nofzinger, E.A.; Kupfer, D.J. Daytime symptoms in primary insomnia: a prospective analysis using ecological momentary assessment. *Sleep Medicine* **2007**, *8*, 198-208, doi:10.1016/j.sleep.2006.10.006.
4. Casement, M.D.; Livingston, N.R.; Allen, N.B.; Forbes, E.E. Feasibility, acceptability and affective consequences of at-home sleep extension in young women with depressive symptoms: A pilot study. *Journal of Sleep Research* **2021**, *30*, e13045, doi:10.1111/jsr.13045.
5. Elovainio, M.; Kuula, L.; Halonen, R.; Pesonen, A.K. Dynamic fluctuations of emotional states in adolescents with delayed sleep phase-A longitudinal network modeling approach. *Journal of Affective Disorders* **2020**, *276*, 467-475, doi:10.1016/j.jad.2020.07.050.
6. Kammerer, M.K.; Mehl, S.; Ludwig, L.; Lincoln, T.M. Sleep and circadian rhythm disruption predict persecutory symptom severity in day-to-day life: A combined actigraphy and experience sampling study. *Journal of Abnormal Psychology* **2021**, *130*, 78-88, doi:10.1037/abn0000645.
7. Kaufmann, C.N.; Gershon, A.; Eyler, L.T.; Depp, C.A. Clinical significance of mobile health assessed sleep duration and variability in bipolar disorder. *Journal of Psychiatric Research* **2016**, *81*, 152-159, doi:10.1016/j.jpsychires.2016.07.008.
8. Kouros, C.D.; El-Sheikh, M. Daily mood and sleep: reciprocal relations and links with adjustment problems. *Journal of Sleep Research* **2015**, *24*, 24-31, doi:10.1111/jsr.12226.
9. Lemola, S.; Ledermann, T.; Friedman, E.M. Variability of sleep duration is related to subjective sleep quality and subjective well-being: an actigraphy study. *PLoS One* **2013**, *8*, e71292, doi:10.1371/journal.pone.0071292.
10. O'Leary, K.; Small, B.J.; Panaite, V.; Bylsma, L.M.; Rottenberg, J. Sleep quality in healthy and mood-disordered persons predicts daily life emotional reactivity. *Cognition and Emotion* **2016**, *31*, 435-443, doi:10.1080/02699931.2015.1126554.
11. Peltz, J.S. The day-to-day impact of nighttime noise disturbances on college students' psychological functioning. *Journal of American College Health* **2022**, *70*, 2061-2069, doi:10.1080/07448481.2020.1842422.
12. Pressman, S.D.; Jenkins, B.N.; Kraft-Feil, T.L.; Rasmussen, H.; Scheier, M.F. The whole is not the sum of its parts: Specific types of positive affect influence sleep differentially. *Emotion* **2017**, *17*, 778-793, doi:10.1037/emo0000256.
13. Strahler, J.; Nater, U.M.; Skoluda, N. Associations between Health Behaviors and Factors on Markers of Healthy Psychological and Physiological Functioning: a Daily Diary Study. *Annals of Behavioral Medicine* **2020**, *54*, 22-35, doi:10.1093/abm/kaz018.

14. Thota, D. Evaluating the Relationship Between Fitbit Sleep Data and Self-Reported Mood, Sleep, and Environmental Contextual Factors in Healthy Adults: Pilot Observational Cohort Study. *JMIR Formative Research* **2020**, *4*, e18086, doi:10.2196/18086.
15. Totterdell, P.; Spelten, E.; Barton, J.; Smith, L.; Folkard, S. On-shift and daily variations in self-report and performance measures in rotating-shift and permanent night nurses. *Work & Stress* **1995**, *9*, 187-197, doi:10.1080/02678379508256553.
16. Coifman, K.G.; Seah, T.H.S.; Nylocks, K.M.; Wise, A.; Almahmoud, S.; Summers, C.; Aurora, P.; Garcia, M.; Delahanty, D.L. Micro Versus Macro Processes: How specific stress exposure impacts sleep, affect, and risk-related behavior on the path to disease in high-risk adults. *Anxiety Stress Coping* **2021**, *34*, 381-396, doi:10.1080/10615806.2021.1888933.
17. Dong, Y.; Zhang, X. Study on the effect of awakening daylight in dormitories on morning alertness, mood, fatigue and sleep quality of college students. *Building and Environment* **2021**, *203*, 108060, doi:10.1016/j.buildenv.2021.108060.
18. Gumport, N.B.; Gasperetti, C.E.; Silk, J.S.; Harvey, A.G. The Impact of Television, Electronic Games, and Social Technology Use on Sleep and Health in Adolescents with an Evening Circadian Preference. *Journal of Youth and Adolescence* **2021**, *50*, 2351-2362, doi:10.1007/s10964-021-01429-9.
19. Hussain, M.; Kho, C.; Main, A.; Zawadzki, M.J. Horizontal Collectivism Moderates the Relationship Between in-the-Moment Social Connections and Well-Being Among Latino/a College Students. *Journal of Immigrant and Minority Health* **2021**, *23*, 1001-1010, doi:10.1007/s10903-021-01143-5.
20. Hyde, J.; Eidels, A.; van Amelsvoort, T.; Myin-Germeys, I.; Campbell, L. Gene Deletion and Sleep Depletion: Exploring the Relationship Between Sleep and Affect in 22q11.2 Deletion Syndrome. *The Journal of Genetic Psychology* **2021**, *182*, 304-316, doi:10.1080/00221325.2021.1930995.
21. Kaurin, A.; Hisler, G.; Dombrovski, A.Y.; Hallquist, M.N.; Wright, A.G.C. Sleep and next-day negative affect and suicidal ideation in borderline personality disorder. *Personality Disorders: Theory, Research, and Treatment* **2022**, *13*, 160-170, doi:10.1037/per0000496.
22. Lee, S.; Bonnar, D.; Kim, Y.; Lee, Y.; Lee, S.; Gradisar, M.; Suh, S. Sleep Characteristics and Risk Factors of Korean Esports Athletes: An Exploratory Study. *Sleep Medicine Research* **2020**, *11*, 77-87, doi:10.17241/smr.2020.00773.
23. Moshe, I.; Terhorst, Y.; Opoku Asare, K.; Sander, L.B.; Ferreira, D.; Baumeister, H.; Mohr, D.C.; Pulkki-Råback, L. Predicting Symptoms of Depression and Anxiety Using Smartphone and Wearable Data. *Frontiers in Psychiatry* **2021**, *12*, 625247, doi:10.3389/fpsyt.2021.625247.
24. Muzni, K.; Groeger, J.A.; Dijk, D.J.; Lazar, A.S. Self-reported sleep quality is more closely associated with mental and physical health than chronotype and sleep duration in young adults: A multi-instrument analysis. *Journal of Sleep Research* **2020**, *30*, e13152, doi:10.1111/jsr.13152.
25. Philbrook, L.E.; Macdonald-Gagnon, G.E. Bidirectional Relations Between Sleep and Emotional Distress in College Students: Loneliness as a Moderator. *The Journal of Genetic Psychology* **2021**, *182*, 361-373, doi:10.1080/00221325.2021.1913982.
26. Mousa, S.; Latchford, G.; Weighall, A.; Nash, H.; Murray-Leslie, R.; Reuber, M.; Relton, S.D.; Graham, C.D. Evidence of objective sleep impairment in nonepileptic attack disorder: A naturalistic prospective controlled study using actigraphy and daily sleep diaries over six nights. *Epilepsy & Behavior* **2021**, *117*, 107867, doi:10.1016/j.yebeh.2021.107867.

27. Saksvik-Lehouillier, I.; Nordhaug, L.; Owesen, S.M.; Karlsen, H.R. The rhythm of affect, autonomy, competence and relatedness: A pilot diary study. *Chronobiology International* **2021**, *38*, 480-488, doi:10.1080/07420528.2020.1867156.
28. Sano, A.; Phillips, A.J.; McHill, A.W.; Taylor, S.; Barger, L.K.; Czeisler, C.A.; Picard, R.W. 0182 Influence of Weekly Sleep Regularity on Self-Reported Wellbeing. *Sleep* **2017**, *40*, A67-A68, doi:10.1093/sleepj/zsx050.181.
29. Seidl, E.; Venz, J.; Ollmann, T.M.; Voss, C.; Hoyer, J.; Pieper, L.; Beesdo-Baum, K. How current and past anxiety disorders affect daily life in adolescents and young adults from the general population-An epidemiological study with ecological momentary assessment. *Depression and Anxiety* **2021**, *38*, 272-285, doi:10.1002/da.23133.
30. Shen, L.; Wiley, J.F.; Bei, B. Perceived daily sleep need and sleep debt in adolescents: associations with daily affect over school and vacation periods. *Sleep* **2021**, *44*, doi:10.1093/sleep/zsab190.
31. Nater-Mewes, R.; Müller, L.R.F.; Gossmann, K.; Schmid, R.F.; Rosner, R.; Unterhitzenberger, J. A pilot study on ecological momentary assessment in asylum-seeking children and adolescents resettled to Germany: Investigating compliance, post-migration factors, and the relation between daily mood, sleep patterns, and mental health. *PLoS One* **2021**, *16*, e0246069, doi:10.1371/journal.pone.0246069.
32. Wu, C.; Fritz, H.; Bastami, S.; Maestre, J.P.; Thomaz, E.; Julien, C.; Castelli, D.M.; de Barbaro, K.; Bearman, S.K.; Harari, G.M.; et al. Multi-modal data collection for measuring health, behavior, and living environment of large-scale participant cohorts. *Gigascience* **2021**, *10*, doi:10.1093/gigascience/giab044.
33. Zamora, A.N.; Arboleda-Merino, L.; Tellez-Rojo, M.M.; O'Brien, L.M.; Torres-Olascoaga, L.A.; Peterson, K.E.; Banker, M.; Fossee, E.; Song, P.X.; Taylor, K.; et al. Sleep Difficulties among Mexican Adolescents: Subjective and Objective Assessments of Sleep. *Behavioral Sleep Medicine* **2022**, *20*, 269-289, doi:10.1080/15402002.2021.1916497.
34. Zawadzki, M.J.; Hussain, M.; Kho, C. Comparing multidimensional facets of stress with social, emotional, and physical well-being using ecological momentary assessment among a Hispanic sample. *Stress Health* **2022**, *38*, 375-387, doi:10.1002/smi.3098.
35. Almeida, F.; Pires, L.; Bettencourt, C.; Almeida, R.; Ruivo Marques, D.; Leitão, J.; Gomes, A.A. Momentary emotional states in primary school children: combined effects of chronotype X time-of-day. *Biological Rhythm Research* **2022**, *54*, 52-69, doi:10.1080/09291016.2022.2081400.
36. Alzueta, E.; de Zambotti, M.; Javitz, H.; Dulai, T.; Albinni, B.; Simon, K.C.; Sattari, N.; Zhang, J.; Shuster, A.; Mednick, S.C.; et al. Tracking Sleep, Temperature, Heart Rate, and Daily Symptoms Across the Menstrual Cycle with the Oura Ring in Healthy Women. *International Journal of Women's Health* **2022**, *14*, 491-503, doi:10.2147/IJWH.S341917.
37. Andel, S.A.; Pindek, S.; Spector, P.E.; Crowe, R.P.; Cash, R.E.; Panchal, A. Adding fuel to the fire: The exacerbating effects of calling intensity on the relationship between emotionally disturbing work and employee health. *Journal of Occupational Health Psychology* **2022**, *27*, 488-502, doi:10.1037/ocp0000331.
38. Bai, S.; Buxton, O.M.; Master, L.; Hale, L. Daily associations between family interaction quality, stress, and objective sleep in adolescents. *Sleep Health* **2022**, *8*, 69-72, doi:10.1016/j.sleh.2021.11.006.

39. Bai, Y.; Copeland, W.E.; Burns, R.; Nardone, H.; Devadanam, V.; Rettew, J.; Hudziak, J. Ecological Momentary Assessment of Physical Activity and Wellness Behaviors in College Students Throughout a School Year: Longitudinal Naturalistic Study. *JMIR Public Health and Surveillance* **2022**, *8*, e25375, doi:10.2196/25375.
40. Balouch, S.; Dijk, D.A.D.; Rusted, J.; Skene, S.S.; Tabet, N.; Dijk, D.J. Night-to-night variation in sleep associates with day-to-day variation in vigilance, cognition, memory, and behavioral problems in Alzheimer's disease. *Alzheimer's & Dementia: Diagnosis, Assessment & Disease Monitoring* **2022**, *14*, e12303, doi:10.1002/dad2.12303.
41. Blok, E.; Koopman-Verhoeff, M.E.; Dickstein, D.P.; Saletin, J.; Luik, A.I.; Rijlaarsdam, J.; Hillegers, M.; Kocevska, D.; White, T.; Tiemeier, H. Sleep and mental health in childhood: a multi-method study in the general pediatric population. *Child and Adolescent Psychiatry and Mental Health* **2022**, *16*, 11, doi:10.1186/s13034-022-00447-0.
42. Bonnar, D.; Lee, S.; Roane, B.M.; Blum, D.J.; Kahn, M.; Jang, E.; Dunican, I.C.; Gradisar, M.; Suh, S. Evaluation of a Brief Sleep Intervention Designed to Improve the Sleep, Mood, and Cognitive Performance of Esports Athletes. *Journal of Environmental Research and Public Health* **2022**, *19*, 4146, doi:10.3390/ijerph19074146.
43. Burke, T.A.; Hamilton, J.L.; Seigel, D.; Kautz, M.; Liu, R.T.; Alloy, L.B.; Barker, D.H. Sleep irregularity and nonsuicidal self-injurious urges and behaviors. *Sleep* **2022**, *45*, doi:10.1093/sleep/zsac084.
44. Dominiak, M.; Kaczmarek-Majer, K.; Antosik-Wojcinska, A.Z.; Opara, K.R.; Olwert, A.; Radziszewska, W.; Hryniewicz, O.; Swiecicki, L.; Wojnar, M.; Mierzejewski, P. Behavioral and Self-reported Data Collected From Smartphones for the Assessment of Depressive and Manic Symptoms in Patients With Bipolar Disorder: Prospective Observational Study. *Journal of Medical Internet Research* **2022**, *24*, e28647, doi:10.2196/28647.
45. Goyer, J.P.; Akinola, M.; Grunberg, R.; Crum, A.J. Thriving under pressure: The effects of stress-related wise interventions on affect, sleep, and exam performance for college students from disadvantaged backgrounds. *Emotion* **2022**, *22*, 1755-1772, doi:10.1037/emo0001026.
46. Hamilton, N.; Freche, R.; Zhang, Y.; Zeller, G.; Carroll, I. Test Anxiety and Poor Sleep: A Vicious Cycle. *International Journal of Behavioral Medicine* **2021**, *28*, 250-258, doi:10.1007/s12529-021-09973-1.
47. Horwitz, A.; Czyz, E.; Al-Dajani, N.; Dempsey, W.; Zhao, Z.; Nahum-Shani, I.; Sen, S. Utilizing daily mood diaries and wearable sensor data to predict depression and suicidal ideation among medical interns. *Journal of Affective Disorders* **2022**, *313*, 1-7, doi:10.1016/j.jad.2022.06.064.
48. Kabir, K.S.; Flis, A.; Mickens, M.; Trapp, S.K.; Wiese, J. "We're Not Meant to Deal with Crisis for a Year": Supporting Frontline Healthcare Providers' Wellness During a Pandemic. 2022; pp. 147-163.
49. Kivela, L.; Riese, H.; Fakkel, T.G.; Verkuil, B.; Penninx, B.; Lamers, F.; van der Does, W.; Antypa, N. Chronotype, daily affect and social contact: An ecological momentary assessment study. *Psychiatry Research* **2022**, *309*, 114386, doi:10.1016/j.psychres.2021.114386.
50. Krohn, H.; Guintivano, J.; Frische, R.; Steed, J.; Rackers, H.; Meltzer-Brody, S. App-Based Ecological Momentary Assessment to Enhance Clinical Care for Postpartum Depression: Pilot Acceptability Study. *JMIR Formative Research* **2022**, *6*, e28081, doi:10.2196/28081.

51. Le, F.; Yap, Y.; Tung, N.Y.C.; Bei, B.; Wiley, J.F. The Associations Between Daily Activities and Affect: a Compositional Isotemporal Substitution Analysis. *International Journal of Behavioral Medicine* **2022**, *29*, 456-468, doi:10.1007/s12529-021-10031-z.
52. Lewis, K.J.S.; Tilling, K.; Gordon-Smith, K.; Saunders, K.E.A.; Di Florio, A.; Jones, L.; Jones, I.; O'Donovan, M.C.; Heron, J. The dynamic interplay between sleep and mood: an intensive longitudinal study of individuals with bipolar disorder. *Psychological Medicine* **2023**, *53*, 3345-3354, doi:10.1017/S0033291721005377.
53. Manasse, S.M.; Haedt-Matt, A.A.; Smith, K.E.; Egbert, A.H.; O'Sullivan, K.; Koren, D.; Engel, S.; Goldschmidt, A.B. The moderating role of sleep duration on momentary relations between negative affect and loss-of-control eating in children and adolescents. *European Eating Disorders Review* **2022**, *30*, 815-822, doi:10.1002/erv.2908.
54. Massar, S.A.A.; Ng, A.S.C.; Soon, C.S.; Ong, J.L.; Chua, X.Y.; Chee, N.; Lee, T.S.; Chee, M.W.L. Reopening after lockdown: the influence of working-from-home and digital device use on sleep, physical activity, and wellbeing following COVID-19 lockdown and reopening. *Sleep* **2022**, *45*, doi:10.1093/sleep/zsab250.
55. Moilanen, S.; Autio, L.; Tolvanen, A.; Sevón, E.; Rönkä, A. From Intense to Leisurely Study Days: A Diary Study of Daily Wellbeing among Students in Higher Education. *Open Education Studies* **2020**, *2*, 295-311, doi:10.1515/edu-2020-0133.
56. Newman, D.B.; Gordon, A.M.; Mendes, W.B. Comparing daily physiological and psychological benefits of gratitude and optimism using a digital platform. *Emotion* **2021**, *21*, 1357-1365, doi:10.1037/emo0001025.
57. Niemeijer, K.; Mestdagh, M.; Kuppens, P. Tracking Subjective Sleep Quality and Mood With Mobile Sensing: Multiverse Study. *Journal of Medical Internet Research* **2022**, *24*, e25643, doi:10.2196/25643.
58. O'Rourke, N.; Sixsmith, A. Ecological momentary assessment of mood and movement with bipolar disorder over time: Participant recruitment and efficacy of study methods. *International Journal of Methods in Psychiatric Research* **2021**, *30*, e1895, doi:10.1002/mpr.1895.
59. Park, L.S.; Martinez, L.; Xu, S. Job experience as a buffer against incivility: a daily diary study. *Journal of Managerial Psychology* **2022**, *37*, 243-263, doi:10.1108/jmp-03-2021-0194.
60. Parsons, C.E.; Young, K.S. Beneficial effects of sleep extension on daily emotion in short-sleeping young adults: An experience sampling study. *Sleep Health* **2022**, *8*, 505-513, doi:10.1016/j.sleh.2022.05.008.
61. Riedy, D.R.; MacPherson, A.R.; Sabet, S.; Dautovich, N.D. Examining negative affect, sleep duration, and using food to cope as predictors of weight in midlife women. *Journal of Behavioral Medicine* **2022**, *45*, 894-903, doi:10.1007/s10865-022-00338-x.
62. Şansal, K.E. Relationship of daily time spent outdoors with sleep quality and emotional well-being among community-dwelling older adults during COVID-19 restrictions. *Turkish Journal of Geriatrics* **2021**, *24*, 424-432, doi:10.31086/tjgeri.2021.240.
63. Sletten, T.L.; Sullivan, J.P.; Arendt, J.; Palinkas, L.A.; Barger, L.K.; Fletcher, L.; Arnold, M.; Wallace, J.; Strauss, C.; Baker, R.J.S.; et al. The role of circadian phase in sleep and performance during Antarctic winter expeditions. *Journal of Pineal Research* **2022**, *73*, e12817, doi:10.1111/jpi.12817.

64. Wieman, S.T.; Fields, J.S.; Arditte Hall, K.A.; MacDonald, H.Z.; Liverant, G.I. Effects of the COVID-19 pandemic on anhedonia, reward exposure and responsiveness, and sleep in college students. *Journal of American College Health* **2022**, 1-5, doi:10.1080/07448481.2022.2047705.
65. Willroth, E.C.; Gatchpazian, A.; Thai, S.; Lassetter, B.; Feinberg, M.; Ford, B.Q. The Insulating Function of Sleep for Well-being: Daily Sleep Quality Attenuates the Link Between Current Affect and Global Life Satisfaction. *Affective Science* **2022**, *3*, 318-329, doi:10.1007/s42761-021-00092-4.
66. Xia, C.H.; Barnett, I.; Tapera, T.M.; Adebimpe, A.; Baker, J.T.; Bassett, D.S.; Brotman, M.A.; Calkins, M.E.; Cui, Z.; Leibenluft, E.; et al. Mobile footprinting: linking individual distinctiveness in mobility patterns to mood, sleep, and brain functional connectivity. *Neuropsychopharmacology* **2022**, *47*, 1662-1671, doi:10.1038/s41386-022-01351-z.
67. Yap, Y.; Bei, B.; Wiley, J.F. Daily coping moderates the relations between stress and actigraphic sleep: a daily intensive longitudinal study with ecological momentary assessments. *Sleep Medicine* **2021**, *88*, 231-240, doi:10.1016/j.sleep.2021.10.012.
68. Zhang, B.; Zhao, L.; Liu, X.; Bu, Y.; Ren, Y. The Influence of Employee Emotion Fluctuation on Service Performance: An Experience Sampling Data Analysis. *Frontiers in Psychology* **2022**, *13*, 648142, doi:10.3389/fpsyg.2022.648142.
69. Zubek, J.; Ziembowicz, K.; Pokropski, M.; Gwiazdzinski, P.; Denkiewicz, M.; Boros, A. Rhythms of the day: How electronic media and daily routines influence mood during COVID-19 pandemic. *Applied Psychology: Health and Well-Being* **2022**, *14*, 519-536, doi:10.1111/aphw.12317.
70. Zuidersma, M.; Lugtenburg, A.; van Zelst, W.; Reesink, F.E.; De Deyn, P.P.; Strijkert, F.; Zuidema, S.U.; Oude Voshaar, R.C. Temporal dynamics of depression, cognitive performance and sleep in older persons with depressive symptoms and cognitive impairments: a series of eight single-subject studies. *International Psychogeriatrics* **2022**, *34*, 47-59, doi:10.1017/S1041610221000065.
71. Amon, M.J.; Mattingly, S.; Necaise, A.; Mark, G.; Chawla, N.; Dey, A.; D'Mello, S. Flexibility Versus Routineness in Multimodal Health Indicators: A Sensor-based Longitudinal in Situ Study of Information Workers. *ACM Transactions on Computing for Healthcare* **2022**, *3(3)*, doi:10.1145/3514259.
72. Barbeau, K.; Turpin, C.; Lafreniere, A.; Campbell, E.; De Koninck, J. Dreamers' evaluation of the emotional valence of their day-to-day dreams is indicative of some mood regulation function. *Frontiers in Behavioral Neuroscience* **2022**, *16*, doi:10.3389/fnbeh.2022.947396.
73. Lim, J.A.; Yun, J.Y.; Choi, S.H.; Park, S.; Suk, H.W.; Jang, J.H. Greater variability in daily sleep efficiency predicts depression and anxiety in young adults: Estimation of depression severity using the two-week sleep quality records of wearable devices. *Frontiers in Psychiatry* **2022**, *13*, doi:10.3389/fpsyt.2022.1041747.
74. Walsh, R.F.L.; Smith, L.T.; Titone, M.K.; Ng, T.H.; Goel, N.; Alloy, L.B. The relationship between physical activity states and depressive symptoms: Using ambulatory assessment to characterize day-to-day associations among individuals with and without bipolar spectrum disorder. *Depression and Anxiety* **2022**, *39(12)*, 835-844, doi:10.1002/da.23290.
75. Asiamah-Asare, B.K.Y.; Robinson, S.; Powell, D.; Kwasnicka, D. Impact of fly-in fly-out work on health behaviours and affective states: A daily diary study. *Stress and Health* **2023**, doi:10.1002/smi.3361.

76. Ballesio, A.; Zagaria, A.; Salaris, A.; Terrasi, M.; Lombardo, C.; Ottaviani, C. Sleep and daily positive emotions-Is heart rate variability a mediator? *Journal of Psychophysiology* **2023**, *37*, 134-142, doi:10.1027/0269-8803/a000315.
77. Bundo, M.; Preisig, M.; Merikangas, K.; Glaus, J.; Vaucher, J.; Waeber, G.; Marques-Vidal, P.; Strippoli, M.F.; Muller, T.; Franco, O.; et al. How ambient temperature affects mood: an ecological momentary assessment study in Switzerland. *Environmental Health: A Global Access Science Source* **2023**, *22*, 52, doi:10.1186/s12940-023-01003-9.
78. Frerart, L.; Bilsen, L.; Dejonckheere, E.; Kuppens, P. Overnight emotional inertia in relation to depressive symptomatology and subjective sleep quality. *SLEEP Advances* **2023**, *4*, doi:10.1093/sleepadvances/zpac048.
79. Junca-Silva, A.; Mendes, S. The intersectional effects of motivational and affective factors on managers' performance. *Applied Psychology: Health and Well-Being* **2023**, *15*, 1619-1636, doi:10.1111/aphw.12458.
80. Lee, S.; Mu, C.X.; Joshi, R.; Khan, A. Daily and momentary variability in sleep, stress, and well-being data in two samples of health care workers. *Field Methods* **2023**, *35*, 349-363, doi:10.1177/1525822X221132425.
81. Massar, S.A.A.; Ong, J.L.; Lau, T.; Ng, B.K.L.; Chan, L.F.; Koek, D.; Cheong, K.; Chee, M.W.L. Working-from-home persistently influences sleep and physical activity 2 years after the Covid-19 pandemic onset: a longitudinal sleep tracker and electronic diary-based study. *Frontiers in Psychology* **2023**, *14*, 1145893, doi:10.3389/fpsyg.2023.1145893.
82. Mathew, G.M.; Reichenberger, D.A.; Master, L.; Buxton, O.M.; Chang, A.M.; Hale, L. Actigraphic sleep variability is associated with lower positive mood in adolescents. *Journal of Adolescent Health* **2023**, *73*, 478-485, doi:10.1016/j.jadohealth.2023.04.019.
83. Menghini, L.; Yuksel, D.; Prouty, D.; Baker, F.C.; King, C.; de Zambotti, M. Wearable and mobile technology to characterize daily patterns of sleep, stress, presleep worry, and mood in adolescent insomnia. *Sleep Health* **2023**, *9(1)*, 108-116, doi:10.1016/j.sleh.2022.11.006.
84. Nakagawa, T.; Yasumoto, S.; Kabayama, M.; Matsuda, K.; Gondo, Y.; Kamide, K.; Ikebe, K. Association between prior-night sleep and next-day fatigue in older adults: a daily diary study. *BMC Geriatrics* **2023**, *23(1)*, 817, doi:10.1186/s12877-023-04539-0.
85. Newman, D.B.; Gordon, A.M.; Prather, A.A.; Berry Mendes, W. Examining Daily Associations Among Sleep, Stress, and Blood Pressure Across Adulthood. *Annals of Behavioral Medicine* **2023**, *57(6)*, 453-462, doi:10.1093/abm/kaac074.
86. Smith, L.T.; Walsh, R.F.; Goel, N.; Alloy, L.B. Social jetlag and trajectories of mood symptoms and reward responsiveness in individuals at low-risk, high-risk, and with bipolar spectrum disorders: An ecological momentary assessment study. *Psychiatry Research* **2023**, *329*, 1-10, doi:10.1016/j.psychres.2023.115499.
87. Speyer, L.G.; Murray, A.L.; Kievit, R. Investigating Moderation Effects at the Within-Person Level Using Intensive Longitudinal Data: A Two-Level Dynamic Structural Equation Modelling Approach in Mplus. *Multivariate Behavioral Research* **2023**, doi:10.1080/00273171.2023.2288575.
88. Stapp, E.K.; Zipunnikov, V.; Leroux, A.; Cui, L.; Husky, M.M.; Dey, D.; Merikangas, K.R. Specificity of affective dynamics of bipolar and major depressive disorder. *Brain and Behavior* **2023**, *13(9)*, doi:10.1002/brb3.3134.

89. Tang, N.K.; Banks, P.D.; Sanborn, A.N. Judgement of sleep quality of the previous night changes as the day unfolds: A prospective experience sampling study. *Journal of Sleep Research* **2023**, *32*, 1-10, doi:10.1111/jsr.13764.
90. Urwin, S.; Lau, Y.S.; Grande, G.; Sutton, M. Informal caregiving, time use and experienced wellbeing. *Health Economics* **2023**, *32*, 356-374, doi:10.1002/hec.4624.
91. Walsh, R.F.; Smith, L.T.; Klugman, J.; Titone, M.K.; Ng, T.H.; Goel, N.; Alloy, L.B. An examination of bidirectional associations between physical activity and mood symptoms among individuals diagnosed and at risk for bipolar spectrum disorders. *Behaviour Research and Therapy* **2023**, *161*, 1-10, doi:10.1016/j.brat.2023.104255.
92. Whiston, A.; Igou, E.R.; Fortune, D.G.; Analog Devices, T.; Semkovska, M. Examining Stress and Residual Symptoms in Remitted and Partially Remitted Depression Using a Wearable Electrodermal Activity Device: A Pilot Study. *IEEE Journal of Translational Engineering in Health and Medicine* **2023**, *11*, 96-106, doi:10.1109/JTEHM.2022.3228483.
93. Whiston, A.; Igou, E.R.; Fortune, D.G.; Semkovska, M. Longitudinal interactions between residual symptoms and physiological stress in the remitted symptom network structure of depression. *Acta Psychologica* **2023**, *241*, 104078, doi:10.1016/j.actpsy.2023.104078.
94. Xu, J.; Wang, H.; Liu, S.; Hale, M.E.; Weng, X.; Ahemaitijiang, N.; Hu, Y.; Suveg, C.; Han, Z.R. Relations among family, peer, and academic stress and adjustment in Chinese adolescents: A daily diary analysis. *Developmental Psychology* **2023**, *59*, 1346-1358, doi:10.1037/dev0001538.
95. Zhang, H.; Zhou, Z.E.; Zhang, L.; Liu, Y.; Shi, Y. How customer mistreatment hinders employee sleep quality and next-morning vigor: The effects of affective rumination and mindfulness. *Applied Psychology* **2023**, doi:10.1111/apps.12507.
96. Zhu, Y.; Martin, A.; Kane, H.; Park, J. Is daily emotion suppression associated with poor sleep? The moderating role of culture. *Emotion* **2023**, *23*, 1829-1843, doi:10.1037/emo0001206.
97. Zhu, Y.; Zhao, C.; Zhuang, J.Y. Examination of daily abusive supervision effects on next-day employee wellbeing: a spillover perspective. *Australian Journal of Psychology* **2023**, *75*, doi:10.1080/00049530.2023.2264938.
98. Chen, S.T.; Yang, C.H.; Hyun, J.; Ku, P.W. The association between morning pleasant anticipation and daily positive incidental affect on adults' daily steps: An ecological momentary assessment study. *Psychology of Sport & Exercise* **2024**, *70*, 102561, doi:10.1016/j.psychsport.2023.102561.
99. Dickman, K.D.; Thomas, M.C.; Chin, B.N.; Kamarck, T.W. Bidirectional Associations Between Loneliness, Emotional Support, and Sleep in Daily Life. *Psychosomatic Medicine* **2024**, *86(4)*, 252-260, doi:10.1097/PSY.0000000000001291.
100. Hoyniak, C.P.; Vogel, A.C.; Puricelli, A.; Luby, J.L.; Whalen, D.J. Day-to-day bidirectional associations between sleep and emotion states in early childhood: Importance of end-of-day mood for sleep quality. *Sleep Health* **2024**, *28*, doi:10.1016/j.sleh.2023.12.007.
101. Kinoshita, S.; Hanashiro, S.; Tsutsumi, S.; Shiga, K.; Kitazawa, M.; Wada, Y.; Inaishi, J.; Kashiwagi, K.; Fukami, T.; Mashimo, Y.; et al. Assessment of Stress and Well-Being of Japanese Employees Using Wearable Devices for Sleep Monitoring Combined With Ecological Momentary Assessment: Pilot Observational Study. *JMIR Formative Research* **2024**, *8*, e49396, doi:10.2196/49396.
102. Labbaf, S.; Abbasian, M.; Nguyen, B.; Lucero, M.; Ahmed, M.S.; Yunusova, A.; Rivera, A.; Jain, R.; Borelli, J.L.; Dutt, N.; et al. Physiological and emotional assessment of college students using

wearable and mobile devices during the 2020 COVID-19 lockdown: An intensive, longitudinal dataset. *Data in Brief* **2024**, *54*, doi:10.1016/j.dib.2024.110228.

103. Lenneis, A.; Das-Friebel, A.; Tang, N.K.; Sanborn, A.N.; Lemola, S.; Singmann, H.; Wolke, D.; von Muhlenen, A.; Realo, A. The influence of sleep on subjective well-being: An experience sampling study. *Emotion* **2024**, *24*, 451-464, doi:10.1037/emo0001268.
104. Li, Y.N.; Cheng, B.H.; Yu, B.; Zhu, J.N.Y. Let's get physical! A time-lagged examination of the motivation for daily physical activity and implications for next-day performance and health. *Personnel Psychology* **2024**, *77*, 917-955, doi:10.1111/peps.12585.
105. Palatine, E.; Phillips, M.L.; Soehner, A.M. The effect of slow wave sleep deprivation on mood in adolescents with depressive symptoms: A pilot study. *Journal of Affective Disorders* **2024**, *354*, 347-355, doi:10.1016/j.jad.2024.03.058.
106. Siepe, B.S.; Sander, C.; Schultze, M.; Kliem, A.; Ludwig, S.; Hegerl, U.; Reich, H. Time-Varying Network Models for the Temporal Dynamics of Depressive Symptomatology in Patients With Depressive Disorders: Secondary Analysis of Longitudinal Observational Data. *JMIR Mental Health* **2024**, *11(1)*, doi:10.2196/50136.
107. Song, Y.M.; Jeong, J.; de los Reyes, A.A.; Lim, D.; Cho, C.H.; Yeom, J.W.; Lee, T.; Lee, J.B.; Lee, H.J.; Kim, J.K. Causal dynamics of sleep, circadian rhythm, and mood symptoms in patients with major depression and bipolar disorder: insights from longitudinal wearable device data. *eBioMedicine* **2024**, *103*, doi:10.1016/j.ebiom.2024.105094.
108. Toenders, Y.J.; van der Cruijsen, R.; Runze, J.; van de Groep, S.; Wierenga, L.; Crone, E.A. Mood variability during adolescent development and its relation to sleep and brain development. *Scientific Reports* **2024**, *14*, 8537, doi:10.1038/s41598-024-59227-9.
109. Wen, J.H.; Klaiber, P.; Leger, K.A.; Hill, P.L.; Pfund, G.N.; Slavish, D.C.; Delongis, A.; Sin, N.L. Nightly Sleep Predicts Next-Morning Expectations for Stress and Positive Experiences. *Psychosomatic Medicine* **2024**, *86(4)*, 261-271, doi:10.1097/PSY.0000000000001303.
110. Wijnhoven, H.A.H.; Kok, A.A.L.; Schaap, L.A.; Hoekstra, T.; van Stralen, M.M.; Twisk, J.W.R.; Visser, M. The associations between sleep quality, mood, pain and appetite in community dwelling older adults: a daily experience study. *Journal of Nutrition, Health and Aging* **2024**, *28(2)*, doi:10.1016/j.jnha.2023.100028.
111. Xu, J.; Liu, M.; Li, L.; Xia, Z. Effects of environmental lighting on students’ sleep, alertness and mood: A field study in a Chinese boarding school. *Lighting Research and Technology* **2024**, *56*, 185-206, doi:10.1177/14771535231165263.
112. Yang, Z.; Wang, Y.; Yamashita, K.S.; Khatibi, E.; Azimi, I.; Dutt, N.; Borelli, J.L.; Rahmani, A.M. Integrating wearable sensor data and self-reported diaries for personalized affect forecasting. *Smart Health* **2024**, *32*, doi:10.1016/j.smhl.2024.100464.
113. Moretti, M.M. Affect regulation checklist. *Unpublished measure and data. British Columbia, Canada: Simon Fraser University* **2003**.
114. George, M.J.; Rivenbark, J.G.; Russell, M.A.; Ng'eno, L.; Hoyle, R.H.; Odgers, C.L. Evaluating the Use of Commercially Available Wearable Wristbands to Capture Adolescents' Daily Sleep Duration. *Journal of Research on Adolescence* **2019**, *29*, 613-626, doi:10.1111/jora.12467.
115. Altman, E.G.; Hedeker, D.; Peterson, J.L.; Davis, J.M. The Altman Self-Rating Mania Scale. *Biological Psychiatry* **1997**, *42*, 948-955, doi:10.1016/S0006-3223(96)00548-3.

116. Tseng, Y.C.; Lin, E.C.; Wu, C.H.; Huang, H.L.; Chen, P.S. Associations among smartphone app-based measurements of mood, sleep and activity in bipolar disorder. *Psychiatry Research* **2022**, *310*, 114425, doi:10.1016/j.psychres.2022.114425.
117. Titone, M.K.; Goel, N.; Ng, T.H.; MacMullen, L.E.; Alloy, L.B. Impulsivity and sleep and circadian rhythm disturbance predict next-day mood symptoms in a sample at high risk for or with recent-onset bipolar spectrum disorder: An ecological momentary assessment study. *Journal of Affective Disorders* **2022**, *298*, 17-25, doi:10.1016/j.jad.2021.08.155.
118. Beck, A.T.; Steer, R.A.; Brown, G. Beck depression inventory–II. *Psychological Assessment* **1996**, doi:10.1037/t00742-000.
119. Romans, S.E.; Kreindler, D.; Asllani, E.; Einstein, G.; Laredo, S.; Levitt, A.; Morgan, K.; Petrovic, M.; Toner, B.; Stewart, D.E. Mood and the menstrual cycle. *Psychotherapy and Psychosomatics* **2013**, *82*, 53-60, doi:10.1159/000339370.
120. Li, D.X.; Romans, S.; De Souza, M.J.; Murray, B.; Einstein, G. Actigraphic and self-reported sleep quality in women: associations with ovarian hormones and mood. *Sleep Medicine* **2015**, *16*, 1217-1224, doi:10.1016/j.sleep.2015.06.009.
121. Lee, S.; Mu, C.X.; Joshi, R.; Khan, A. Daily and Momentary Variability in Sleep, Stress, and Well-being Data in Two Samples of Health Care Workers. *Field Methods* **2022**, 35(34), doi:10.1177/1525822X221132425.
122. Poon, C.Y.; Cheng, Y.C.; Wong, V.W.H.; Tam, H.K.; Chung, K.F.; Yeung, W.F.; Ho, F.Y.Y. Directional associations among real-time activity, sleep, mood, and daytime symptoms in major depressive disorder using actigraphy and ecological momentary assessment. *Behaviour Research and Therapy* **2024**, *173*, 1-8, doi:10.1016/j.brat.2023.104464.
123. Lovibond, P.F.; Lovibond, S.H. The structure of negative emotional states: comparison of the Depression Anxiety Stress Scales (DASS) with the Beck Depression and Anxiety Inventories. *Behaviour Research and Therapy* **1995**, *33*, 335-343, doi:10.1016/0005-7967(94)00075-u.
124. Almeida, D.M.; Wethington, E.; Kessler, R.C. The daily inventory of stressful events: an interview-based approach for measuring daily stressors. *Assessment* **2002**, *9*, 41-55, doi:10.1177/1073191102091006.
125. Ong, A.D.; Exner-Cortens, D.; Riffin, C.; Steptoe, A.; Zautra, A.; Almeida, D.M. Linking Stable and Dynamic Features of Positive Affect to Sleep. *Annals of Behavioral Medicine* **2013**, *46*, 52-61, doi:10.1007/s12160-013-9484-8.
126. Fredrickson, B.L.; Tugade, M.M.; Waugh, C.E.; Larkin, G.R. What good are positive emotions in crises? A prospective study of resilience and emotions following the terrorist attacks on the United States on September 11th, 2001. *Journal of Personality and Social Psychology* **2003**, *84*, 365-376, doi:10.1037//0022-3514.84.2.365.
127. Harris, P.E.; Gordon, A.M.; Dover, T.L.; Small, P.A.; Collins, N.L.; Major, B. Sleep, Emotions, and Sense of Belonging: A Daily Experience Study. *Affective Science* **2022**, *3*, 295-306, doi:10.1007/s42761-021-00088-0.
128. Newman, D.B.; Epel, E.S.; Coccia, M.; Puterman, E.; Prather, A.A. Asymmetrical Effects of Sleep and Emotions in Daily Life. *Affective Science* **2022**, *3*, 307-317, doi:10.1007/s42761-022-00112-x.
129. Casillas, A.; Clark, L.A. The mini mood and anxiety symptom questionnaire (Mini-MASQ). **2000**.
130. Watson, D.; Clark, L.A.; Weber, K.; Assenheimer, J.S.; Strauss, M.E.; McCormick, R.A. Testing a tripartite model: II. Exploring the symptom structure of anxiety and depression in student, adult,

and patient samples. *Journal of Abnormal Psychology* **1995**, *104*, 15-25, doi:10.1037//0021-843x.104.1.15.
131. Kalmbach, D.A.; Arnedt, J.T.; Swanson, L.M.; Rapier, J.L.; Ciesla, J.A. Reciprocal dynamics between self-rated sleep and symptoms of depression and anxiety in young adult women: a 14-day diary study. *Sleep Medicine* **2017**, *33*, 6-12, doi:10.1016/j.sleep.2016.03.014.
132. Bean, C.A.L.; Ciesla, J.A. Naturalistic Partial Sleep Deprivation Leads to Greater Next-Day Anxiety: The Moderating Role of Baseline Anxiety and Depression. *Behavior Therapy* **2021**, *52*, 861-873, doi:10.1016/j.beth.2020.10.008.
133. Steyer, R.; Eid, M.; Schwenkmezger, P. Modeling true intraindividual change: True change as a latent variable. *Methods of Psychological Research Online* **1997**, *2*, 21-33, doi:10.23668/psycharchives.12718.
134. Reis, D.; Arndt, C.; Lischetzke, T.; Hoppe, A. State work engagement and state affect: Similar yet distinct concepts. *Journal of Vocational Behavior* **2016**, *93*, 1-10, doi:10.1016/j.jvb.2015.12.004.
135. Warr, P.; Bindl, U.K.; Parker, S.K.; Inceoglu, I. Four-quadrant investigation of job-related affects and behaviours. *European Journal of Work and Organizational Psychology* **2013**, *23*, 342-363, doi:10.1080/1359432x.2012.744449.
136. Williamson, A.J.; Battisti, M.; Leatherbee, M.; Gish, J.J. Rest, Zest, and My Innovative Best: Sleep and Mood as Drivers of Entrepreneurs' Innovative Behavior. *Entrepreneurship Theory and Practice* **2018**, *43*, 582-610, doi:10.1177/1042258718798630.
137. Kessler, R.C.; Andrews, G.; Colpe, L.J.; Hiripi, E.; Mroczek, D.K.; Normand, S.L.; Walters, E.E.; Zaslavsky, A.M. Short screening scales to monitor population prevalences and trends in non-specific psychological distress. *Psychological Medicine* **2002**, *32*, 959-976, doi:10.1017/s0033291702006074.
138. Zarit, S.H.; Kim, K.; Femia, E.E.; Almeida, D.M.; Klein, L.C. The effects of adult day services on family caregivers' daily stress, affect, and health: outcomes from the Daily Stress and Health (DaSH) study. *Gerontologist* **2014**, *54*, 570-579, doi:10.1093/geront/gnt045.
139. Liu, Y.; Leggett, A.N.; Kim, K.; Polenick, C.A.; McCurry, S.M.; Zarit, S.H. Daily sleep, well-being, and adult day services use among dementia care dyads. *Aging & Mental Health* **2022**, *26*, 2472-2480, doi:10.1080/13607863.2021.1998354.
140. Barber, K.E.; Rackoff, G.N.; Newman, M.G. Day-to-day directional relationships between sleep duration and negative affect. *Journal of Psychosomatic Research* **2023**, *172*, doi:10.1016/j.jpsychores.2023.111437.
141. Watson, D.; Clark, L.A.; Tellegen, A. Development and validation of brief measures of positive and negative affect: the PANAS scales. *Journal of Personality and Social Psychology* **1988**, *54*, 1063-1070, doi:10.1037//0022-3514.54.6.1063.
142. Laurent, J.; Catanzaro, S.J.; Joiner, T.E.; Rudolph, K.D.; Potter, K.I.; Lambert, S.; Osborne, L.; Gathright, T. A measure of positive and negative affect for children: Scale development and preliminary validation. *Psychological Assessment* **1999**, *11*, 326-338, doi:10.1037/1040-3590.11.3.326.
143. Watson, D.; Clark, L.A. The PANAS-X: Manual for the positive and negative affect schedule-expanded form. **1994**, doi:10.17077/48vt-m4t2.
144. Thompson, E.R. Development and Validation of an Internationally Reliable Short-Form of the Positive and Negative Affect Schedule (PANAS). *Journal of Cross-Cultural Psychology* **2016**, *38*, 227-242, doi:10.1177/0022022106297301.

145. Blaxton, J.M.; Bergeman, C.S.; Whitehead, B.R.; Braun, M.E.; Payne, J.D. Relationships Among Nightly Sleep Quality, Daily Stress, and Daily Affect. *Journals of Gerontology Series B: Psychological Sciences and Social Sciences* **2017**, *72*, 363-372, doi:10.1093/geronb/gbv060.
146. Bouwmans, M.E.J.; Bos, E.H.; Hoenders, H.J.R.; Oldehinkel, A.J.; de Jonge, P. Sleep quality predicts positive and negative affect but not vice versa. An electronic diary study in depressed and healthy individuals. *Journal of Affective Disorders* **2017**, *207*, 260-267, doi:10.1016/j.jad.2016.09.046.
147. Cousins, J.C.; Whalen, D.J.; Dahl, R.E.; Forbes, E.E.; Olino, T.M.; Ryan, N.D.; Silk, J.S. The bidirectional association between daytime affect and nighttime sleep in youth with anxiety and depression. *Journal of Pediatric Psychology* **2011**, *36*, 969-979, doi:10.1093/jpepsy/jsr036.
148. da Estrela, C.; Barker, E.T.; Lantagne, S.; Gouin, J.P. Chronic parenting stress and mood reactivity: The role of sleep quality. *Stress Health* **2018**, *34*, 296-305, doi:10.1002/smi.2790.
149. Das-Friebel, A.; Lenneis, A.; Realo, A.; Sanborn, A.; Tang, N.K.Y.; Wolke, D.; von Muhlenen, A.; Lemola, S. Bedtime social media use, sleep, and affective wellbeing in young adults: an experience sampling study. *Journal of Child Psychology and Psychiatry* **2020**, *61*, 1138-1149, doi:10.1111/jcpp.13326.
150. Díaz-Morales, J.F.; Parra-Robledo, Z. Day-of-week mood patterns in adolescents considering chronotype, sleep length and sex. *Personality and Individual Differences* **2021**, *179*, 110951, doi:10.1016/j.paid.2021.110951.
151. Doane, L.D.; Thurston, E.C. Associations among sleep, daily experiences, and loneliness in adolescence: evidence of moderating and bidirectional pathways. *Journal of Adolescence* **2014**, *37*, 145-154, doi:10.1016/j.adolescence.2013.11.009.
152. Fortier, M.S.; Guerin, E.; Williams, T.; Strachan, S. Should I exercise or sleep to feel better? A daily analysis with physically active working mothers. *Mental Health and Physical Activity* **2015**, *8*, 56-61, doi:10.1016/j.mhpa.2015.03.001.
153. Galambos, N.L.; Dalton, A.L.; Maggs, J.L. Losing Sleep Over It: Daily Variation in Sleep Quantity and Quality in Canadian Students' First Semester of University. *Journal of Research on Adolescence* **2009**, *19*, 741-761, doi:10.1111/j.1532-7795.2009.00618.x.
154. Garcia, C.; Zhang, L.; Holt, K.; Hardeman, R.; Peterson, B. Latina adolescent sleep and mood: an ecological momentary assessment pilot study. *Journal of Child and Adolescent Psychiatric Nursing* **2014**, *27*, 132-141, doi:10.1111/jcap.12082.
155. Gershon, A.; Thompson, W.K.; Eidelman, P.; McGlinchey, E.L.; Kaplan, K.A.; Harvey, A.G. Restless pillow, ruffled mind: sleep and affect coupling in interepisode bipolar disorder. *Journal of Abnormal Psychology* **2012**, *121*, 863-873, doi:10.1037/a0028233.
156. Jones, D.R.; Smyth, J.M.; Graham-Engeland, J.E. Associations between positively valenced affect and health behaviors vary by arousal. *Applied Psychology: Health and Well-Being* **2022**, *14*, 215-235, doi:10.1111/aphw.12299.
157. Kalmbach, D.A.; Pillai, V.; Roth, T.; Drake, C.L. The interplay between daily affect and sleep: a 2-week study of young women. *Journal of Sleep Research* **2014**, *23*, 636-645, doi:10.1111/jsr.12190.
158. Leger, K.A.; Charles, S.T.; Fingerman, K.L. Affect variability and sleep: Emotional ups and downs are related to a poorer night's rest. *Journal of Psychosomatic Research* **2019**, *124*, 109758, doi:10.1016/j.jpsychores.2019.109758.

159. McCrae, C.S.; Dzierzewski, J.M.; McNamara, J.P.; Vatthauer, K.E.; Roth, A.J.; Rowe, M.A. Changes in Sleep Predict Changes in Affect in Older Caregivers of Individuals with Alzheimer's Dementia: A Multilevel Model Approach. *Journals of Gerontology Series B: Psychological Sciences and Social Sciences* **2016**, *71*, 458-462, doi:10.1093/geronb/gbu162.
160. McCrae, C.S.; McNamara, J.P.; Rowe, M.A.; Dzierzewski, J.M.; Dirk, J.; Marsiske, M.; Craggs, J.G. Sleep and affect in older adults: using multilevel modeling to examine daily associations. *Journal of Sleep Research* **2008**, *17*, 42-53, doi:10.1111/j.1365-2869.2008.00621.x.
161. McGrath, E.; Cooper-Thomas, H.D.; Garrosa, E.; Sanz-Vergel, A.I.; Cheung, G.W. Rested, friendly, and engaged: The role of daily positive collegial interactions at work. *Journal of Organizational Behavior* **2017**, *38*, 1213-1226, doi:10.1002/job.2197.
162. Messman, B.A.; Slavish, D.C.; Dietch, J.R.; Jenkins, B.N.; ten Brink, M.; Taylor, D.J. Associations between daily affect and sleep vary by sleep assessment type: What can ambulatory EEG add to the picture? *Sleep Health* **2021**, *7*, 219-228, doi:10.1016/j.sleh.2020.11.009.
163. Mousavi, Z.A.; Lai, J.; Simon, K.; Rivera, A.P.; Yunusova, A.; Hu, S.; Labbaf, S.; Jafarlou, S.; Dutt, N.D.; Jain, R.C.; et al. Sleep Patterns and Affect Dynamics Among College Students During the COVID-19 Pandemic: Intensive Longitudinal Study. *JMIR Formative Research* **2022**, *6*, e33964, doi:10.2196/33964.
164. Ryuno, H.; Yamaguchi, Y.; Greiner, C. Effect of Employment Status on the Association Among Sleep, Care Burden, and Negative Affect in Family Caregivers. *Journal of Geriatric Psychiatry and Neurology* **2021**, *34*, 574-581, doi:10.1177/0891988720957099.
165. Sayre, G.M.; Grandey, A.A.; Almeida, D.M. Does sleep help or harm managers' perceived productivity? Trade-offs between affect and time as resources. *Journal of Occupational Health Psychology* **2021**, *26*, 127-141, doi:10.1037/ocp0000192.
166. Shen, L.; Wiley, J.F.; Bei, B. Sleep and affect in adolescents: Bidirectional daily associations over 28-day ecological momentary assessment. *Journal of Sleep Research* **2022**, *31*, e13491, doi:10.1111/jsr.13491.
167. Shi, X.; Wang, X. Daily spillover from home to work: the role of workplace mindfulness and daily customer mistreatment. *International Journal of Contemporary Hospitality Management* **2022**, *34*, 3008-3028, doi:10.1108/ijchm-10-2021-1281.
168. Shi, S.; Zuo, K.; Xu, W. Feeling better or not: Adjusting affective style moderates the association between sleep duration and positive affect on next day. *PsyCh Journal* **2021**, *10*, 905-915, doi:10.1002/pchj.474.
169. Simor, P.; Krietsch, K.N.; Koteles, F.; McCrae, C.S. Day-to-Day Variation of Subjective Sleep Quality and Emotional States Among Healthy University Students--a 1-Week Prospective Study. *International Journal of Behavioral Medicine* **2015**, *22*, 625-634, doi:10.1007/s12529-015-9464-4.
170. Sin, N.L.; Almeida, D.M.; Crain, T.L.; Kossek, E.E.; Berkman, L.F.; Buxton, O.M. Bidirectional, Temporal Associations of Sleep with Positive Events, Affect, and Stressors in Daily Life Across a Week. *Annals of Behavioral Medicine* **2017**, *51*, 402-415, doi:10.1007/s12160-016-9864-y.
171. Song, B.; Wang, B.; Qian, J.; Zhang, Y. Procrastinate at work, sleep badly at night: How job autonomy matters. *Applied Psychology* **2021**, *71*, 1407-1427, doi:10.1111/apps.12363.
172. Takano, K.; Sakamoto, S.; Tanno, Y. Repetitive thought impairs sleep quality: an experience sampling study. *Behavior Therapy* **2014**, *45*, 67-82, doi:10.1016/j.beth.2013.09.004.

173. Wang, M.T.; Henry, D.A.; Scanlon, C.L.; Del Toro, J.; Voltin, S.E. Adolescent Psychosocial Adjustment during COVID-19: An Intensive Longitudinal Study. *Journal of Clinical Child & Adolescent Psychology* **2023**, *52*, 633-648, doi:10.1080/15374416.2021.2007487.
174. Wang, Y.R.; Black, K.J.; Martin, A. Antecedents and outcomes of daily anticipated stress and stress forecasting errors. *Stress Health* **2021**, *37*, 898-913, doi:10.1002/smi.3044.
175. Wong, P.M.; Hasler, B.P.; Kamarck, T.W.; Wright, A.G.C.; Hall, M.; Carskadon, M.A.; Gao, L.; Manuck, S.B. Day-to-day associations between sleep characteristics and affect in community dwelling adults. *Journal of Sleep Research* **2021**, *30*, e13297, doi:10.1111/jsr.13297.
176. Zohar, D.; Tzischinsky, O.; Epstein, R.; Lavie, P. The effects of sleep loss on medical residents' emotional reactions to work events: a cognitive-energy model. *Sleep* **2005**, *28*, 47-54, doi:10.1093/sleep/28.1.47.
177. O'Neill, A.S.; Mohr, C.D.; Bodner, T.E.; Hammer, L.B. Perceived partner responsiveness, pain, and sleep: A dyadic study of military-connected couples. *Health Psychology* **2020**, *39*, 1089-1099, doi:10.1037/hea0001035.
178. Baglioni, C.; Johann, A.F.; Benz, F.; Steinmetz, L.; Meneo, D.; Frase, L.; Kuhn, M.; Ohler, M.; Huart, S.; Speiser, N.; et al. Interactions between insomnia, sleep duration and emotional processes: An ecological momentary assessment of longitudinal influences combining self-report and physiological measures. *Journal of Sleep Research* **2024**, *33*, doi:10.1111/jsr.14001.
179. Chachos, E.; Shen, L.; Yap, Y.; Maskevich, S.; Stone, J.E.; Wiley, J.F.; Bei, B. Vulnerability to sleep-related affective disturbances? A closer look at dysfunctional beliefs and attitudes about sleep as a moderator of daily sleep-affect associations in young people. *Sleep Health* **2023**, *26*, doi:10.1016/j.sleh.2023.07.008.
180. Hachenberger, J.; Li, Y.M.; Lemola, S. Physical activity, sleep and affective wellbeing on the following day: An experience sampling study. *Journal of Sleep Research* **2023**, *32*, 1-10, doi:10.1111/jsr.13723.
181. Ohana, M.; Fortin, M. Half Just or Half Unjust? the Influence of Dispositional Optimism on the Link Between Interpersonal Peer Injustice, Negative Emotions and Sleep Problems. *Group and Organization Management* **2023**, *0*, 01461672231193800, doi:10.1177/10596011231200550.
182. Rea, E.M.; DeCarlo Santiago, C.; Nicholson, L.; Heard Egbert, A.; Bohnert, A.M. Sleep, Affect, and Emotion Reactivity in First-Year College Students: A Daily Diary Study. *International Journal of Behavioral Medicine* **2023**, *30(5)*, 753-768, doi:10.1007/s12529-022-10127-0.
183. Sheppard, L.D.; Loi, T.I.; Kmec, J.A. Too Tired to Lean In? Sleep Quality Impacts Women's Daily Intentions to Pursue Workplace Status. *Sex Roles* **2022**, *87*, 379-389, doi:10.1007/s11199-022-01321-1.
184. Xie, M.; Zhang, Y.; Wang, W.; Chen, H.; Lin, D. Associations Between Multiple Dimensions of Sleep and Mood During Early Adolescence: A Longitudinal Daily Diary Study. *Journal of Youth and Adolescence* **2024**, doi:10.1007/s10964-024-02007-5.
185. Kroenke, K.; Spitzer, R.L.; Williams, J.B.; Lowe, B. An ultra-brief screening scale for anxiety and depression: the PHQ-4. *Psychosomatics* **2009**, *50*, 613-621, doi:10.1176/appi.psy.50.6.613.
186. Peltz, J.; Rogge, R. Adolescent and parent sleep quality mediates the impact of family processes on family members' psychological distress. *Sleep Health* **2022**, *8*, 73-81, doi:10.1016/j.sleh.2021.08.009.

187. Peltz, J.S.; Rogge, R.D.; O'Connor, T.G. Adolescent sleep quality mediates family chaos and adolescent mental health: A daily diary-based study. *Journal of Family Psychology* **2019**, *33*, 259-269, doi:10.1037/fam0000491.
188. Cella, D.; Riley, W.; Stone, A.; Rothrock, N.; Reeve, B.; Yount, S.; Amtmann, D.; Bode, R.; Buysse, D.; Choi, S.; et al. The Patient-Reported Outcomes Measurement Information System (PROMIS) developed and tested its first wave of adult self-reported health outcome item banks: 2005-2008. *Journal of Clinical Epidemiology* **2010**, *63*, 1179-1194, doi:10.1016/j.jclinepi.2010.04.011.
189. Hruska, B.; Anderson, L.; Barduhn, M.S. Multilevel analysis of sleep quality and anger in emergency medical service workers. *Sleep Health* **2022**, *8*, 303-310, doi:10.1016/j.sleh.2022.02.005.
190. Mata, J.; Thompson, R.J.; Jaeggi, S.M.; Buschkuehl, M.; Jonides, J.; Gotlib, I.H. Walk on the bright side: physical activity and affect in major depressive disorder. *Journal of Abnormal Psychology* **2012**, *121*, 297-308, doi:10.1037/a0023533.
191. Flueckiger, L.; Lieb, R.; Meyer, A.H.; Witthauer, C.; Mata, J. Day-to-day variations in health behaviors and daily functioning: two intensive longitudinal studies. *Journal of Behavioral Medicine* **2017**, *40*, 307-319, doi:10.1007/s10865-016-9787-x.
192. McNair, D.; Lorr, M.; Droppleman, L. Profile of mood states manual (rev.). *San Diego: Educational and Industrial Testing Service* **1992**.
193. Shacham, S. A shortened version of the Profile of Mood States. *Journal of Personality Assessment* **1983**, *47*, 305-306, doi:10.1207/s15327752jpa4703_14.
194. Chiang, J.J.; Kim, J.J.; Almeida, D.M.; Bower, J.E.; Dahl, R.E.; Irwin, M.R.; McCreath, H.; Fuligni, A.J. Sleep Efficiency Modulates Associations Between Family Stress and Adolescent Depressive Symptoms and Negative Affect. *Journal of Adolescent Health* **2017**, *61*, 501-507, doi:10.1016/j.jadohealth.2017.04.011.
195. Fuligni, A.J.; Hardway, C. Daily Variation in Adolescents' Sleep, Activities, and Psychological Well-Being. *Journal of Research on Adolescence* **2006**, *16*, 353-378, doi:10.1111/j.1532-7795.2006.00498.x.
196. Lev Ari, L.; Shulman, S. Sleep, daily activities, and their association with mood states among emerging adults. *Biological Rhythm Research* **2013**, *44*, 353-367, doi:10.1080/09291016.2012.692251.
197. Slavish, D.C.; Sliwinski, M.J.; Smyth, J.M.; Almeida, D.M.; Lipton, R.B.; Katz, M.J.; Graham-Engeland, J.E. Neuroticism, rumination, negative affect, and sleep: Examining between- and within-person associations. *Personality and Individual Differences* **2018**, *123*, 217-222, doi:10.1016/j.paid.2017.11.023.
198. Talbot, L.S.; Stone, S.; Gruber, J.; Hairston, I.S.; Eidelman, P.; Harvey, A.G. A test of the bidirectional association between sleep and mood in bipolar disorder and insomnia. *Journal of Abnormal Psychology* **2012**, *121*, 39-50, doi:10.1037/a0024946.
199. Yip, T.; Xie, M.; Cham, H.; El Sheikh, M. Linking ethnic/racial discrimination to adolescent mental health: Sleep disturbances as an explanatory pathway. *Child Development* **2022**, *93*, 973-994, doi:10.1111/cdev.13747.
200. Master, L.; Nahmod, N.G.; Mathew, G.M.; Hale, L.; Chang, A.M.; Buxton, O.M. Why so slangry (sleepy and angry)? Shorter sleep duration and lower sleep efficiency predict worse next-day mood in adolescents. *Journal of Adolescence* **2023**, *95*, 1140-1151, doi:10.1002/jad.12182.

201. McGowan, A.L.; Boyd, Z.M.; Kang, Y.; Bennett, L.; Mucha, P.J.; Ochsner, K.N.; Bassett, D.S.; Falk, E.B.; Lydon-Staley, D.M. Within-person temporal associations among self-reported physical activity, sleep, and well-being in college students. *Psychosomatic Medicine* **2023**, *85*, 141-153, doi:10.1097/PSY.0000000000001159.
202. Russell, J.A. A circumplex model of affect. *Journal of Personality and Social Psychology* **1980**, *39*, 1161, doi:10.1037/h0077714.
203. Diener, E.; Wirtz, D.; Tov, W.; Kim-Prieto, C.; Choi, D.-w.; Oishi, S.; Biswas-Diener, R. New Well-being Measures: Short Scales to Assess Flourishing and Positive and Negative Feelings. *Social Indicators Research* **2009**, *97*, 143-156, doi:10.1007/s11205-009-9493-y.
204. Dickens, C.N.; Gray, A.L.; Heshmati, S.; Oravecz, Z.; Brick, T.R. Daily Implications of Felt Love for Sleep Quality. *The American Journal of Psychology* **2021**, *134*, 463-477, doi:10.5406/amerjpsyc.134.4.0463.
205. Totterdell, P.; Reynolds, S.; Parkinson, B.; Briner, R.B. Associations of sleep with everyday mood, minor symptoms and social interaction experience. *Sleep* **1994**, *17*, 466-475, doi:10.1093/sleep/17.5.466.
206. Jones, F.; Fletcher, B. Taking work home: A study of daily fluctuations in work stressors, effects on moods and impacts on marital partners. *Journal of Occupational and Organizational Psychology* **1996**, *69*, 89-106, doi:10.1111/j.2044-8325.1996.tb00602.x.
207. Tzischinsky, O.; Zohar, D.; Epstein, R.; Chillag, N.; Lavie, P. Daily and yearly burnout symptoms in Israeli shift work residents. *Journal of Human Ergology* **2001**, *30*, 357-362, doi:10.11183/jhe1972.30.357.
208. de Wild-Hartmann, J.A.; Wichers, M.; van Bemmel, A.L.; Derom, C.; Thiery, E.; Jacobs, N.; van Os, J.; Simons, C.J. Day-to-day associations between subjective sleep and affect in regard to future depression in a female population-based sample. *British Journal of Psychiatry* **2013**, *202*, 407-412, doi:10.1192/bjp.bp.112.123794.
209. Wrzus, C.; Wagner, G.G.; Riediger, M. Feeling good when sleeping in? Day-to-day associations between sleep duration and affective well-being differ from youth to old age. *Emotion* **2014**, *14*, 624-628, doi:10.1037/a0035349.
210. van Zundert, R.M.; van Roekel, E.; Engels, R.C.; Scholte, R.H. Reciprocal associations between adolescents' night-time sleep and daytime affect and the role of gender and depressive symptoms. *Journal of Youth and Adolescence* **2015**, *44*, 556-569, doi:10.1007/s10964-013-0009-3.
211. Konen, T.; Dirk, J.; Leonhardt, A.; Schmiedek, F. The interplay between sleep behavior and affect in elementary school children's daily life. *Journal of Experimental Child Psychology* **2016**, *150*, 1-15, doi:10.1016/j.jecp.2016.04.003.
212. Tavernier, R.; Choo, S.B.; Grant, K.; Adam, E.K. Daily affective experiences predict objective sleep outcomes among adolescents. *Journal of Sleep Research* **2016**, *25*, 62-69, doi:10.1111/jsr.12338.
213. Lauritsen, L.; Andersen, L.; Olsson, E.; Sondergaard, S.R.; Norregaard, L.B.; Loventoft, P.K.; Svendsen, S.D.; Frokjaer, E.; Jensen, H.M.; Hageman, I.; et al. Usability, Acceptability, and Adherence to an Electronic Self-Monitoring System in Patients With Major Depression Discharged From Inpatient Wards. *Journal of Medical Internet Research* **2017**, *19*, e123, doi:10.2196/jmir.6673.

214. Cox, R.C.; Sterba, S.K.; Cole, D.A.; Upender, R.P.; Olatunji, B.O. Time of day effects on the relationship between daily sleep and anxiety: An ecological momentary assessment approach. *Behaviour Research and Therapy* **2018**, *111*, 44-51, doi:10.1016/j.brat.2018.09.008.
215. Kalmbach, D.A.; Fang, Y.; Arnedt, J.T.; Cochran, A.L.; Deldin, P.J.; Kaplin, A.I.; Sen, S. Effects of Sleep, Physical Activity, and Shift Work on Daily Mood: a Prospective Mobile Monitoring Study of Medical Interns. *Journal of General Internal Medicine* **2018**, *33*, 914-920, doi:10.1007/s11606-018-4373-2.
216. Li, H.; Mukherjee, D.; Krishnamurthy, V.B.; Millett, C.; Ryan, K.A.; Zhang, L.; Saunders, E.F.H.; Wang, M. Use of ecological momentary assessment to detect variability in mood, sleep and stress in bipolar disorder. *BMC Research Notes* **2019**, *12*, 791, doi:10.1186/s13104-019-4834-7.
217. Merikangas, K.R.; Swendsen, J.; Hickie, I.B.; Cui, L.; Shou, H.; Merikangas, A.K.; Zhang, J.; Lamers, F.; Crainiceanu, C.; Volkow, N.D.; et al. Real-time Mobile Monitoring of the Dynamic Associations Among Motor Activity, Energy, Mood, and Sleep in Adults With Bipolar Disorder. *JAMA Psychiatry* **2019**, *76*, 190-198, doi:10.1001/jamapsychiatry.2018.3546.
218. Parsey, C.M.; Schmitter-Edgecombe, M. Using Actigraphy to Predict the Ecological Momentary Assessment of Mood, Fatigue, and Cognition in Older Adulthood: Mixed-Methods Study. *JMIR Aging* **2019**, *2*, e11331, doi:10.2196/11331.
219. Triantafillou, S.; Saeb, S.; Lattie, E.G.; Mohr, D.C.; Kording, K.P. Relationship Between Sleep Quality and Mood: Ecological Momentary Assessment Study. *JMIR Mental Health* **2019**, *6*, e12613, doi:10.2196/12613.
220. Sin, N.L.; Wen, J.H.; Klaiber, P.; Buxton, O.M.; Almeida, D.M. Sleep duration and affective reactivity to stressors and positive events in daily life. *Health Psychology* **2020**, *39*, 1078-1088, doi:10.1037/hea0001033.
221. Wen, X.; An, Y.; Li, W.; Du, J.; Xu, W. How could physical activities and sleep influence affect inertia and affect variability? Evidence based on ecological momentary assessment. *Current Psychology* **2020**, *41*, 3055-3061, doi:10.1007/s12144-020-00803-3.
222. Difrancesco, S.; Penninx, B.; Antypa, N.; van Hemert, A.M.; Riese, H.; Lamers, F. The day-to-day bidirectional longitudinal association between objective and self-reported sleep and affect: An ambulatory assessment study. *Journal of Affective Disorders* **2021**, *283*, 165-171, doi:10.1016/j.jad.2021.01.052.
223. Fang, Y.; Forger, D.B.; Frank, E.; Sen, S.; Goldstein, C. Day-to-day variability in sleep parameters and depression risk: a prospective cohort study of training physicians. *NPJ Digital Medicine* **2021**, *4*, 28, doi:10.1038/s41746-021-00400-z.
224. Lee, S. Naturally Occurring Consecutive Sleep Loss and Day-to-Day Trajectories of Affective and Physical Well-Being. *Annals of Behavioral Medicine* **2022**, *56*, 393-404, doi:10.1093/abm/kaab055.
225. Minaeva, O.; George, S.V.; Kuranova, A.; Jacobs, N.; Thiery, E.; Derom, C.; Wichers, M.; Riese, H.; Booij, S.H. Overnight affective dynamics and sleep characteristics as predictors of depression and its development in women. *Sleep* **2021**, *44*, doi:10.1093/sleep/zsab129.
226. Neubauer, A.B.; Kramer, A.C.; Schmidt, A.; Könen, T.; Dirk, J.; Schmiedek, F. Reciprocal relations of subjective sleep quality and affective well-being in late childhood. *Developmental Psychology* **2021**, *57*, 1372-1386, doi:10.1037/dev0001209.

227. Simor, P.; Polner, B.; Bathori, N.; Sifuentes-Ortega, R.; Van Roy, A.; Albajara Saenz, A.; Luque Gonzalez, A.; Benkirane, O.; Nagy, T.; Peigneux, P. Home confinement during the COVID-19: day-to-day associations of sleep quality with rumination, psychotic-like experiences, and somatic symptoms. *Sleep* **2021**, *44*, 1-15, doi:10.1093/sleep/zsab029.
228. Sin, N.L.; Rush, J.; Buxton, O.M.; Almeida, D.M. Emotional Vulnerability to Short Sleep Predicts Increases in Chronic Health Conditions Across 8 Years. *Annals of Behavioral Medicine* **2021**, *55*, 1231-1240, doi:10.1093/abm/kaab018.
229. Sun-Suslow, N.; Campbell, L.M.; Tang, B.; Fisher, A.C.; Lee, E.; Paolillo, E.W.; Heaton, A.; Moore, R.C. Use of digital health technologies to examine subjective and objective sleep with next-day cognition and daily indicators of health in persons with and without HIV. *Journal of Behavioral Medicine* **2022**, *45*, 62-75, doi:10.1007/s10865-021-00233-x.
230. Vigoureux, T.F.D.; Lee, S. Individual and joint associations of daily sleep and stress with daily well-being in hospital nurses: an ecological momentary assessment and actigraphy study. *Journal of Behavioral Medicine* **2021**, *44*, 320-332, doi:10.1007/s10865-021-00207-z.
231. Wieman, S.T.; Arditte Hall, K.A.; MacDonald, H.Z.; Gallagher, M.W.; Suvak, M.K.; Rando, A.A.; Liverant, G.I. Relationships Among Sleep Disturbance, Reward System Functioning, Anhedonia, and Depressive Symptoms. *Behavior Therapy* **2022**, *53*, 105-118, doi:10.1016/j.beth.2021.06.006.
232. Chan, W.S.; Lam, S.C.Y.; Ng, A.S.Y.; Lobo, S. Daily Associations of Sleep Quality and Sleep Duration with Anxiety in Young Adults: The Moderating Effect of Alexithymia. *Behavioral Sleep Medicine* **2022**, *20*, 787-797, doi:10.1080/15402002.2021.2016406.
233. Narmandakh, A.; Oldehinkel, A.J.; Masselink, M.; de Jonge, P.; Roest, A.M. Affect, worry, and sleep: Between- and within-subject associations in a diary study. *Journal of Affective Disorders Reports* **2021**, *4*, doi:10.1016/j.jadr.2021.100134.
234. Parsons, C.E.; Schofield, B.; Batziou, S.E.; Ward, C.; Young, K.S. Sleep quality is associated with emotion experience and adaptive regulation of positive emotion: An experience sampling study. *Journal of Sleep Research* **2022**, *31*, e13533, doi:10.1111/jsr.13533.
235. Sperry, S.H.; Kwapil, T.R. Variability in Sleep Is Associated with Trait-Based and Daily Measures of Bipolar Spectrum Psychopathology. *Behavioral Sleep Medicine* **2022**, *20*, 90-99, doi:10.1080/15402002.2021.1890076.
236. Ying, F.; Wen, J.H.; Klaiber, P.; DeLongis, A.; Slavish, D.C.; Sin, N.L. Associations Between Intraindividual Variability in Sleep and Daily Positive Affect. *Affective Science* **2022**, *3*, 330-340, doi:10.1007/s42761-021-00082-6.
237. Lee, M.H.; Min, A.; Park, C.; Kim, I. How Do Sleep Disturbances Relate to Daytime Functions, Care-related Quality of Life, and Parenting Interactions in Mothers of Children with Autism Spectrum Disorder? *Journal of Autism and Developmental Disorders* **2023**, *53*, 2764-2772, doi:10.1007/s10803-022-05532-z.
238. Marcusson-Clavertz, D.; Sliwinski, M.J.; Buxton, O.M.; Kim, J.; Almeida, D.M.; Smyth, J.M. Relationships between daily stress responses in everyday life and nightly sleep. *Journal of Behavioral Medicine* **2022**, *45*, 518-532, doi:10.1007/s10865-021-00281-3.
239. Patapoff, M.; Ramsey, M.; Titone, M.; Kaufmann, C.N.; Malhotra, A.; Ancoli-Israel, S.; Wing, D.; Lee, E.; Eyler, L.T. Temporal relationships of ecological momentary mood and actigraphy-based sleep measures in bipolar disorder. *Journal of Psychiatric Research* **2022**, *150*, 257-263, doi:10.1016/j.jpsychires.2022.03.055.

240. Roberts, N.A.; Burleson, M.H.; Pituch, K.; Flores, M.; Woodward, C.; Shahid, S.; Todd, M.; Davis, M.C. Affective Experience and Regulation via Sleep, Touch, and "Sleep-Touch" Among Couples. *Affective Science* **2022**, *3*, 353-369, doi:10.1007/s42761-021-00093-3.
241. Kouros, C.D.; Keller, P.S.; Martín-Piñón, O.; El-Sheikh, M. Bidirectional associations between nightly sleep and daily happiness and negative mood in adolescents. *Child Development* **2022**, *93*, e547-e562, doi:10.1111/cdev.13798.
242. Lucke, A.J.; Wrzus, C.; Gerstorf, D.; Kunzmann, U.; Katzorreck, M.; Kolodziejczak, K.; Ram, N.; Hoppmann, C.; Schilling, O.K. Good night-good day? Bidirectional links of daily sleep quality with negative affect and stress reactivity in old age. *Psychology and Aging* **2022**, *37*, 876-890, doi:10.1037/pag0000704.
243. Jordan, D.; Slavish, D.C.; Dietch, J.; Messman, B.; Ruggero, C.; Kelly, K.; Taylor, D.J. Investigating sleep, stress, and mood dynamics via temporal network analysis. *Sleep Medicine* **2023**, *103*, 1-11, doi:10.1016/j.sleep.2023.01.007.
244. Kirshenbaum, J.S.; Coury, S.M.; Colich, N.L.; Manber, R.; Gotlib, I.H. Objective and subjective sleep health in adolescence: Associations with puberty and affect. *Journal of Sleep Research* **2023**, *32*, 1-12, doi:10.1111/jsr.13805.
245. Ng, A.S.C.; Massar, S.A.A.; Bei, B.; Chee, M.W.L. Assessing 'readiness' by tracking fluctuations in daily sleep duration and their effects on daily mood, motivation, and sleepiness. *Sleep Medicine* **2023**, *112*, 30-38, doi:10.1016/j.sleep.2023.09.028.
246. Punna, M.; Sihvonen, S.; Aunola, K.; Rönkä, A. Daily moods, health routines and recovery among employees working in the retail and services sector: A diary study. *International Journal of Social Welfare* **2023**, *32*, 278-290, doi:10.1111/ijsw.12564.
247. Sell, N.T.; Sisson, N.M.; Gordon, A.M.; Stanton, S.C.; Impett, E.A. Daily sleep quality and support in romantic relationships: The role of negative affect and perspective-taking. *Affective Science* **2023**, *4*, 370-384, doi:10.1007/s42761-023-00180-7.
248. Song, J.; Crawford, C.M.; Fisher, A.J. Sleep quality moderates the relationship between daily mean levels and variability of positive affect. *Affective Science* **2023**, doi:10.1007/s42761-022-00177-8.
249. Xie, Y.; Lemay, E.P.; Feeney, B.C. Cyclical Links Between Daily Partner Interactions and Sleep Quality in Older Adult Couples: The Mediating Role of Perceived Partner Responsiveness and Negative Affect. *Personality & Social Psychology Bulletin* **2023**, *0*, 01461672231193800, doi:10.1177/01461672231193800.
250. Collier Villaume, S.; Stephens, J.E.; Craske, M.G.; Zinbarg, R.E.; Adam, E.K. Sleep and daily affect and risk for major depression: Day-to-day and prospective associations in late adolescence and early adulthood. *Journal of Adolescent Health* **2024**, *74*, 388-391, doi:10.1016/j.jadohealth.2023.08.042.
251. Dong, L.; D'Amico, E.J.; Dickerson, D.L.; Brown, R.A.; Palimaru, A.I.; Johnson, C.L.; Troxel, W.M. Bidirectional associations between daily sleep and wake behaviors in Urban American Indian/Alaska Native youth. *Journal of Adolescent Health* **2024**, *74*, 350-357, doi:10.1016/j.jadohealth.2023.08.048.
252. Evans, S.C.; Hamilton, J.L.; Boyd, S.I.; Karlovich, A.R.; Ladouceur, C.D.; Silk, J.S.; Bylsma, L.M. Daily associations between sleep and affect in youth at risk for psychopathology: The moderating role of externalizing symptoms. *Research on Child and Adolescent Psychopathology* **2024**, *52*, 35-50, doi:10.1007/s10802-023-01087-4.

253. Kwan, H.K.; Chen, Y.; Tang, G.; Zhang, X.; Le, J. Power distance orientation alleviates the beneficial effects of empowering leadership on actors' work engagement via negative affect and sleep quality. *Asia Pacific Journal of Management* **2024**, 1-26, doi:10.1007/s10490-024-09947-3.
254. Lee, S.A.; Mukherjee, D.; Rush, J.; Lee, S.; Almeida, D.M. Too little or too much: nonlinear relationship between sleep duration and daily affective well-being in depressed adults. *BMC Psychiatry* **2024**, *24(1)*, doi:10.1186/s12888-024-05747-7.
255. Meigs, J.M.; Kiderman, M.; Kircanski, K.; Cardinale, E.M.; Pine, D.S.; Leibenluft, E.; Brotman, M.A.; Naim, R. Sleepless nights, sour moods: daily sleep-irritability links in a pediatric clinical sample. *Journal of Child Psychology and Psychiatry, and Allied Disciplines* **2024**, doi:10.1111/jcpp.13959.
256. Peng, J.; Wei, Z.; Liu, C.; Li, K.; Wei, X.; Yuan, S.; Guo, Z.; Wu, L.; Feng, T.; Zhou, Y.; et al. Temporal network of experience sampling methodology identifies sleep disturbance as a central symptom in generalized anxiety disorder. *BMC Psychiatry* **2024**, *24(1)*, doi:10.1186/s12888-024-05698-z.
257. Wescott, D.; Taylor, M.; Klevens, A.; Franzen, P.; Roecklein, K. Waking up on the wrong side of the bed: Depression severity moderates daily associations between sleep duration and morning affect. *Journal of Sleep Research* **2024**, *33*, e14010, doi:10.1111/jsr.14010.
258. Zapalac, K.; Miller, M.; Champagne, F.A.; Schnyer, D.M.; Baird, B. The effects of physical activity on sleep architecture and mood in naturalistic environments. *Scientific Reports* **2024**, *14(1)*, 5637, doi:10.1038/s41598-024-56332-7.
